# Distinct resistance mechanisms arise to allosteric vs. ATP-competitive AKT inhibitors

**DOI:** 10.1038/s41467-022-29655-0

**Published:** 2022-04-19

**Authors:** Kristin M. Zimmerman Savill, Brian B. Lee, Jason Oeh, Jie Lin, Eva Lin, Wei-Jen Chung, Amy Young, Wennie Chen, Monika Miś, Kathryn Mesh, Jeffrey Eastham, Florian Gnad, Zhaoshi Jiang, Eric W. Stawiski, Benjamin Haley, Anneleen Daemen, Xiaojing Wang, Hartmut Koeppen, Zora Modrusan, Scott E. Martin, Deepak Sampath, Kui Lin

**Affiliations:** 1 Department of Molecular Oncology, Genentech Inc., South San Francisco, CA USA; 2 Department of Bioinformatics, Genentech Inc., South San Francisco, CA USA; 3 Department of Research Pathology, Genentech Inc., South San Francisco, CA USA; 4 Department of Molecular Biology, Genentech Inc., South San Francisco, CA USA; 5 Department of Discovery Chemistry, Genentech Inc., South San Francisco, CA USA; 6 Department of Microchemistry, Proteomics, Lipidomics and NGS, Genentech Inc., South San Francisco, CA USA; 7 Present Address: Cardinal Health, Dublin, OH USA; 8 Present Address: Loxo Oncology at Lilly, Indianapolis, IN USA; 9 Present Address: Oric Pharmaceutical, South San Francisco, CA USA; 10 Present Address: Roche Diagnostics, Basel, Switzerland; 11 Present Address: BioMap, Inc., Beijing, China; 12 Present Address: PACT Pharma, South San Francisco, CA USA; 13 Present Address: Ultragenyx Pharmaceutical, Inc., Novato, CA USA

**Keywords:** Kinases, Cancer therapy, Prostate cancer, Target identification, Target validation

## Abstract

The AKT kinases have emerged as promising therapeutic targets in oncology and both allosteric and ATP-competitive AKT inhibitors have entered clinical investigation. However, long-term efficacy of such inhibitors will likely be challenged by the development of resistance. We have established prostate cancer models of acquired resistance to the allosteric inhibitor MK-2206 or the ATP-competitive inhibitor ipatasertib following prolonged exposure. While alterations in AKT are associated with acquired resistance to MK-2206, ipatasertib resistance is driven by rewired compensatory activity of parallel signaling pathways. Importantly, MK-2206 resistance can be overcome by treatment with ipatasertib, while ipatasertib resistance can be reversed by co-treatment with inhibitors of pathways including PIM signaling. These findings demonstrate that distinct resistance mechanisms arise to the two classes of AKT inhibitors and that combination approaches may reverse resistance to ATP-competitive inhibition.

## Abstract (web summary)

How resistance to different classes of AKT inhibitors can emerge is unclear. Here, the authors show that resistance to allosteric inhibitors is mainly due to mutation of AKT1 while the ATP competitive resistance is driven by activation of PIM kinases in prostate cancer models.

## Introduction

Enhanced activity of the phosphoinositide 3-kinase (PI3K)/AKT/mechanistic target of rapamycin (mTOR) signaling pathway is among the most frequently observed changes in cancer and is associated with tumor invasiveness, survival, and proliferation^1^. The AKT/PKB serine/threonine kinase functions as a central node in this pathway and is being investigated as a therapeutic target in oncology^2^. Three isoforms of AKT (AKT1, 2 and 3) exist in humans that each contain a Plekstrin homology (PH) domain, a kinase domain, and a C-terminal hydrophobic regulatory region^3^. Activation of these isoforms is mediated by recruitment to PtdIns-3,4-P_2_ (PI3,4P_2_) and PtdIns-3,4,5-P_3_ (PIP_3_) at the plasma membrane and subsequent phosphorylation of T308 and S473 by 3-phosphoinositide-dependent protein kinase 1 (PDPK1) and mTOR complex 2 (mTORC2), respectively. Aberrant activation of AKT in cancer may occur via several mechanisms including mutational activation of the catalytic subunit of PI3K, which generates PIP_3_ and, indirectly, PI3,4P_2_; loss of the PIP_3_ phosphatase PTEN; and, albeit less frequently, activating mutations in AKT^4^. Upon activation, AKT mediates various cellular processes including cell survival, metabolism and proliferation by regulating the activity of downstream proteins including proline-rich AKT substrate of 40 kDa (PRAS40), glycogen synthase kinase 3 (GSK-3), Forkhead box class O (FoxO) transcription factors, tuberous sclerosis complex 2 (TSC2), Bcl-2 associated death promoter (BAD), mTOR complex 1 (mTORC1), eukaryotic translation inhibition factor 4E-binding protein 1 (4EBP1), and the S6 ribosomal protein kinase^4^.

Two main classes of AKT inhibitors (AKTis) have entered clinical investigation in oncology: allosteric inhibitors such as MK-2206 and adenosine 5′-triphosphate (ATP)-competitive inhibitors such as ipatasertib/GDC-0068^2^. Importantly, these inhibitors differentially exploit the on-off activity cycle of AKT. In its inactive state, AKT adopts a closed conformation in which the PH domain interacts with the kinase domain, also referred to as the PH-in state^5,6^. Upon recruitment to the membrane and phosphorylation at T308 and S473, the interaction between the PH and kinase domains is released, resulting in an open PH-out conformation conducive to ATP-binding^5^. Allosteric inhibitors preferentially bind to the inactive PH-in conformation at a cavity formed between the PH and kinase domains, preventing phosphorylation and activation of AKT^5,6^. In contrast, ATP-competitive inhibitors selectively target the PH-out conformation, protecting AKT from dephosphorylation at T308 and S473 while simultaneously blocking ATP binding and kinase activity^7^. As a result, decreased AKT phosphorylation at both T308 and S473 is typically observed in allosteric inhibitor-treated cells while increased or sustained pAKT at both sites is characteristic of the ATP-competitive inhibitors.

Given that intrinsic sensitivity to AKTis such as ipatasertib correlates with AKT pathway activation^8^, the therapeutic potential of AKTis is likely to be the greatest in indications associated with PI3K/AKT pathway activating alterations. One such indication is prostate cancer. Activation of the PI3K/AKT pathway is thought to comprise roughly 50% of metastatic castration-resistant prostate cancer (mCRPC), frequently via PTEN loss^9–11^. In fact, a randomized phase II study evaluating combined inhibition of AKT via ipatasertib and androgen signaling via abiraterone in patients with mCRPC showed superior antitumor activity of the combination compared to abiraterone alone, especially in patients with PTEN-loss tumors^12^. Additionally, in metastatic triple-negative breast cancer (mTNBC), another indication associated with frequent PI3K/AKT pathway activating alterations, the combination of ipatasertib and paclitaxel also improved progression-free survival compared to paclitaxel alone in a randomized phase II trial, with a more pronounced effect observed in patients with *PIK3CA/AKT1/PTEN*-altered tumors^13^. Phase III clinical trials are currently underway to further evaluate ipatasertib as a therapeutic agent in these indications.

While treatment of tumors with targeted therapies can initially result in impressive clinical outcomes, resistance is likely to emerge over extended treatment times^14^. Although some mechanisms of intrinsic resistance to PI3K/AKT/mTOR pathway inhibitors have been described, including SGK1 signaling in breast cancer^15^, Wnt-β-catenin signaling in colon cancer^16^, androgen receptor signaling in prostate cancer^17^ and RAS/RAF pathway signaling across multiple cancers^8^, few studies have explored mechanisms of acquired resistance to AKTis following prolonged treatment. Further, it remains unknown whether overlapping or distinct mechanisms of resistance will arise to long-term treatment with allosteric vs. ATP-competitive inhibitors. Here, we aim to identify mechanisms of acquired resistance to both allosteric and ATP-competitive AKTis using an unbiased approach. We performed systematic analysis of cell lines with acquired AKTi-resistance (AKTi-R) using methods including RNA sequencing (RNA-seq) and whole exome sequencing (exome-seq) and explored potential functional dependencies and combination strategies using a chemical genetics screen. Our findings indicate that distinct mechanisms do arise to the two different classes of AKTis and that combination approaches may be taken to reverse this resistance.

## Results

### Cells resistant to allosteric vs. ATP-competitive AKTis display distinct phenotypes

**Fig. 1:**
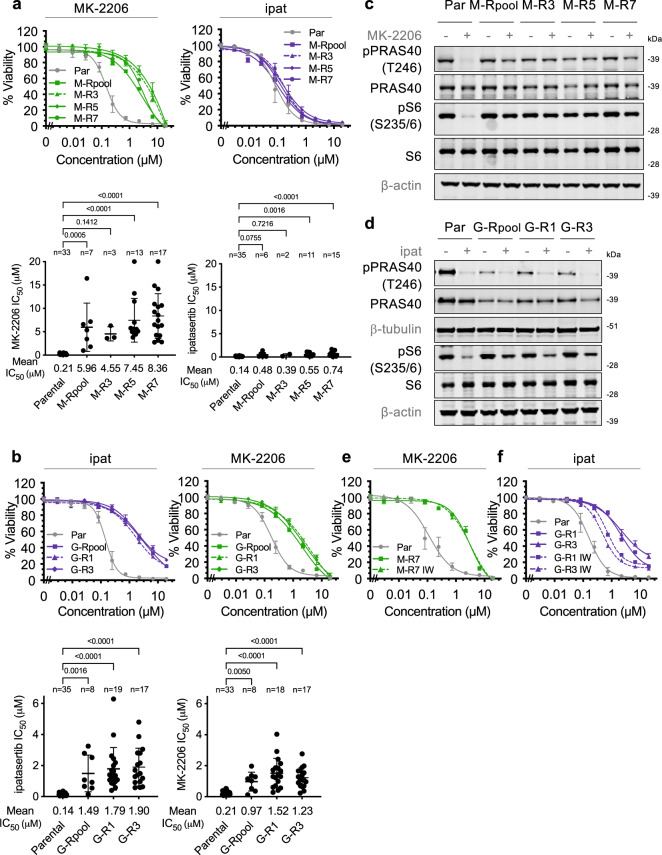
Characteristics of allosteric vs. ATP-competitive AKTi-R cells. **a**, **b** Representative MK-2206 or ipatasertib (ipat) dose response curves (*n* = 4 replicates) and scatter plots depicting absolute IC_50_ values from independent experiments from a 4-day viability assay with LNCaP parental (Par) or AKTi-R cell lines. Error bars represent standard deviation (SD). *P* values compared to Par are indicated using ordinary one-way ANOVA corrected for multiple comparisons using Dunnett’s test. **c,d** Immunoblot analysis of indicated total or phosphorylated proteins in cells treated with DMSO or 5 μM AKTi for 3 h. **e**, **f** Withdrawal of AKTi for 11 passages (IW) was performed in both M-R and G-R cells. The response of Par, M-R or G-R, and M-R IW or G-R IW cells to AKT inhibition was then assessed as in **a**, **b**. See also Supplementary Figs. 1, 2. Source data are provided as a Source Data file.

To establish cell lines with acquired resistance both to ipatasertib/GDC-0068 and to MK-2206, we started with the LNCaP prostate cancer cell line, which is PTEN-deficient and intrinsically sensitive to both AKTis with similar IC_50_s (Supplementary Fig. 1a, b). Resistance was established by treatment of the parental (Par) LNCaP cells with gradually escalating doses of each AKTi up to 5 μM. Surviving cell pools and clones were maintained in the presence of AKTi and subjected to various analyses (Supplementary Fig. 1c). Assessment of viability revealed that the MK-2206-resistant cells (M-R) display substantial resistance specifically to the allosteric inhibitor (Fig. 1a). Conversely, ipatasertib/GDC-0068-resistant cells (G-R) are resistant to both the allosteric and ATP-competitive AKTi (Fig. 1b). The degree of resistance of the resistant pools and individual clones is comparable for both M-R and G-R cells. Immunoblot analysis revealed that while pAKT is partially suppressed in M-R cells in the presence of 5 μM MK-2206, a low level of pAKT persists (Supplementary Fig. 1d, e). This corresponds to inefficient suppression of the phosphorylation of its direct substrates PRAS40 and GSK-3β, as well as the downstream targets of mTORC1 ribosomal protein S6 and 4EBP1 in M-R cells (Fig. 1c, Supplementary Fig. 1d–f). Conversely, while ipatasertib-mediated suppression of signaling downstream of mTORC1 (as indicated by phosphorylation of 4EBP1 and S6) and apoptosis (measured by cleaved PARP levels) are similarly ineffective in G-R cells, AKT signaling to its direct substrates (measured by phosphorylation of its direct substrates PRAS40 and GSK-3β) is still largely impaired by ipatasertib in G-R cells (Fig. 1d, Supplementary Fig. 1d–f), suggesting that AKT-independent mechanisms can sustain mTORC1 downstream signaling in G-R cells. Interestingly, a decrease in phosphorylated PRAS40 was detected in G-R cells in the absence of ipatasertib, corresponding with a decrease in total PRAS40 levels (Fig. 1d, Supplementary Fig. 1d–f). This decrease in pPRAS40 persists even after withdrawal of ipatasertib from the G-R cells for 11 passages (IW) (Supplementary Fig. 1e). As described in subsequent sections, this corresponds with a truncating mutation in the *AKT1S1* gene encoding PRAS40. Withdrawal of the inhibitor from the resistant lines for 11 passages followed by re-treatment with the AKTis revealed that while the resistance of M-R cells is not reversible, partial reversion of resistance can be observed in G-R cells (Fig. 1e, f, Supplementary Fig. 2a–d). The partial reversion of resistance in G-R IW cells is associated with a restored ability of ipatasertib to suppress mTORC1 signaling (Supplementary Fig. 2e). Altogether, these findings indicate that the resistance of M-R cells is specific to allosteric AKT inhibition, irreversible, and associated with impaired MK-2206-mediated suppression of AKT signaling while the resistance of G-R cells is AKTi class-independent, partially reversible, and associated with suppression of AKT but not mTORC1 signaling by ipatasertib. Thus, distinct mechanisms apparently drive the resistance of M-R and G-R cells.

### Acquired alterations in AKT isoforms mediate MK-2206 resistance of M-R cells

**Fig. 2:**
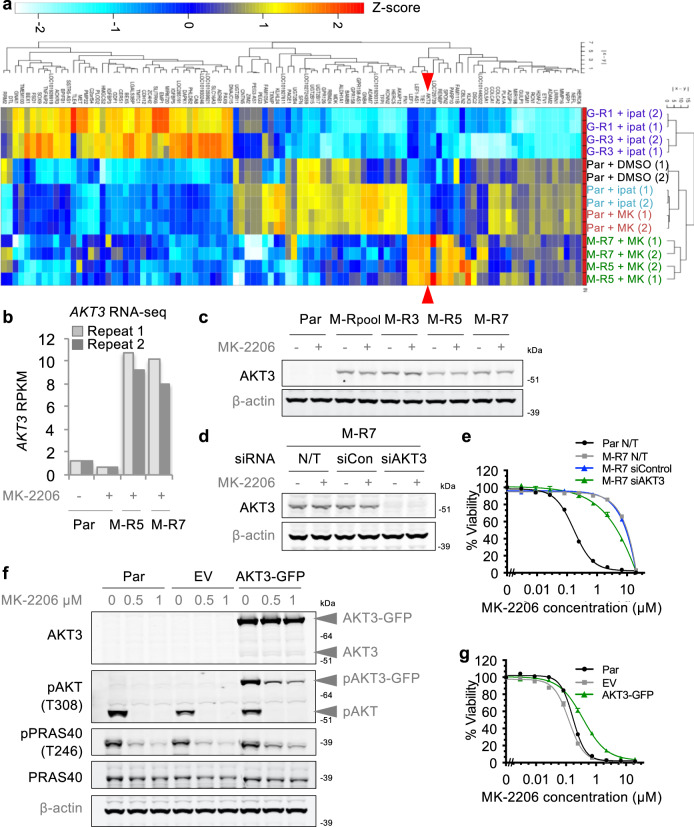
Increased AKT3 modestly impacts the response of LNCaP Par or M-R cells to MK-2206. **a** Hierarchical clustering and heatmap of RNA-seq transcriptome analysis for top 100 variably expressed genes in AKTi-treated (5 μM, 14 h) M-R or G-R cells vs. Par cells (-/+ AKTi) are displayed. Color corresponds to per-gene z-score. Red arrowheads indicate *AKT3* expression. Data represent 2 biological replicates. **b** RPKM values associated with the *AKT3* locus in various cell lines are plotted. **c** Immunoblot analysis of AKT3 in indicated cell lines treated as in Fig. 1c. **d** Cells were either not transfected (N/T) or transfected with siRNA targeting a random scrambled sequence (siControl) or *AKT3* (siAKT3), cultured for 48 h, and subjected to a second round of transfection. The next day, cells were treated with 5 μM MK-2206 or DMSO for 3 h and analyzed by immunoblot. **e** As in **d** except cell viability was assessed 4 days after addition of MK-2206. Data are presented as Mean ± SEM; *n* = 4 replicates. **f** Immunoblot analysis of LNCaP Par cells or those stably expressing empty vector (EV) or AKT3-GFP treated with DMSO or indicated concentrations of MK-2206 for 3 h. Grey arrow heads denote AKT3 or pAKT bands associated with molecular weights predicted for endogenous or GFP-tagged AKT3. **g** Response of Par, EV, or AKT3-GFP cells to MK-2206 was assessed with a 4-day viability assay as in Fig. 1a. Data are presented as Mean ± SEM; *n* = 4 replicates. See also Supplementary Figs. 3, 4 and Supplementary Dataset 1, 2. Source data are provided as a Source Data file.

RNA-seq analysis revealed that a vast number of genes are differentially expressed in AKTi-R cells compared with parental cells (Supplementary Dataset 1). Clustering of the top 100 most variably expressed genes demonstrated that while the MK-2206- and ipatasertib-treated parental cells display similar transcriptional profiles in these genes, the profiles of M-R vs. G-R cells are distinct from one another and from that of either DMSO- or AKTi-treated parental cells (Fig. 2a). Importantly, the profiles of distinct resistant clones generated using the same AKTi and of assay replicates were similar to one another, indicating reproducibility of the transcriptional signatures (Fig. 2a). To identify alterations in gene expression that occur specifically in allosteric vs. ATP-competitive AKTi-R cells, we looked for M-R-specific transcriptional changes. We identified 11 genes including *AKT3* that are upregulated in M-R cells in the presence of MK-2206 in comparison to parental cells treated with either DMSO or MK-2206, and which are not differentially regulated in the G-R cells (Supplementary Fig. 3a). Further examination demonstrated that increased expression of AKT3 can be detected in multiple M-R lines at the level of both mRNA and protein (Fig. 2b, c). This is specific to the AKT3 isoform, as no changes in AKT1 or AKT2 were observed (Supplementary Fig. 3b, c), and is unlikely to result from genetic amplification based on SNP array analysis of copy number (Supplementary Dataset 2 and Supplementary Fig. 3d). Further, increased AKT3 is unlikely to represent a short-term response to MK-2206 treatment as treatment of LNCaP parental cells for up to 72 h fails to increase protein levels (Supplementary Fig. 3e). Importantly, increased AKT3 expression is not reversible in cells grown in the absence of inhibitor for 11 passages (Supplementary Fig. 3f) and hence correlates with the lack of reversibility of resistance (Fig. 1e and Supplementary Fig. 2a, b). Given that MK-2206 is less potent against AKT3 than AKT1 or AKT2 in an enzymatic assay^18^ and that there is functional redundancy between AKT isoforms^4^, we hypothesized that increased AKT3 expression may enable partial escape from inhibition by the allosteric inhibitor, and went on to explore the role of AKT3 expression levels in the response to MK-2206 using knockdown as well as ectopic expression. While siRNA-mediated knockdown of AKT3 results in increased sensitivity of M-R cells to MK-2206 (Fig. 2d, e), this increase is small and dependent upon the M-R cell line examined (Supplementary Fig. 4a, b). Stable overexpression of a GFP-tagged form of AKT3 in LNCaP cells confers reduced sensitivity to MK-2206 compared with parental or empty vector (EV)-expressing cells (Fig. 2f, g and Supplementary Figs. 4c, d). However, even substantial overexpression of AKT3 only minimally impacts the response of cells to MK-2206 and does not recapitulate the degree of resistance observed in M-R cells. Therefore, we conclude that increased AKT3 expression likely plays a minor role in the resistance of M-R cells.

**Fig. 3:**
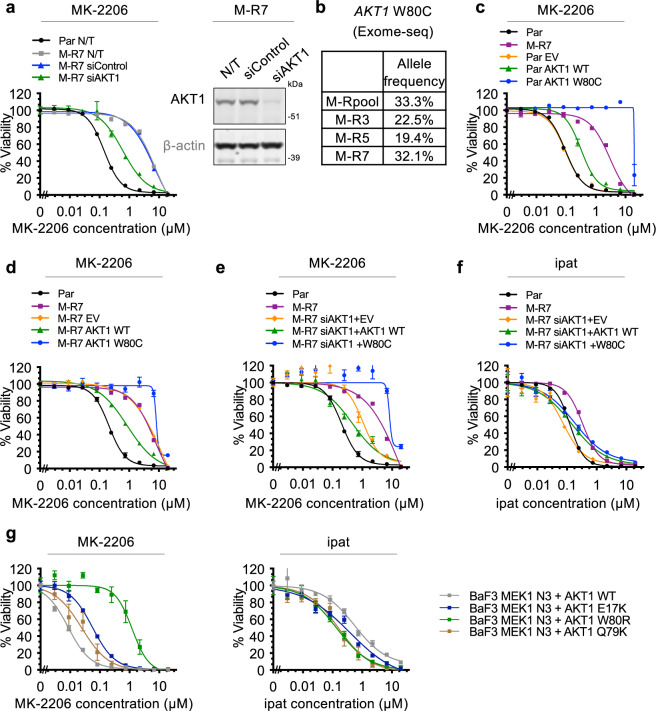
Mutation of W80 on *AKT1* confers resistance to MK-2206 but not ipatasertib. **a** Par or M-R7 cells were either not-transfected (N/T) or transfected with siControl or siRNA targeting *AKT1* (siAKT1) and grown overnight. Cells were then treated with MK-2206 and viability was assessed 4 days later (left). Levels of indicated proteins were assessed by immunoblot 6 days following transfection (right). **b** Allele frequencies of the *AKT1* W80C mutation in individual cell lines, as detected by exome-seq. **c** LNCaP Par, M-R7 or Par cells stably overexpressing cumate-inducible, siRNA-resistant AKT1 WT or W80C, or EV were treated with 10 μg/ml cumate and 4 days later, re-plated in 10 μg/ml cumate. The following day, cells were treated with a dose range of MK-2206 and viability was assessed after a further 4 days. **d** Response of M-R7 cells stably overexpressing cumate-inducible siRNA-resistant AKT1 WT, AKT1 W80C, or EV to MK-2206 was assessed as in **c**. **e** As in **d** except M-R7 EV, AKT1 WT and AKT1 W80C lines were transfected with siAKT1 when re-plating in 10 μg/ml cumate. **f** As in **e** except the response of cells to ipatasertib was assessed. **g** Response of Ba/F3 cells simultaneously overexpressing MEK1 N3 and AKT1 WT, E17K or W80R to MK-2206 (left) or ipatasertib (right) was assessed 4 days after plating cells in the absence of IL-3 with the viability assay. Error bars represent standard error of the mean (SEM); n = 4 replicates in **a**, **c**–**g**. See also Supplementary Fig. 5 and Supplementary Dataset 3, 4. Source data are provided as a Source Data file.

Interestingly, siRNA-mediated depletion of AKT1 results in a more dramatic reversion of resistance in M-R cells (Fig. 3a) than that observed following AKT3 depletion (Fig. 2d, e), suggesting that AKT1 plays a greater role in the resistance of M-R cells to allosteric AKT inhibition. Indeed, exome-seq revealed a heterozygous point mutation (W80C) in the PH domain of AKT1 in all M-R cell lines (Fig. 3b, Supplementary Fig. 5a, Supplementary Dataset 3). This tryptophan residue is reported to play a key role in the formation of a cavity in the inactive conformation of AKT that serves as the binding site of allosteric AKTis and mutagenesis of this residue confers resistance to allosteric inhibitors^5,6,19–21^. To investigate the role of this cysteine substitution, we exploited a piggyBac transposon-based system to stably express cumate-inducible wildtype (WT) or W80C AKT1 (Supplementary Fig. 5b, c). As expected, while overexpression of AKT1 WT in LNCaP parental cells only results in a slight increase in MK-2206 resistance compared with untransfected or EV-expressing cells, cumate-induced expression of AKT1 W80C confers dramatic MK-2206 resistance that even exceeds that observed in M-R cells (Fig. 3c). Overexpression of AKT1 WT in M-R7 cells results in a slight increase in sensitivity to MK-2206, consistent with dilution of the W80C allele by the increased pool of the MK-2206-targetable WT AKT1 allele, while overexpression of W80C in M-R7 further increases resistance to MK-2206 compared to that of non-transfected or EV-expressing M-R7 cells (Fig. 3d). We then asked whether ectopic expression of AKT1 WT or W80C mutant could rescue the resistance to MK-2206 in M-R cells when endogenous AKT1 is depleted. Consistent with Fig. 3a, siRNA-mediated knockdown of endogenous AKT1 alleles (both WT and W80C) results in enhanced sensitivity of M-R cells to MK-2206 (Fig. 3e). As expected, simultaneous overexpression of siRNA-resistant AKT1 W80C, but not WT, rescues resistance to MK-2206 (Fig. 3e). In contrast, these manipulations did not significantly change the sensitivity of M-R cells to ipatasertib (Fig. 3f). These findings strongly suggest that the AKT1 W80C mutation plays a major role in the resistance of M-R cells specifically to allosteric AKT inhibition.

Interestingly, a W80R mutation in *AKT1* has been detected in patients with uterine, colon, and breast cancer, albeit at lower frequencies than the activating mutation E17K (Supplementary Dataset 4). To explore whether this arginine substitution is also associated with resistance to allosteric AKT inhibition, we exploited the IL-3-independent growth assay in the Ba/F3 murine pro-B cell line. Survival of the IL-3-dependent Ba/F3 cells can be rendered IL-3-independent via co-overexpression of AKT1 and an activated form of the MAP2 kinase mitogen-activated protein kinase (MAPK)/extracellular-signal- regulated kinase (ERK) kinase (MEK1) (Mek1 ΔN3, S218E, S222D), termed MEK1 N3^22^. Overexpression of AKT1 W80R in Ba/F3 cells results in an increase in pAKT (S473), pPRAS40 (T246) and pS6 (S235/236) levels comparable to that observed following overexpression of WT AKT1 (Supplementary Fig. 5d). Compared to the strongly transforming E17K activating mutation, AKT1 W80R is only slightly more potent than WT at promoting IL-3-independent growth of Ba/F3-MEK1 N3 cells (Supplementary Fig. 5e), but results in substantially greater resistance to MK-2206-mediated inhibition in comparison to that observed in Ba/F3-MEK1 N3 cells overexpressing similar levels of WT AKT1 (~86-fold increase in IC_50_) (Fig. 3g and Supplementary Fig. 5f). In contrast, similar to observations made following AKT1 W80C overexpression, cells overexpressing AKT1 W80R remain sensitive to the ATP-competitive inhibitor ipatasertib (Fig. 3g and Supplementary Fig. 5f). We also examined a neighboring cancer-associated AKT1 mutation we previously reported^22^, Q79K. This mutation exhibits a stronger transforming activity similar to that of E17K (Supplementary Fig. 5e), and causes mild resistance to MK-2206 while remaining sensitive to ATP-competitive inhibitors in the Ba/F3 assay (Fig. 3g and Supplementary Fig. 5f). The degree of resistance of Q79K to MK-2206 is also more similar to E17K (2-6x), nowhere near W80C ( > 86x). This suggests that although Q79 is next to W80 in the primary sequence, it does not have the same structural impact on allosteric inhibitor binding as W80, which makes key pi-pi interactions with allosteric inhibitors such as MK-2206^5,6^. Therefore, mutation of AKT1 at W80 likely represents a clinically relevant mechanism of resistance specifically to allosteric AKTis.

### PIM signaling promotes acquired resistance to ipatasertib

In contrast to the M-R cells, no alterations in sequence or expression of AKT isoforms were detected in G-R cells by exome-seq or RNA-seq analysis (Supplementary Dataset 1, 3). A heterozygous truncating mutation (Q178*) and a slight decrease in mRNA levels in AKT1 substrate 1 (*AKT1S1*)/PRAS40 were detected in multiple G-R clones (Supplementary Dataset 3 and Supplementary Fig. 6a–c). As mentioned above, these alterations were associated with decreased levels of the total and phosphorylated forms of the WT PRAS40 protein (Fig. 1d, Supplementary Figs. 1d, 6d). As PRAS40 has been proposed to function in many contexts as a negative regulator of mTORC1 activity^23–25^, we hypothesized that reduced levels of WT PRAS40 may enable AKT-independent mTORC1 signaling and proliferation in G-R cells. However, results from shRNA-mediated knockdown, CRISPR-mediated knockout, CRISPR-mediated mutational knock-in and ectopic overexpression of the mutation suggested that alteration of PRAS40 alone is unlikely to drive ipatasertib resistance in LNCaP cells (Supplementary Fig. 6e–h).

**Fig. 4:**
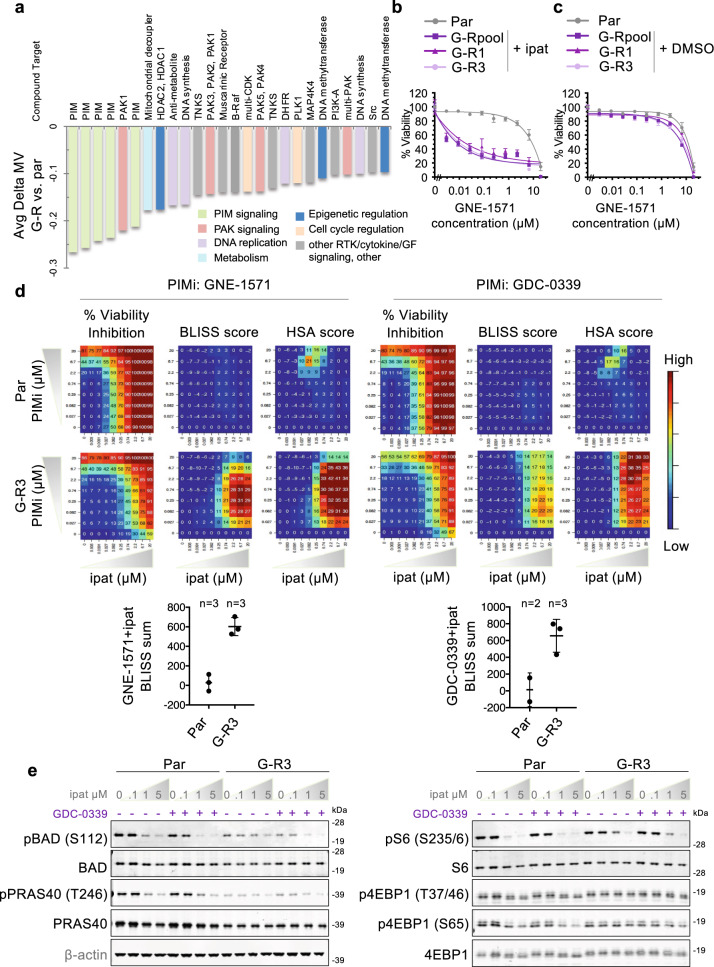
A chemical genetics screen revealed that inhibitors of the PIM kinases reverse the resistance of G-R cells to ipatasertib. **a** Bar plot depicts average mean viability difference between G-R and Par cells (Avg Delta MV) of the chemical genetics screen hits. Compounds with Avg Delta MV ≤  −0.10 are plotted in ascending order. Compound targets are indicated above bar plot. Colors correspond to pathways targeted by each compound as indicated in legend. The mean Delta MV of all compounds screened is −0.01 (see also Supplementary Dataset 5). **b** Response of Par cells plated in DMSO-control medium or G-R cells plated in 5 μM ipatasertib-containing medium to the PIMi GNE-1571 was assessed using a 4-day viability assay. Error bars represent SEM; n = 4 replicates. **c** As in **b** except all cells were plated in DMSO-control medium. **d** Heatmaps depict % viability inhibition, Bliss or HSA scores associated with each dose combination treatment of Par or G-R3 cells with ipatasertib and PIMi (GNE-1571 or GDC-0339). Mean Bliss sum values from independent biological replicates are depicted in scatter plots below. Data are presented as Mean ± SD of the indicated numbers of biological replicates. **e** Indicated proteins were assessed by immunoblot following a 24-hour treatment with the indicated concentrations of ipatasertib and/or 0.1 μM GDC-0339 in Par or G-R3 cells. Representative data from at least 2 independent experiments are shown. See also Supplementary Figs. 6–9 and Supplementary Dataset 5. Source data are provided as a Source Data file.

Given that a high number of alterations in both gene expression and exome sequence were detected in G-R cells compared to parental LNCaP cells (Supplementary Dataset 1, 3) and that multiple alterations could be simultaneously driving resistance, the identification of resistance drivers through functional characterization of each alteration or combination of alterations proved challenging. Instead, we took a chemical genetics screen approach to identify the molecular pathways or mechanisms involved in maintaining the resistance of G-R cells to ipatasertib and to identify potential combination strategies to overcome this resistance. A library of 426 small molecules including chemotherapeutics and compounds targeting a range of molecular mechanisms were screened against parental cells plated in DMSO-containing medium or G-R cells plated in ipatasertib (Supplementary Fig. 7a). Both ipatasertib and MK-2206 were included in the library and as expected, they were identified among the top compounds associated with enhanced resistance in G-R cells (Supplementary Fig. 7b). Compounds with average mean viability (MV, based on the area under the curve of dose-response curves) difference between G-R cells and the parental cells (Delta MV) ≤ −0.10 or those with IC_50_ log2 fold change (FC) $\le$ −1 are plotted in Fig. 4a and Supplementary Fig. 7c, respectively and highlighted in Supplementary Dataset 5. In several cases, multiple compounds with diverse chemical scaffolds targeting the same molecular pathways/mechanisms were among these hits, indicating that the effects observed were unlikely a result of off-target effects.

Remarkably, 5 of the top 6 hits by Delta MV and the top 4 hits by IC_50_ log2 FC were all inhibitors of the proviral integration of Moloney virus (PIM) kinases (Fig. 4a and Supplementary Fig. 7b–d), a family of serine/threonine kinases that function in parallel to AKT to regulate apoptosis, metabolism, and protein translation via phosphorylation of several substrates shared with AKT and mTORC1 including TSC2^26^, PRAS40^27^, BAD^28^, and 4EBP1^29^. Validation studies confirmed that the enhanced sensitivity of G-R cells to PIM inhibition was reproducible across multiple PIM inhibitors and G-R lines (Fig. 4b, Supplementary Fig. 7e, f). These include all 5 PIMis present in the library, with GNE-5652 meeting the Delta MV criteria but not the IC_50_ log2 FC criteria due to <50% maximum inhibition of viability after curve fitting (Supplementary Fig. 7d). Furthermore, AZD1208 and LGH447, PIMis with unrelated chemical structures not included in the original screen, also showed similar enhanced efficacy in G-R cells compared to parental cells (Supplementary Fig. 7f). Importantly, the enhanced sensitivity of G-R cells to PIM inhibition required the presence of ipatasertib (Fig. 4b, c and Supplementary Fig. 7e, f).

Combination effects of PIM inhibition and ipatasertib were further explored in parental and G-R cells using a dose-response matrix followed by analysis of synergy or additivity using the Bliss independence or “highest single agent” (HSA) models, respectively^30–32^. While only weak additivity was observed in parental cells, co-treatment of ipatasertib with PIM inhibitors in G-R cells resulted in a substantial increase in both the Bliss and HSA scores, indicating strong synergy between PIM and AKT inhibition in the G-R cells (Fig. 4d). Given the functional overlap between the PIM and AKT kinases, we hypothesized that PIM signaling may enable the AKT-independent proliferation of G-R cells via the phosphorylation of targets downstream of AKT and mTORC1. In support of this notion, combined treatment with PIM inhibitors and ipatasertib was required to more effectively suppress phosphorylation of BAD and the mTORC1 effectors S6 and 4EBP1 in G-R cells compared with either treatment alone (Fig. 4e and Supplementary Fig. 8a, b). Consistent with persistent mTORC1 signaling driving resistance to ipatasertib, the G-R cells maintained their original sensitivity to the mTORC1 kinase inhibitor RapaLink-1^33^ (Supplementary Fig. 7f). Similar synergistic effects were also observed between MK-2206 and PIM inhibitors in G-R cells, consistent with PIM-dependent activity being the shared mechanism of resistance to both AKTis in G-R cells (Supplementary Fig. 9a).

**Fig. 5:**
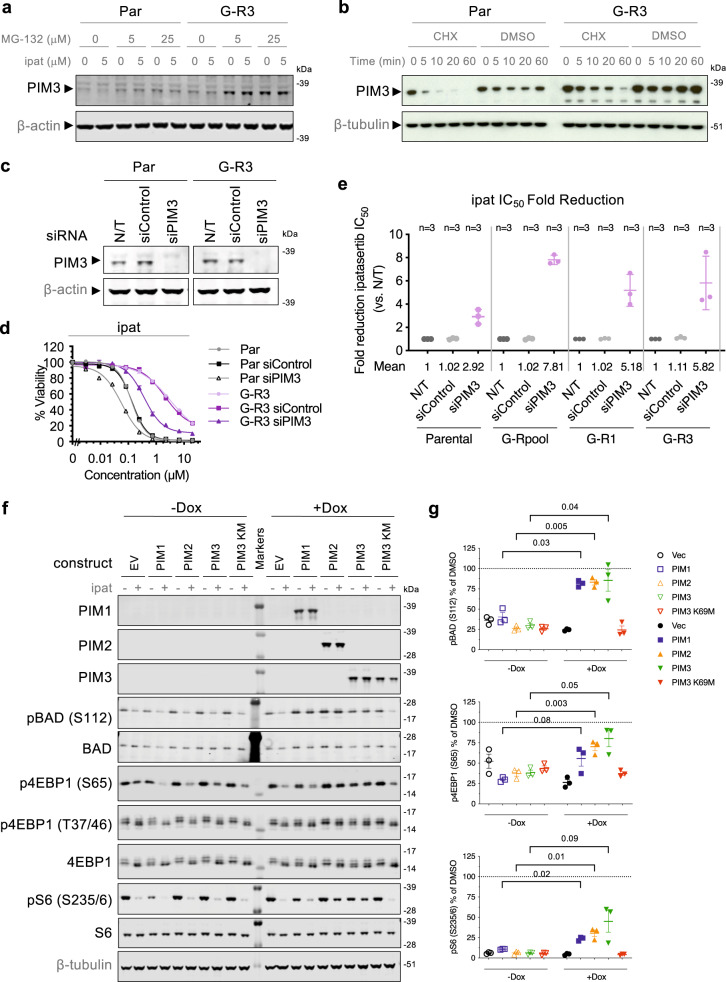
PIM3 is required for the resistance of G-R cells to ipatasertib. **a** Par or G-R3 cells were treated with 5 μM ipatasertib with or without 5 or 25 μM MG-132 for 2 h and indicated protein levels were assessed by immunoblot. **b** Par or G-R3 cells were treated with 50 μg/mL cycloheximide (CHX) or DMSO and PIM3 protein levels were analyzed by immunoblot at the indicated timepoints. **c** PIM3 levels were assessed by immunoblot in non-transfected (N/T), siControl-transfected, or siRNA targeting *PIM3* (siPIM3)-transfected Par or G-R3 cells. **d** Non-transfected, siControl or siPIM3-transfected Par or G-R3 cells were grown overnight and treated with a dose range of ipatasertib. Viability was assessed 4 days later. Error bars represent SEM; *n* = 4 replicates. **e** Scatter plot depicts fold reduction in ipatasertib IC_50_ in cells transfected with siControl or siPIM3 vs. N/T cells. Error bars represent SD; *n* = 3 independent experiments. **f** LNCaP cells stably transfected with Dox-inducible empty vector (EV), PIM1, PIM2, PIM3, or the PIM3 K69M (KM) mutant were cultured in the absence or presence of 100 ng/mL Dox for 3 days, then treated with DMSO or 1 μM ipatasertib for 3 hours and levels of indicated proteins were assessed by immunoblot. **g** Quantification of the indicated phosphoproteins in ipat-treated cells as in **f**, expressed as percentage of each corresponding DMSO-treated cells normalized to β-tubulin (pBAD and p4EBP1) or total S6 (pS6). Error bars represent SEM; *n* = 3 independent experiments; *p* values of the selected pairs are indicated using paired two-tailed *t* test. See also Supplementary Fig. 10. Source data are provided as a Source Data file.

While there are three distinct PIM isoforms (PIM1, 2 and 3), each potently targeted by the pan-PIM kinase inhibitors included in this study, LNCaP cells primarily express PIM3 protein (Supplementary Fig. 10a, b). The PIM kinases have been shown to be constitutively active when expressed and therefore, regulation of activity is thought to be primarily dependent upon expression levels^34–36^. Interestingly, while PIM3 transcript levels are not increased in G-R cells compared with parental cells by RNA-seq analysis (Supplementary Fig. 10c), an increased level of the PIM3 protein can be detected in G-R cells by immunoblots compared to the parental cells with or without ipatasertib treatment (Fig. 5a, b and Supplementary Fig. 10b, d). In addition, ipatasertib treatment induced a down-regulation of PIM3 levels in parental cells while this effect was diminished in G-R3 cells (Fig. 5a and Supplementary Fig. 10b, d, f). Treatment with cycloheximide revealed that the PIM3 protein was rapidly degraded with a half-life of less than 5 min in the parental cells, while the half-life was extended to 15 min in the G-R cells (Fig. 5b and Supplementary Fig. 10g). Treatment with the proteasome inhibitor MG-132, but not lysosome inhibitors chloroquine or bafilomycin A1, resulted in increased PIM3 levels in both parental and G-R cells, suggesting PIM3 is subjected to proteasome-mediated degradation in both cells (Fig. 5a and Supplementary Fig. 10e, f). PIMi treatment also resulted in increased PIM3 levels (Supplementary Fig. 10b), likely due to an inhibition of autophosphorylation-dependent, proteasome-mediated degradation observed in PIM1 and PIM2 previously^37,38^. These results suggest that G-R cells maintain a higher steady-state PIM3 protein expression with a longer half-life than parental cells, and can overcome ipatasertib-induced down-regulation of PIM3. Our data does not rule out the possibility that additional mechanisms, such as increased translational efficiency, may contribute to the increase in PIM3 protein levels.

To more directly explore the role of PIM3 in the response of LNCaP parental and G-R cells to ipatasertib, we performed siRNA knockdown and doxycycline (Dox)-inducible overexpression experiments. While siRNA-mediated depletion of PIM3 resulted in some increase in sensitivity of parental cells to ipatasertib (~2.9-fold decrease in IC_50_ values vs. non-transfected), suggesting that the low level PIM3 expression in the parental cells can antagonize the effect of AKT inhibition to some extent, the impact of PIM3 knockdown on ipatasertib sensitivity was more pronounced in G-R cells (~5.2–7.8-fold decrease in IC_50_ values vs. non-transfected) (Fig. 5c–e). Therefore, depletion of PIM3 has a greater impact on the response of G-R cells to ipatasertib than that of parental cells, mimicking the pattern observed in the inhibitor combination studies. Conversely, inducible overexpression of each of the 3 WT PIM isoforms, but not the kinase-deficient PIM3 K69M mutant, in LNCaP parental cells resulted in ineffective pBAD, p4EBP1 and pS6 inhibition by ipatasertib (Fig. 5f, g). Furthermore, overexpression of PIM isoforms also resulted in reduced sensitivity to ipatasertib in cell viability (Supplementary Fig. 10h). Altogether, these results suggest that PIM signaling plays a major role in the resistance of G-R cells to ipatasertib and that this resistance mechanism can be reversed in vitro by co-treatment with PIM inhibitors.

Among the prostate cancer cell lines characterized in Supplementary Fig. 10a, 22RV1 exhibited significantly higher levels of PIM3 expression than the other lines, while PC-3 showed higher PIM2 expression. AKT activation was observed in PC-3 cells, which is PTEN-null, while 22RV1 and DU145 showed little pAKT activity. Accordingly, ipatasertib exhibits single agent activity in PC-3 cells but not DU145 or 22RV1 cells (Supplementary Figs. 1a, b and 9b). Interestingly, synergy between ipatasertib and PIMi GDC-0339 was observed in 22RV1 cells, suggesting elevated PIM3 expression may also contribute to intrinsic resistance to ipatasertib in this cell line (Supplementary Fig. 9b)

### Combined treatment with a PIMi overcomes resistance to ipatasertib in vivo

**Fig. 6:**
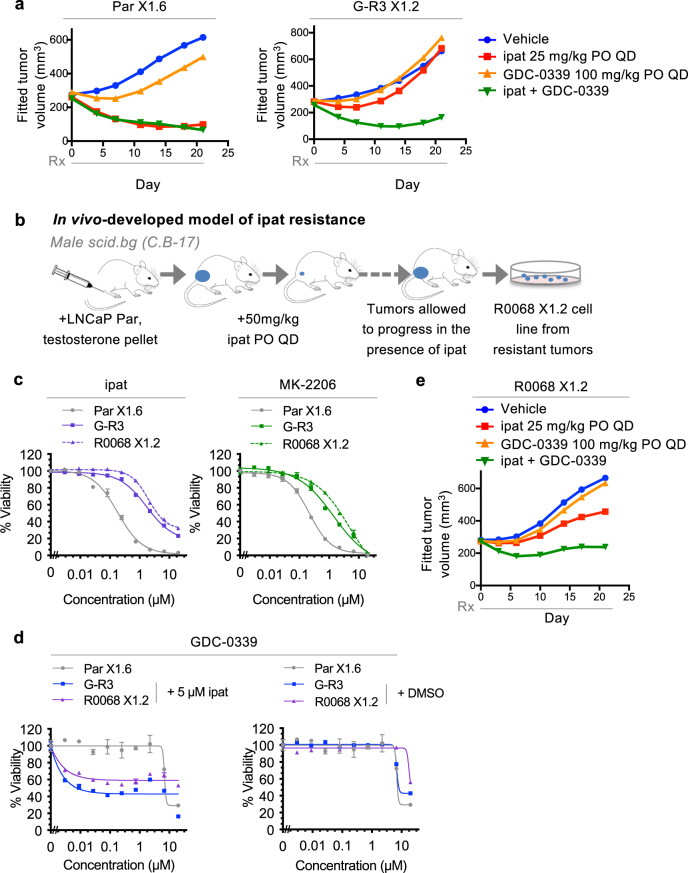
Combined treatment with a PIMi overcomes resistance to ipatasertib in vivo in ipatasertib-resistant models established either in vitro or in vivo. **a** Tumor xenografts derived from LNCaP Par X1.6 or G-R3 X1.2 cells were established by subcutaneous injection into male NOD *scid* gamma (NSG) mice supplemented with testosterone. Mice were treated with indicated inhibitors and tumor volume was monitored over time. Fitted tumor growth curves from 9 mice per group are displayed. **b** Schematic depiction of the establishment of ipatasertib resistance in vivo. **c** Representative AKTi dose response curves from a 4-day viability assay with indicated cell lines. R0068 X1.2 cells (established in vivo) display similar AKTi resistance to that of G-R cells (established in vitro). Error bars represent SEM; *n* = 4 replicates. **d** (left) Response of Par X1.6 cells plated in DMSO-control medium or of G-R3 or R0068 X1.2 cells plated in ipatasertib-containing medium to the PIMi GDC-0339 was assessed using a 4-day viability assay. (right) As in (left) except all cells were plated in DMSO-control medium (Dose response curves for par X1.6 in left and right panels are identical). Error bars represent SEM; *n* = 4 replicates. **e** As in **a** except fitted tumor growth curves from mice bearing R0068 X1.2 xenografts (9 mice per group) are displayed. See also Supplementary Figs. 11–13. Source data are provided as a Source Data file.

Next, we went on to test whether PIM signaling also plays a role in AKTi resistance in an in vivo setting. To this aim, LNCaP parental and G-R3 cells were subjected to in vivo selection to establish sublines that grow consistently as xenografts in immune-compromised male mice supplemented with testosterone. When re-examined in vitro, these sublines, termed Par X1.6 and G-R3 X1.2, remained sensitive or resistant to AKTis, similar to the original lines (Supplementary Fig. 11a, b). Mice bearing Par X1.6 and G-R3 X1.2 tumors were treated with vehicle, ipatasertib, the pan-PIM kinase inhibitor GDC-0339 (which is optimized for its absorption, distribution, metabolism, and excretion (ADME) properties for in vivo dosing^39^), or a combination of ipatasertib and GDC-0339. Tumor growth inhibition was evident following treatment with ipatasertib in Par X1.6 but not G-R3 X1.2 tumor-bearing mice, demonstrating that G-R3 X1.2 retained AKTi resistance in vivo (Fig. 6a and Supplementary Fig. 11c). While neither the Par X1.6 nor the G-R3 X1.2 tumors were responsive to the PIM inhibitor alone, a remarkable combination effect was observed when G-R3 X1.2 xenografts were treated with combined PIM and AKT inhibition (Fig. 6a and Supplementary Fig. 11c, d). The combination was well tolerated with no significant difference in body weight changes between treatment and vehicle groups (Supplementary Fig. 11e). Little combination effect was observed in the Par X1.6 xenograft model. However, since treatment with 25 mg/kg ipatasertib alone is already effective at causing tumor regression in the Par X1.6 tumors, any additional effects of PIM inhibition would be difficult to discern. Immunohistochemistry (IHC) and immunoblot analysis of tumor lysates confirmed elevated PIM3 levels and more effective inhibition of pPRAS40, pS6, p4EBP1, pBAD by the combination treatment in the G-R3 X1.2 tumors, while ipatasertib alone was effective in the Par X1.6 tumors (Supplementary Fig. 12a–c).

Given the possibility that the acquisition of resistance to AKT inhibition may be driven by very different mechanisms following long-term inhibitor exposure in an in vivo vs. in vitro setting, we went on to explore mechanisms of resistance to ipatasertib established in vivo. To this aim, mice bearing LNCaP parental tumors were exposed to prolonged ipatasertib treatment and surviving tumors were excised and adapted in vitro to establish the resistant line R0068 X1.2 (Fig. 6b). In vitro characterization studies revealed that R0068 X1.2 cells display a similar degree of AKTi resistance and reversibility to that of G-R3 cells (Fig. 6c and Supplementary Fig. 13a). Importantly, like the G-R cells, R0068 X1.2 cells display enhanced sensitivity to the PIMi GDC-0339 only when plated in the presence of ipatasertib (Fig. 6d). Similar to the G-R cells established in vitro, elevated levels of PIM3 protein were also observed in the R0068 X1.2 tumor lysates compared to Par X1.6 tumors (Supplementary Fig. 13b) and enhanced inhibition of tumor growth and downstream markers was observed when ipatasertib was combined with GDC-0339 in vivo (Fig. 6e and Supplementary Fig. 13c–g).

To further characterize the mechanism of the combined effect of ipat and PIMi, we performed IHC analysis on the apoptosis marker cleaved caspase 3 and the cell cycle marker cyclin D1 in the G-R3 X1.2 (Supplementary Fig. 12d) and the R0068 X1.2 (Supplementary Fig. 13h) tumors. Tumors treated with both ipat and GDC-0339 exhibited elevated percentages of tumor cells stained positive for cleaved caspase 3. Conversely, reduced levels of cyclin D1 were observed in the combination groups compared with vehicle controls or single agent groups, either quantified by the percentage of cells with strong and moderate cyclin D1 signals, or by digital histoscores, most significantly in the R0068 X1.2 tumors (Supplementary Figs. 12d and 13h). These data are consistent with both an increase in apoptosis and a reduction in cell cycle progression in tumors treated with the combination of ipatasertib and GDC-0339.

Taken together, these data demonstrate that PIM signaling mediates acquired resistance to the ATP-competitive AKTi ipatasertib, derived both in vitro in cell culture and in vivo as xenograft tumors, suggesting that PIM inhibitors may be potential candidates as combination partners for ATP-competitive AKT inhibitors in the clinic.

## Discussion

Given the high frequency of alterations in the PI3K/AKT signaling pathway in cancer and the well-established role of AKT signaling in mediating tumor survival and progression, inhibition of this pathway represents an attractive therapeutic approach in oncology^1^. Although clinical development of agents targeting different nodes of the pathway has been challenging due to limited efficacy and tolerability in solid tumors^40^, emerging clinical data suggest that direct targeting of AKT, the central node of the pathway, with AKTis such as ipatasertib can achieve favorable therapeutic index. Encouraging early evidence of efficacy has been observed in Phase II studies in mCRPC with ipatasertib in combination with abiraterone and in mTNBC with ipatasertib in combination with paclitaxel^13,41^. More recently, in the phase III IPATential150 trial, ipatasertib achieved significantly superior radiographic progression–free survival and antitumor activity in combination with abiraterone as first-line treatment for mCRPC in patients with PTEN loss by IHC^42^, further demonstrating the clinical relevance for AKT inhibitors in PTEN-null prostate cancers. Nevertheless, despite a 22% improvement in the objective response rate, the median rPFS improvement is only 2 months between the ipatasertib arm and the placebo arm, suggesting a short duration of the response to ipatasertib may limit the overall benefit an AKT inhibitor can potentially provide.

With two distinct classes of AKTis being investigated, we set out to understand whether overlapping or distinct mechanisms of acquired resistance would arise to different inhibitors. We took a systematic approach, using RNA-seq, exome-seq, and SNP array to identify genetic and non-genetic alterations that emerge following long-term exposure of the PTEN-deficient prostate cancer cell line, LNCaP, to allosteric or ATP-competitive AKT inhibitors. In addition, we exploited a chemical genetics screen to identify druggable pathways that mediate AKTi resistance, which provided us with candidate combination strategies to overcome acquired resistance. We discovered that distinct mechanisms drive acquired resistance to allosteric vs. ATP-competitive AKT inhibitors, which are consistent with their mechanisms of action, and are associated with differences in degree of cross-resistance to AKTis, sensitivity of AKT substrate phosphorylation to AKT inhibition and reversibility of resistance.

Allosteric inhibitors bind to the inactive conformation of AKT through stabilizing the PH-kinase domain interaction, therefore are susceptible to alterations in AKT isoforms themselves. Unlike ipatasertib, which is similarly potent against all three AKT isoforms^8^, allosteric inhibitors such as MK-2206 are reported to be less potent against AKT3 than AKT1 or AKT2^18,43,44^. Indeed, we found that AKT3 is upregulated in the M-R cells. Results from AKT3 knockdown and overexpression studies suggest that AKT3 upregulation plays a significant, albeit small role in MK-2206 resistance in the M-R lines. More importantly, we found the resistance of LNCaP M-R cells to be primarily driven by a W80C mutation in AKT1 identified by exome-seq. This finding is consistent with previous reports that W80 is critical for allosteric inhibitor binding and locking AKT in the inactive conformation^5,6,19–21^. Further, our results demonstrate that a W80R mutation identified in patient tumors can also confer resistance to allosteric but not ATP-competitive inhibitors. Interestingly, while no AKT2 alterations were detected in AKTi-resistant cells in our study, a W80C mutation in AKT2 was identified in human tumors and shown to confer resistance to MK-2206 when ectopically overexpressed in MCF-10A cells^20^.

In contrast to M-R cells, ipatasertib-resistant G-R cells did not harbor any alterations in AKT isoforms. Instead, they displayed resistance to both classes of AKTis, partial reversion of resistance following inhibitor withdrawal, and complete to partial responsiveness of AKT substrate phosphorylation to ipatasertib. However, the phosphorylation of targets downstream of mTORC1 was refractory to ipatasertib treatment in these cells. Importantly, the G-R cells were established at 5 μM of ipatasertib, a concentration that’s within the range of Cmax of clinically relevant ipatasertib exposure in patients^41^. Interestingly, although gatekeeper mutations have been shown to mediate resistance to ATP pocket binding drugs in other kinases^45^, similar mutations were not discovered in the G-R cells. This may be due to the fact that the AKT kinases have a relatively large methionine residue as a gatekeeper. Structural analysis suggests that ipatasertib fits snugly inside the ATP pocket, leaving few options for an even larger residue to substitute Met and sterically prevent ipatasertib binding without affecting ATP binding^46^.

Genetic instability of cancer cells will inevitably result in overwhelming numbers of changes in post-treatment tumor samples, making it difficult to distinguish driver alterations from passenger changes, which is further complicated by the fact that multiple alterations are often responsible for the resistance phenotype. Using a chemical genetics screen, we identified PIM signaling as an important mechanism of AKTi resistance in G-R cells, which would have been difficult to pinpoint merely from the exome-seq and RNA-seq results. Genetic validation using siRNA knockdown confirmed that depletion of PIM3 significantly increased sensitivity of G-R cells to ipatasertib, with the caveat of the incomplete nature of the siRNA effect. Considering the intrinsic genetic instability of the LNCaP cell line which is defective in mismatch repair genes^47,48^, CRISPR knockout experiments were not performed in these cells. Instead, we carried out Dox-inducible overexpression of each PIM isoform in LNCaP parental cells to recapitulate the reduced sensitivity of AKT downstream markers and cell viability to ipatasertib inhibition observed in G-R cells.

The PIM kinases share multiple substrates with both AKT and mTORC1^26–29^. Indeed, combined PIM and AKT inhibition more effectively suppresses phosphorylation of targets downstream of AKT/mTORC1 in G-R cells than either treatment alone. The importance of PIM signaling in acquired resistance to ipatasertib was not only evident in G-R cells in cell culture, but also validated in xenograft tumors established from these cells. Moreover, using xenograft tumors grown from the LNCaP parental cells, we independently derived ipatasertib-resistant tumors entirely through in vivo selection in immune-compromised male mice, and demonstrated similar dependence on PIM signaling in these models.

Increased expression of PIM kinases has been detected both in hematopoietic malignancies and in solid tumors such as prostate cancer, where their oncogenic potential is perhaps best characterized among solid tumors^49^. Elevated levels of all 3 PIM proteins have been observed in prostate cancer samples compared to benign patient samples^50^. Evidence from the literature supports the notion that PIM signaling may play an important role in AKTi resistance not only in prostate cancer but in a variety of indications. Indeed, PIM signaling has been implicated in intrinsic resistance to PI3K/AKT pathway inhibition, though the exact mechanism of PIM-mediated resistance and particular PIM isoform(s) mediating resistance appear to be context-dependent. For example, in PC3-LN4 prostate cancer cells, intrinsic resistance to both allosteric and ATP-competitive AKTis has been demonstrated to involve AKTi-induced PIM1 upregulation followed by a PIM1-dependent increase in receptor tyrosine kinase expression via cap-independent translation^51^. In various breast cancer cell lines, intrinsic resistance to the PI3Kα or AKT inhibitors has been demonstrated to involve PIM1, with PIM3 likely playing a less prominent role in this setting^52^. PIM2 expression has been linked to the resistance of breast cancer and MM cell lines to PI3K inhibitors GDC-0941 or BKM120^26,53^. Interestingly, PIM3 upregulation has been reported as a feedback mechanism in response to mTORC1 inhibition by rapamycin through miR-33 mediated suppression encoded by the SREBP loci^54^. In the current study, we did not observe increased PIM3 transcripts in the LNCaP parental or G-R cells in the presence of ipatasertib (Supplementary Fig. 10c). While most of the previous reports suggest that PIM signaling mediates intrinsic resistance to PI3K/AKT inhibition, our understanding of the role of PIM signaling in maintaining AKTi resistance following long term treatment is still unfolding. This study provides, for the first time, compelling evidence that PIM signaling plays a critical role in the acquired resistance to ATP-competitive inhibitors in a PTEN-deficient prostate cancer model. Importantly, our data suggest that cancer cells can rewire their signaling pathways from a predominantly strong dependence on AKT signaling to co-dependence on both AKT and PIM signaling. It’s recently reported that the expression of both PIM1 and PIM2 are further increased in CRPC compared to primary prostate cancer^50^. It is conceivable that in PTEN-null prostate cancers with lower levels of PIM expression, such as in early stage CRPC or hormone sensitive disease represented by the LNCaP cells, PIM upregulation can occur as an acquired or adaptive resistance after initial sensitivity to AKT inhibition, while in more advanced tumors where further increase in PIM expression occurs, intrinsic resistance to AKT inhibitors may be observed.

Overall, we have demonstrated that distinct mechanisms of acquired resistance arise to allosteric vs. ATP-competitive AKT inhibition. Further, we have uncovered resistance mechanisms in both the allosteric and ATP-competitive AKTi-resistant settings that are clinically actionable. Separate mechanisms of resistance were identified using multiple systematic approaches, including RNA-seq, exome-seq, and a chemical library screen, highlighting the utility of combining approaches in the search for resistance drivers. Alterations in AKT isoforms, including the first report of the acquisition of a mutation in *AKT1* at a residue found to be altered in human patients, were associated with acquired resistance to allosteric AKTi yet responsiveness to ATP-competitive AKTi. Therefore, ipatasertib treatment may represent a potential therapeutic strategy for patients with acquired resistance to allosteric AKT inhibition. Alternatively, PIM signaling was found to play an important role in acquired resistance to ATP-competitive AKT inhibition, both in the in vitro and in vivo settings. Hence, acquired resistance to ATP-competitive AKT inhibition in the clinic may be reversed by combined treatment with a PIM inhibitor.

## Methods

### Compounds and antibodies

Compounds were supplied by in-house synthesis at Genentech, Inc. or purchased from vendors. Antibodies used from immunoblots to AKT (#2920, 1:1000), AKT1 (#2938, 1:1000), AKT2 (#3063, 1:1000), AKT3 (#8018, 1:1000), pAKT(T308) (#2965, 1:1000), pAKT(S473) (#9271, 1:1000), pPRAS40(T246) (#2997, 1:1000), PRAS40 (#2691, 1:1000), pS6(S235/236) (#2211, 1:1000), S6 (#2317, 1:1000), p4EBP1 (T37/46) (#2855, 1:1000), p4EBP1 (S65) (#9456 1:1000), 4EBP1 (#9452, 1:1000), PARP (#9532 1:1000), Cleaved PARP (#5625, 1:1000), PTEN (#9556, 1:1000 and #9559, 1:1000), PIM2 (#4730, 1:500), PIM3 (#4165, 1:500), pGSK-3β (S9) (#9336, 1:500), GSK-3β (#9832, 1:500), pBAD (S112) (#9239, 1:500), and BAD (#9239, 1:500) were obtained from Cell Signaling Technology. The PIM1 antibody was obtained from Abnova (H00005292-M01, 1:500). An additional antibody to total PRAS40 was obtained from Invitrogen/ThermoFisher (AHO1031, 1:1000). Protein loading was assessed using antibodies to β-actin (Sigma-Aldrich, A5441, 1:3000), β-Tubulin (Sigma-Aldrich, T8328, 1:5000) or glyceraldehyde-3-phosphate dehydrogenase (GAPDH) (Advanced ImmunoChemical, 2-RGM2, 1:2000).

### Contact for reagent and resource sharing

Further information and requests for reagents may be directed to, and will be fulfilled by, the corresponding author, Kui Lin (lin.kui@gene.com).

### Cell lines and cell culture

Cell lines were originally obtained from the American Type Culture Collection (ATCC) and genotyped by Genentech’s cell banking facility. LNCaP is an approximately tetraploid epithelial line derived from a prostate adenocarcinoma metastasis^55,56^. LNCaP cells harbor a frameshift mutation (K6fs*4) (COSMIC # COSM4929) and loss of heterozygosity (LOH) in PTEN^57,58^. The presence of both alterations was confirmed in the LNCaP line in-house via exome sequencing and SNP array. AKTi-resistant (AKTi-R) cell lines were established by treating cells with gradually escalating doses of ipatasertib or MK-2206 until reaching a maximum dose of 5 μM of AKTi. ipatasertib-resistant (G-R) and MK-2206-resistant (M-R) cell pools were then subjected to single cell sorting using FACSAria instrumentation and software (BD Biosciences) and surviving clones were expanded in the presence of AKTi at the maximum doses indicated above. MK-2206 or ipatasertib-resistant cell pools are denoted as M-Rpool or G-Rpool, respectively. Individual AKTi-R clones were assigned numbers, which are indicated following the M-R or G-R prefix. LNCaP G-RB cell lines were established following long-term exposure of LNCaP cells to 5 μM ipatasertib, single cell sorting, and expansion of surviving clones in the presence of the AKTi. The Par X1.6 and G-R3 X1.2 selected lines used for in vivo studies were derived from LNCaP and LNCaP G-R clone 3 (G-R3) tumors that displayed growth in untreated male SCID.bg C.B-17 mice (Charles River Labs). The R0068 X1.2 line was established from mice bearing LNCaP tumors that had been treated with 50 mg/kg ipatasertib over the course of 106 days. Whole tumors from individual mice were excised, minced in complete media, and plated in tissue culture flasks. Cell lines were established following one to two passages. After establishment in culture, G-R3X1.2 and R0068 X1.2 cells were maintained in the presence of 5 μM ipatasertib. All cell lines were maintained at 37 °C/5% CO_2_ in Roswell Park Memorial Institute medium (RPMI) 1640 supplemented with 10% fetal bovine serum (FBS) (Sigma), 2 mM L-Glutamine, and 0.01 M HEPES, pH 7.2. Growth medium for AKTi-R cell lines was additionally supplemented with AKTi at the indicated concentration for cell line maintenance.

### In Vivo Efficacy Studies

All in vivo efficacy studies were approved by Genentech’s Institutional Animal Care and Use Committee and adhere to the National Institutes of Health Guidelines for the Care and Use of Laboratory Animals. Tumor xenografts derived from the LNCaP Par and LNCaP G-R3 X1.2 cell lines were established by subcutaneous injection of 10 × 10^6^ cells into male NOD *scid* gamma (NSG) mice (Jackson Laboratories, Sacramento, CA). Testosterone pellets (12.5 mg/pellet, 60-day release, no. SA-151; Innovative Research of America) were implanted into the dorsal shoulder 5 days prior to cell inoculation. Animals were distributed into treatment groups (*n* = 9/group) when the tumors reached a mean volume of approximately 230 to 350 mm^3^. GDC-0068 and GDC-0339 were formulated in 0.5% methylcellulose/0.2% Tween-80 (MCT) and were administered once daily (QD) via oral formulation (*per os*; PO) at 25 and 100 mg/kg, respectively for 21 days. Tumor volumes were determined using digital calipers (Fred V. Fowler Company, Inc, Newton MA) using the formula (*L x W x W*)/2. Tumor volumes and body weights were recorded twice weekly over the course of the study. Mice with tumor volumes >2000 mm^3^ or recorded body weight loss of >20% from their start of treatment were euthanized per Institutional Animal Care and Use Committee guidelines.

Analysis and comparison of tumor growth was performed as detailed previously using a package of customized functions in R v3.6.2 (R Development Core Team 2008; R Foundation for Statistical Computing, Vienna, Austria) which integrate software from open source packages including lme4, mgcv, gamm4, multcomp, settings, plyr, and several packages from the tidyverse such as magrittr, dplyr, tidyr, and ggplot2^8,59^. In brief, as tumors generally exhibit exponential growth, tumor volumes were subjected to natural log transformation before analysis. A generalized additive mixed model (GAMM) was then applied to describe the change of transformed tumor volumes over time using regression splines with auto-generated spline bases as this approach addresses both repeated measurements from the same study subjects and moderate dropouts before study end. Estimates of group-level efficacy were obtained by calculating a growth contrast, the difference in AUC-based growth rates (i.e., eGaIT) between the treatment and reference group fits. To calculate AUC-based growth rates, group AUC values are corrected for starting tumor burden and then subjected to slope equivalence “normalization”. Slope equivalence “normalization” of AUC results in the actual slope of a fit on the natural log (LN) scale in cases of log-linear growth. In the cases of non-log-linear growth, such “normalization” results in the constant log-linear growth rate that would have been needed to yield the baseline-corrected AUC that was actually observed for a fit on the natural log scale. Mathematically, this “normalization” is attained by dividing each estimated baseline-corrected AUC value by half of the square of the common study period resulting in units of natural log units per day. The more negative the Growth Contrast value, the greater the anti-tumor effect. The 95% confidence intervals are based on the fitted model and variability measures of the data.

### Analysis of body weights

A generalized additive mixed model (GAMM) was also employed to describe the change in raw body weights (i.e., grams) over time with regression splines. After data fitting, raw body weight data at each time point from all individual animals and all group fits were normalized to the starting weight and reported as a percentage to yield % body weight change.

### Viability assay

Cellular viability was assessed 4 days after addition of inhibitors using the CellTiter-Glo® (Promega) luminescent assay as described previously^8^. Briefly, cells were plated in black/clear bottom 384 well plates (BD Falcon) and incubated at 37 °C under 5% CO_2_. The following day, cells were treated with a 9-point dose titration of indicated inhibitors or with DMSO control. All conditions were tested in quadruplicate within each experiment. Treated cells were then incubated for 4 days and viability was assessed using the CellTiter-Glo® (Promega) luminescent assay according to the manufacturer’s instructions. Total luminescence was measured on a Wallac Multilabel Reader (PerkinElmer) and was considered to represent cellular viability. Dose response curves (nonlinear fit, 4-parameter) generated with Prism (GraphPad) depict mean % viability (% DMSO control), with error bars representing standard error of the mean (SEM), from quadruplicate samples (y-axis) versus concentration of inhibitor (x-axis) from a single representative experiment. The inhibitor concentration resulting in the half maximum inhibitor effect (IC_50_) was calculated from % viability values from quadruplicate wells using a 4-parameter curve analysis (XLfit, IDBS software). Scatter plots depict absolute IC_50_ values from independent biological repeats, with bars denoting mean absolute IC_50_ values and standard error of the mean (SEM). Mean IC_50_ values are denoted below x-axis and sample size (*n*) is indicated for each cell line/condition above scatter plot data. Unless otherwise stated, at least 3 independent experiments were performed to assess reproducibility. Where applicable, statistical significance is indicated above scatter plot data used to make comparisons.

### Immunoblotting

Cells were washed with cold 1X phosphate-buffered saline (PBS) and lysed in Cell Extraction Buffer (CEB) (Biosource/Thermo Fisher Scientific) supplemented with a protease inhibitor cocktail (Sigma) and phosphatase inhibitor cocktail (Roche). Protein concentrations were determined using the Lowry-based RC DC protein assay (Bio-Rad) and normalized for equal protein loading. Lysates were loaded onto Tris–glycine gels (Invitrogen) and proteins were separated by electrophoresis. Proteins were then transferred onto nitrocellulose membranes using the iBlot® dry blotting system (Invitrogen), and membranes were blocked with blocking buffer for fluorescent Western blotting (Rockland or LI-COR). Primary antibodies (see below) were detected using IR Dye 800-conjugated (Rockland or LI-COR) or Alexa Fluor 680 (Invitrogen) or IR Dye 680 (LI-COR) species-selective secondary antibodies. Detection and quantification were conducted using an Odyssey infrared scanner (LI-COR) using the manufacturer’s software. Protein loading was assessed using antibodies to β-actin, β-Tubulin, or GAPDH. Raw immunoblot images for cropped blots are provided in the Supplementary Information.

### RNA-seq and gene expression analysis

Par or AKTi-R cells were plated in duplicate in complete RPMI medium and, the following day, treated with DMSO, 5 μM ipatasertib, or MK-2206 for 14 h. Cells were then subjected to total RNA extraction using the RNeasy kit (Qiagen) and RNA concentrations were read using a NanoDrop 8000 spectrophotometer (Thermo Scientific). Following confirmation of RNA integrity with the 2200 TapeStation system (Agilent Technologies), RNA-seq libraries were prepared using the TruSeq RNA Sample Preparation Kit v2 (Illumina) from 1 μg of total RNA. Library size was determined using 2200 TapeStation and High Sensitivity D1000 screen tape (Agilent Technologies) and concentration was assessed by qPCR-based methodology (Library quantification kit, KAPA). The libraries were multiplexed and sequenced on Illumina HiSeq2500 (Illumina) to generate 50 million paired-end 75 base pair reads. RNA-seq reads containing 30% or more bases with a Phred quality score of 23 or lower were excluded. The remaining high quality reads were then mapped to NCBI GRCh37 (hg19) using GSNAP and default settings. Multimapping reads were discarded. Gene expression levels were summarized into count and RPKM (reads per kilobase of exon model per million mapped reads normalized by sample size factor). Differential expression analysis was performed using limma. Genes with adjusted p-value < 0.05 and absolute value of log2FC > = 1 were considered to be differentially expressed. For the hierarchical clustering and heatmap of RNA-seq transcriptome analysis, the RPKM values for the top 100 most variably expressed genes were z-scored and clustered using Euclidean distance.

### Exome-seq

Par or AKTi-R cells were plated in complete RPMI medium and the following day, DNA extraction was performed using the DNeasy kit (Qiagen). Prior to processing by whole exome sequencing, the concentration and integrity of DNA samples was determined using NanoDrop 8000 (Thermo Fisher Scientific) and 2200 TapeStation (Agilent Technologies), respectively. Exome capture was performed using 0.5 μg of genomic DNA and SureSelectXT Human All Exon v5 kit (50 megabases [Mb]) according to manufacturer’s protocol (Agilent Technologies, CA). Fragment size distribution of post-capture amplified libraries was determined with 2200 TapeStation using high sensitivity D1000 screen tape (Agilent Technologies, CA). Concentration of the libraries was measured by Qubit (Thermo Fisher Scientific). Exome capture libraries were sequenced on HiSeq 2500 (Illumina, CA) to generate 75 million paired-end 75 base pair reads. High quality exome-seq reads were mapped to NCBI GRCh38 using GSNAP. Somatic SNVs and INDELs were called by comparing the treatment resistant clones against the parental clones using LoFreq with its default setting. Highly-confident variants were annotated using Ensembl Variant Effect Predictor and filtered with dbSNP 138, ExAC 0.3.1 and RepeatMasker 4.0.5. The functional consequences of somatic variants were annotated using SIFT, PolyPhen and Condel.

### SNP array

DNA from parental or AKTi-R cells was extracted and assessed for quality and quantity as described above. Illumina HumanOnmi2.5-8 arrays were then used to assay genotype, DNA copy number, and loss of heterozygosity as described previously^60^.

### siRNA

The Lipofectamine RNAi Max (Life Technologies) transfection reagent was used to transfect cells with siRNA oligonucleotides (see table below) according to manufacturer’s instructions. Briefly, siRNA oligonucleotides and Lipofectamine RNAi Max were each mixed with Opti-MEM® (Invitrogen) in separate microcentrifuge tubes or wells in multiwell plates. These mixtures were combined and incubated for 15 min prior to mixing with cells in suspension. Cells were transfected with a final concentration of 25 nM siRNA duplexes. ON-TARGETplus SMART pool and individual siRNA oligonucleotides are reported to be highly specific, as verified by microarray analysis, as a result of unique dual-strand modification patterns used to synthesize the reagents.

**Table:** 

siRNA target	Sequence	Supplier	Description	Order #
NTC (control)	UGGUUUACAUGUCGACUAA	Dharmacon	ON-TARGETplus SMART pool	D-001810-10
	UGGUUUACAUGUUGUGUGA			
	UGGUUUACAUGUUUUCUGA			
	UGGUUUACAUGUUUUCCUA			
AKT1	CAUCACACCACCUGACCAA	Dharmacon	ON-TARGETplus	J-003000-10
AKT3	GCACACACUCUAACUGAAA	Dharmacon	ON-TARGETplus SMART pool	J-003002-00
	GAAGAGGGGAGAAUAUAUA			
	GUACCGUGAUCUCAAGUUG			
	GACAGAUGGCUCAUUCAUA			
PIM3	GGCCGUCGCUGGAUCAGAU	Dharmacon	ON-TARGETplus SMART pool	L-032287-00
	GCAGGACCUCUUCGACUUU			
	GCGUGCUUCUCUACGAUAU			
	GGACGAAAAUCUGCUUGUG			

### AKT3 overexpression

LNCaP cells were transfected with a pCMV6-AC-GFP vector containing the human AKT3 sequence (NM_005465) (Origene) or with EV using Fugene HD FuGENE® HD (Promega Corporation). 48 h later, cells were subjected to selection with 0.4 mg/l G418/Geneticin (Invitrogen/Gibco) and surviving cells were expanded. Cells were then sorted FACSAria instrumentation and associated software (BD Biosciences) for positive GFP expression and were maintained in the presence of 0.4 mg/l G418/Geneticin.

### AKT1 WT vs. W80C overexpression

The piggyBac transposon-based system (System Biosciences) was used to introduce a cumate-inducible version of AKT1 WT or W80C to LNCaP Par or M-R7 cells. Using NheI and BstBI, the following sequences were cloned into the B-Cuo-MCS-IRES-GFP-EF1-CymR-Puro Inducible cDNA Cloning and Expression Vector (System Biosciences #PBQM812A-1):

AKT1 WT

GGCGCCACCATGAGCGACGTGGCTATTGTGAAGGAGGGTTGGCTGCACAAACGAGGGGAGTACATCAAGACCTGGCGGCCACGCTACTTCCTCCTCAAGAATGATGGCACCTTCATTGGCTACAAGGAGCGGCCGCAGGATGTGGACCAACGTGAGGCTCCCCTCAACAACTTCTCTGTGGCGCAGTGCCAGCTGATGAAGACGGAGCGGCCCCGGCCCAACACCTTCATCATCCGCTGCCTGCAGTGGACCACTGTCATCGAACGCACCTTCCATGTGGAGACTCCTGAGGAGCGGGAGGAGTGGACAACCGCCATCCAGACTGTGGCTGACGGCCTCAAGAAGCAGGAGGAGGAGGAGATGGACTTCCGGTCGGGCTCACCCAGTGACAACTCAGGGGCTGAAGAGATGGAGGTGTCCCTGGCCAAGCCCAAGCACCGCGTGACCATGAACGAGTTTGAGTACCTGAAGCTGCTGGGCAAGGGCACTTTCGGCAAGGTGATCCTGGTGAAGGAGAAGGCCACAGGCCGCTACTACGCCATGAAGATCCTCAAGAAGGAAGTCATCGTGGCCAAGGACGAGGTGGCCCACACACTCACCGAGAACCGCGTCCTGCAGAACTCCAGGCACCCCTTCCTCACAGCCCTGAAGTACTCTTTCCAGACCCACGACCGCCTCTGCTTTGTCATGGAGTACGCCAACGGGGGCGAGCTGTTCTTCCACCTGTCCCGGGAGCGTGTGTTCTCCGAGGACCGGGCCCGCTTCTATGGCGCTGAGATTGTGTCAGCCCTGGACTACCTGCACTCGGAGAAGAACGTGGTGTACCGGGACCTCAAGCTGGAGAACCTCATGCTGGACAAGGACGGGCACATTAAGATCACAGACTTCGGGCTGTGCAAGGAGGGGATCAAGGACGGTGCCACCATGAAGACCTTTTGCGGCACACCTGAGTACCTGGCCCCCGAGGTGCTGGAGGACAATGACTACGGCCGTGCAGTGGACTGGTGGGGGCTGGGCGTGGTCATGTACGAGATGATGTGCGGTCGCCTGCCCTTCTACAACCAGGACCATGAGAAGCTTTTTGAGCTCATCCTCATGGAGGAGATCCGCTTCCCGCGCACGCTTGGTCCCGAGGCCAAGTCCTTGCTTTCAGGGCTGCTCAAGAAGGACCCCAAGCAGAGGCTTGGCGGGGGCTCCGAGGACGCCAAGGAGATCATGCAGCATCGCTTCTTTGCCGGTATCGTGTGGCAGCACGTGTACGAGAAGAAGCTCAGCCCACCCTTCAAGCCCCAGGTCACGTCGGAGACTGACACCAGGTATTTTGATGAGGAGTTCACGGCCCAGATGATCACCATCACACCGCCTGACCAAGATGACAGCATGGAGTGTGTGGACAGCGAGCGCAGGCCCCACTTCCCCCAGTTCTCCTACTCGGCCAGCGGCACGGCCTGA

AKT1 W80C

GGCGCCACCATGAGCGACGTGGCTATTGTGAAGGAGGGTTGGCTGCACAAACGAGGGGAGTACATCAAGACCTGGCGGCCACGCTACTTCCTCCTCAAGAATGATGGCACCTTCATTGGCTACAAGGAGCGGCCGCAGGATGTGGACCAACGTGAGGCTCCCCTCAACAACTTCTCTGTGGCGCAGTGCCAGCTGATGAAGACGGAGCGGCCCCGGCCCAACACCTTCATCATCCGCTGCCTGCAGTGTACCACTGTCATCGAACGCACCTTCCATGTGGAGACTCCTGAGGAGCGGGAGGAGTGGACAACCGCCATCCAGACTGTGGCTGACGGCCTCAAGAAGCAGGAGGAGGAGGAGATGGACTTCCGGTCGGGCTCACCCAGTGACAACTCAGGGGCTGAAGAGATGGAGGTGTCCCTGGCCAAGCCCAAGCACCGCGTGACCATGAACGAGTTTGAGTACCTGAAGCTGCTGGGCAAGGGCACTTTCGGCAAGGTGATCCTGGTGAAGGAGAAGGCCACAGGCCGCTACTACGCCATGAAGATCCTCAAGAAGGAAGTCATCGTGGCCAAGGACGAGGTGGCCCACACACTCACCGAGAACCGCGTCCTGCAGAACTCCAGGCACCCCTTCCTCACAGCCCTGAAGTACTCTTTCCAGACCCACGACCGCCTCTGCTTTGTCATGGAGTACGCCAACGGGGGCGAGCTGTTCTTCCACCTGTCCCGGGAGCGTGTGTTCTCCGAGGACCGGGCCCGCTTCTATGGCGCTGAGATTGTGTCAGCCCTGGACTACCTGCACTCGGAGAAGAACGTGGTGTACCGGGACCTCAAGCTGGAGAACCTCATGCTGGACAAGGACGGGCACATTAAGATCACAGACTTCGGGCTGTGCAAGGAGGGGATCAAGGACGGTGCCACCATGAAGACCTTTTGCGGCACACCTGAGTACCTGGCCCCCGAGGTGCTGGAGGACAATGACTACGGCCGTGCAGTGGACTGGTGGGGGCTGGGCGTGGTCATGTACGAGATGATGTGCGGTCGCCTGCCCTTCTACAACCAGGACCATGAGAAGCTTTTTGAGCTCATCCTCATGGAGGAGATCCGCTTCCCGCGCACGCTTGGTCCCGAGGCCAAGTCCTTGCTTTCAGGGCTGCTCAAGAAGGACCCCAAGCAGAGGCTTGGCGGGGGCTCCGAGGACGCCAAGGAGATCATGCAGCATCGCTTCTTTGCCGGTATCGTGTGGCAGCACGTGTACGAGAAGAAGCTCAGCCCACCCTTCAAGCCCCAGGTCACGTCGGAGACTGACACCAGGTATTTTGATGAGGAGTTCACGGCCCAGATGATCACCATCACACCGCCTGACCAAGATGACAGCATGGAGTGTGTGGACAGCGAGCGCAGGCCCCACTTCCCCCAGTTCTCCTACTCGGCCAGCGGCACGGCCTGA

These sequences include a silent mutation to confer resistance to the ON-TARGET plus siRNA oligonucleotide (Dharmacon) targeting AKT1 with the sequence CAUCACACCACCUGACCAA. The piggyBac expression vector includes a GFP expression cassette separated from the AKT1 sequence by an internal ribosome entry site (IRES), enabling independent expression of both genes from a single transcript. Using either the PureFection™ (System Biosciences) or FuGENE® HD (Promega Corporation) transfection reagents, LNCaP Par or M-R7 cells were transfected with the piggyBac transposase (System Biosciences) combined with the empty piggyBac expression vector, AKT1 WT piggyBac vector, or AKT1 W80C piggyBac vector. Transfected cells were then incubated for 48 h prior to selection with 1 μg/ml puromycin and subsequently expanded. Leaky expression was minimized by using FACSAria instrumentation and associated software (BD Biosciences) to select for cells that do not express GFP in the absence of cumate. In an effort to obtain populations that express similar levels of AKT1 WT vs. AKT1 W80C, cells were treated with 10 μg/ml cumate for 5 days (conditions previously confirmed to induce expression in all relevant cell lines) and were subjected to sorting for specific GFP expression levels using FACSAria instrumentation and associated software (BD Biosciences).

### Cell sorting

Cell sorting was performed on multiple FACSAria™ Fusions running DIVASoftware v8.0.1 equipped with 5 lasers (355 nm, 405 nm, 488 nm, 561 nm, 638 nm) (BD Biosciences). The instruments were set up with a nozzle size of 100 micron at a frequency of 32 kHz and pressure of 20 psi. The “Four-Way Purity” sort mode was used for coincident discrimination.

### Assessment of prevalence of AKT1 W80 alterations in human cancer indications

Cancer genomics studies in which AKT1 W80 alterations were detected in patients were first identified using cBioPortal (http://www.cbioportal.org/index.do?session_id=5b5e1288498eb8b3d5672636). All AKT1 mutation information reported in those selected studies was then retrieved. The frequency of each AKT1 mutation detected within the same indication was calculated from these studies (# of patients harboring a specific AKT1 mutation/total # patients with that indication within the data set). Data bases used include TCGA: The Cancer Genome Atlas, https://portal.gdc.cancer.gov; METABRIC: Molecular Taxonomy of Breast Cancer International Consortium (Nature 2012 & Nat Commun 2016), Pierra et al., 2016 https://www.ncbi.nlm.nih.gov/pubmed/27161491; MSK-IMPACT: Memorial Sloan Kettering Cancer Center’s Integrated Mutation Profiling of Actionable Cancer Targets (MSKCC, Nat Med 2017).

### Analysis of the impact of WT vs mutant AKT1 in Ba/F3 cells

The impact of WT or mutant AKT1 on sensitivity to MK-2206 or ipatasertib was assessed using the IL-3 independent viability assay in the Ba/F3 model. Survival of the Ba/F3 murine pro-B cell line is constitutively growth-factor dependent but can be rendered IL-3 independent via co-expression of AKT1 and an activated form of the MAP2 kinase mitogen-activated protein kinase (MAPK)/extracellular-signal- regulated kinase (ERK) kinase (MEK1) (Mek1 ΔN3, S218E, S222D), termed MEK1 N3^22^. As described previously^22^, N-terminally FLAG-tagged AKT1 (WT) was constructed using standard PCR techniques and mutants were generated using the QuikChange Site-Directed Mutagenesis Kit (Stratagene/Agilent Technologies). Mutant or WT AKT1 was cloned into the pRetro-internal ribosome entry site (IRES)-GFP vector (Clontech). MEK1 N3 was constructed as previously described^61^ and cloned into the pMXs-puro retroviral vector (Cell Biolabs). Retroviral constructs expressing the WT or mutant protein were transfected into the Phoenix amphoteric packaging cell line using Fugene6 (Roche). Viral supernatant was harvested 48 h after transfection and filtered using a 0.45-μM syringe filter. Ba/F3 cells were then infected with virus by spinoculation (1,800 revolutions per minute [RPM] for 45 min), and cells infected with WT or mutant AKT1 were sorted by flow cytometry based on GFP fluorescence. Infected cells were selected with 2 μg/mL puromycin for 7 days. Pools of these cells were used for subsequent studies. All Ba/F3-derived cell lines were maintained in culture in the presence of 2 ng/mL recombinant murine IL-3 (R&D Systems). For analysis of the impact of WT vs. mutant AKT1 on the response to AKT inhibition, Ba/F3 cells which stably co-express MEK1 N3 and WT or mutant AKT1 were washed three times with 1X PBS and plated in the absence of IL-3 in complete RPMI in 384 well plates (1000 cells per well). The next day, cells were treated with a 9-point dose-titration of MK-2206 or ipatasertib using a maximum dose of 20 μM. DMSO controls were included and all conditions were tested in 4 replicate wells. Cellular viability was assessed 4 days after addition of inhibitors using the CellTiter-Glo® (Promega) luminescent assay as described previously^8^. For analysis of protein levels, lysates were prepared from cells cultured in the absence of IL-3 for 1 day and subjected to Western blotting as described above.

### Chemical genetics screen

A library consisting of 426 compounds including targeted agents, chemotherapeutics, and tool compounds was used to screen for inhibitors associated with enhanced sensitivity in AKTi-R cells compared with Par cell lines. Compounds were obtained from in-house synthesis or purchased from commercial vendors. Cells were plated in 384 well plates (BD Falcon) using seeding densities previous determined to achieve approximately 70–80% confluence at the final time point of the assay. AKTi-R cells were plated in the presence of inhibitor at the dose used to maintain the resistance and Par cells were plated in DMSO control media. The following day, cells were treated with a 9-point dose titration of each inhibitor or DMSO control and 4 days later, cell viability was assessed as described above. Screening drug management, quality control, and the calculation of drug response statistics were performed as described previously^62^. The data was processed using Genedata Screener, Version 14 (Genedata; Basel, Switzerland), with a four‑parameter Hill equation using compound dose-response data normalized to the median of 42 vehicle‑treated wells on each plate. A “Robust Fit” strategy was also employed by Genedata Screener, which is based on Tukey’s biweight and is resistant to outlier data. The reported absolute IC50 is the dose at which cross‑run estimated inhibition is 50% relative to DMSO control wells. In addition to absolute IC50, mean fitted viability across the nine tested doses (i.e., area under the viability curve) was also computed.

### Matrix combination experiments

Par or G-R cells were plated in 384 well plates and 24 h later were treated with a 9-point dose titration of GDC-0068/ipatasertib, a second inhibitor, or a combination of the two. DMSO controls were also included. After a further 4 days, cell viability was assessed using the CellTiter-Glo® assay as described above. The combination effect of ipatasertib and other inhibitors was assessed by Bliss independence analysis^63^ as well as Highest single agent (HSA) analysis^64^, as described previously^30,65^. Briefly, a Bliss expectation for a combined response (*C*) of drugs A and B was calculated by the equation: *C* = (*A* + *B*) − (*A* × *B*) where *A* and *B* are the fractional growth inhibitions of each respective drug at a given dose. The difference between the Bliss expectation and the observed growth inhibition of the combination of drugs A and B at the same dose is the “Delta.Bliss.” Delta.Bliss scores were summed across the dose matrix to generate a Bliss sum. Bliss sum = 0 indicates that the combination treatment is additive (as expected for independent pathway effects); Bliss sum > 0 indicates activity greater than additive (synergy); and Bliss sum <0 indicates the combination is less than additive (antagonism). Using the Highest single agent (HSA) model^64^, scores were calculated at each dose matrix point based on the excess loss of viability in the combination in comparison to the highest single drug response. At least three independent experiments were performed to assess reproducibility unless otherwise indicated. Heatmaps depict % viability inhibition, Bliss score, or HSA score associated with each dose combination point following treatment of cells with the two agents. Mean Bliss sum values from three independent biological replicates are depicted in scatter plots, with mean Bliss sum values denoted below the x-axis and sample size (n) indicated for each cell line/condition above scatter plot data.

### Inducible PIM1, PIM2, and PIM3 overexpression

Sequences encoding the full length forms of human PIM1 (NP_001230115.1), PIM2 (NP_006866.2), PIM3 (NP_001001852.2), or a kinase-deficient PIM3 K69M mutant^66^ were cloned into the BH1.4 Dox-inducible piggyBac vector with an IRES-turboGFP-nuclear expression marker (Genentech, Inc.). LNCaP Par cells were transfected with the piggyBac transposase combined with the empty BH1.4 vector or the PIM expression constructs. Transfected cells were then subjected to selection with puromycin and subsequently expanded. Leaky expression was minimized by FACS sorting to select for cells that do not express GFP in the absence of Dox. In an effort to obtain populations that express similar levels of PIM proteins, cells were treated with 100 ng/ml Dox for 3 days and subjected to sorting for specific GFP expression levels.

### Analysis of xenograft tumors

Flash frozen tumors were pulverized to powder by mechanical force (Covaris cryoPREP). Tumor powder was lysed with RIPA lysis buffer (Sigma) supplemented with a cocktail of phosphatase and protease inhibitors (Thermo Fisher Scientific) and 1 mM PMSF (Sigma). Tumor lysates were homogenized by mechanical disruption (SPEX SamplePrep) and cleared by centrifugation at 14,000 rpm for 10 min at 4 °C. Protein levels were quantified by the BCA Protein Assay Kit (Pierce Biotechnology) and normalized to equal concentrations. Equal amounts of protein lysates were resolved using NuPAGE Bis-Tris precast gels (Thermo Fisher Scientific) and transferred onto nitrocellulose membranes using the iBlot® dry blotting system (Thermo Fisher Scientific). Membranes were blocked with blocking buffer for fluorescent Western blotting (LI-COR). Primary antibodies were detected using IR Dye 800-conjugated (LI-COR) or IR Dye 680-conjuated (LI-COR) species-selective secondary antibodies. Detection and quantification were conducted using an Odyssey infrared scanner (LI-COR) using the manufacturer’s software. Protein loading was assessed using antibodies to β-actin or GAPDH.

### Immunohistochemistry

Immunohistochemisty (IHC) for xenograft tumors were carried out using 5-μm paraffin sections of formalin-fixed tissue on a VentanaBenchmark XT instrument (VMSI) by deparaffinization, treatment with antigen retrieval buffer (VMSI) and incubation with primary antibodies against PTEN (#9559), pAKT (S473) (#4060), pPRAS40 (T246) (#2997), pS6 (S235/236) (#2211), cleaved caspase 3 (Asp175) (#9661) (all from Cell Signaling Technology), or cyclin D1 (AbCam, ab16663) at 37 °C. Bound antibody was detected using DABMap technology (VMSI) and sections were counterstained with hematoxylin. IHC stained slides were scanned on a NanoZoomer XR whole slide imager (Hamamatsu, Bridgewater NJ) at 200x magnification, and were used to quantify the tumor and IHC positive staining areas. Segmentation of tumor regions and DAB positive pixels was performed by a custom algorithm using standard morphological operations and global RGB color thresholds running on Matlab 2019a (Mathworks, Natick, MA).

### Statistics and reproducibility

A two-tailed Student’s t-test or a one-way analysis of variance (ANOVA) was performed when comparing two groups or more than two groups, respectively. Statistical analysis was carried out using Prism v9.3.1 (GraphPad) and Microsoft Excel. Data are expressed as the mean ± standard deviation (SD) or standard error of means (SEM) as described in the figure legends. Each experiment was repeated at least three times unless otherwise indicated.

### Reporting summary

Further information on research design is available in the Nature Research Reporting Summary linked to this article.

## Supplementary information

Supplementary Information

Inventory of Supporting Information

Supplementary Dataset 1

Supplementary Dataset 2

Supplementary Dataset 3

Supplementary Dataset 4

Supplementary Dataset 5

Reporting Summary

## Data Availability

RNA-seq data generated in this study have been deposited in Gene Expression Omnibus (GEO) under accession number GSE139178, at. Whole-exome sequencing data generated in this study have been deposited in Sequence Read Archive (SRA) under accession number PRJNA578183, at. Cancer genomics studies in which AKT1 W80 alterations were detected in patients were first identified using cBioPortal (http://www.cbioportal.org/index.do?session_id=5b5e1288498eb8b3d5672636). All AKT1 mutation information reported in those selected studies was then retrieved. The frequency of each AKT1 mutation detected within the same indication was calculated from these studies (# of patients harboring a specific AKT1 mutation/total # patients with that indication within the data set). Data bases used include TCGA: The Cancer Genome Atlas, https://portal.gdc.cancer.gov; METABRIC: Molecular Taxonomy of Breast Cancer International Consortium (Nature 2012 & Nat Commun 2016), Pierra et al., 2016 https://www.ncbi.nlm.nih.gov/pubmed/27161491; MSK-IMPACT: Memorial Sloan Kettering Cancer Center’s Integrated Mutation Profiling of Actionable Cancer Targets (MSKCC, Nat Med 2017). Source data are provided with this paper.

## Source data

Source Data

## Code availability

Differential expression analysis of RNA-seq data was performed using limma. For the hierarchical clustering and heatmap of RNA-seq transcriptome analysis, the RPKM values for the top 100 most variably expressed genes were z-scored and clustered using Euclidean distance. Somatic SNVs and INDELs were called by comparing the treatment resistant clones against the parental clones using LoFreq with its default setting. Highly-confident variants were annotated using Ensembl Variant Effect Predictor and filtered with dbSNP 138, ExAC 0.3.1 and RepeatMasker 4.0.5. The functional consequences of somatic variants were annotated using SIFT, PolyPhen and Condel.
