# Supplementary material for: Distinct resistance mechanisms arise to allosteric vs. ATP-competitive AKT inhibitors
Source: Nat Commun. 2022 Apr 19;13:2057. doi: 10.1038/s41467-022-29655-0 (PMC9019088; doi:10.1038/s41467-022-29655-0)
Supplement: Supplementary file 1 — Supplementary Information [file 41467_2022_29655_MOESM1_ESM.pdf]

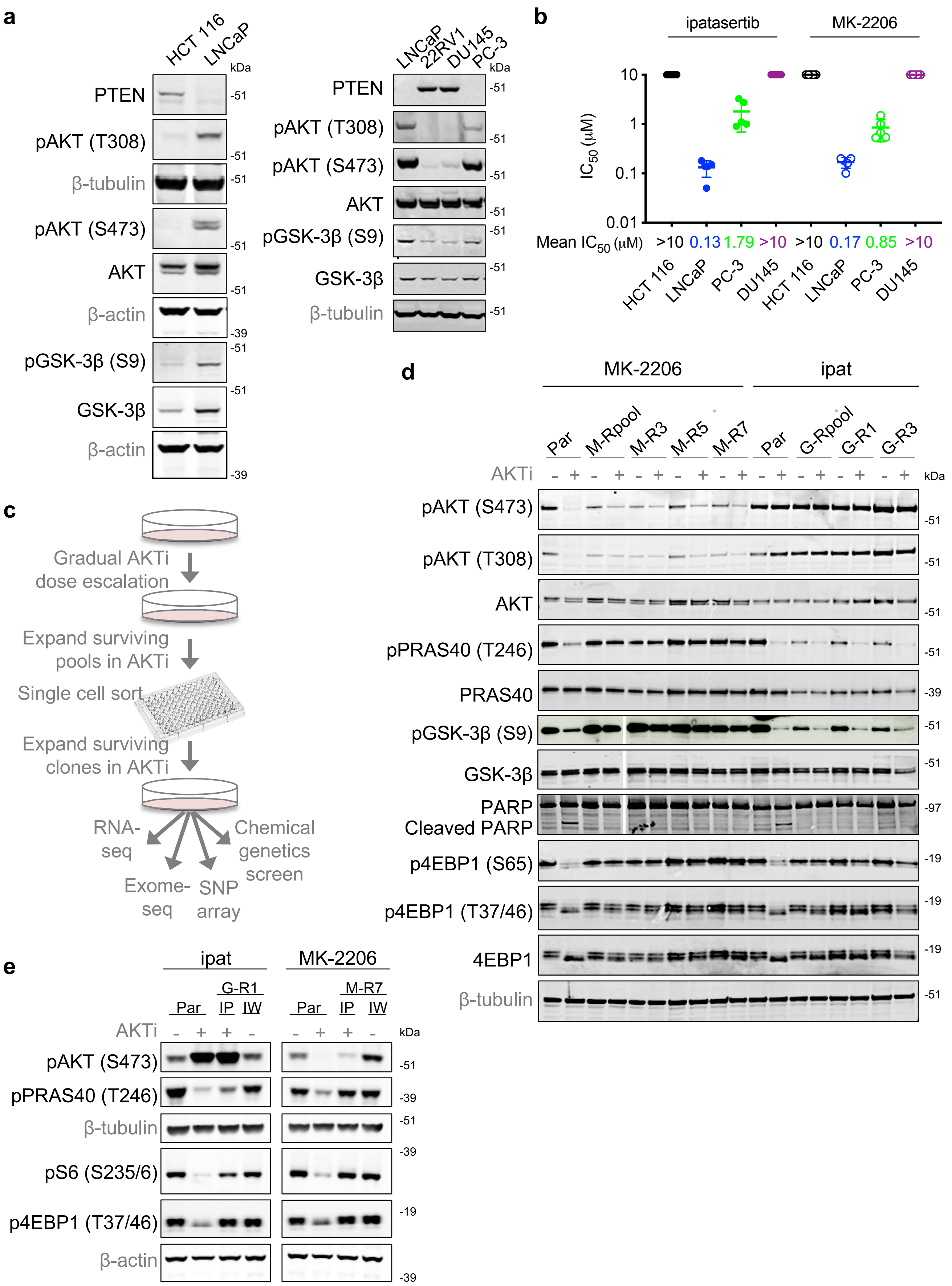
a
HCT 116
LNCaP
kDa
PTEN
pAKT (T308)
β-tubulin
pAKT (S473)
AKT
β-actin
pGSK-3β (S9)
GSK-3β
β-actin
LNCaP
22RV1
DU145
PC-3
kDa
PTEN
pAKT (T308)
pAKT (S473)
AKT
pGSK-3β (S9)
GSK-3β
β-tubulin
-51
-39
b
ipatasertib
MK-2206
IC50 (µM)
10
1
0.1
0.01
Mean IC50 (µM)
>10 0.13 1.79 >10 >10 0.17 0.85 >10
HCT 116
LNCaP
PC-3
DU145
HCT 116
LNCaP
PC-3
DU145
c
Gradual AKTi dose escalation
Expand surviving pools in AKTi
Single cell sort
Expand surviving clones in AKTi
RNA-seq
Exome-seq
SNP array
Chemical genetics screen
d
MK-2206
ipat
Par
M-Rpool
M-R3
M-R5
M-R7
Par
G-Rpool
G-R1
G-R3
AKTi
- + - + - + - + - + - + - + - + - +
kDa
pAKT (S473)
pAKT (T308)
AKT
pPRAS40 (T246)
PRAS40
pGSK-3β (S9)
GSK-3β
PARP
Cleaved PARP
p4EBP1 (S65)
p4EBP1 (T37/46)
4EBP1
β-tubulin
-51
-39
-97
-19
e
ipat
MK-2206
Par
G-R1
IP
IW
Par
M-R7
IP
IW
AKTi
- + + -
- + + -
kDa
pAKT (S473)
pPRAS40 (T246)
β-tubulin
pS6 (S235/6)
p4EBP1 (T37/46)
β-actin
-51
-39
-19

f

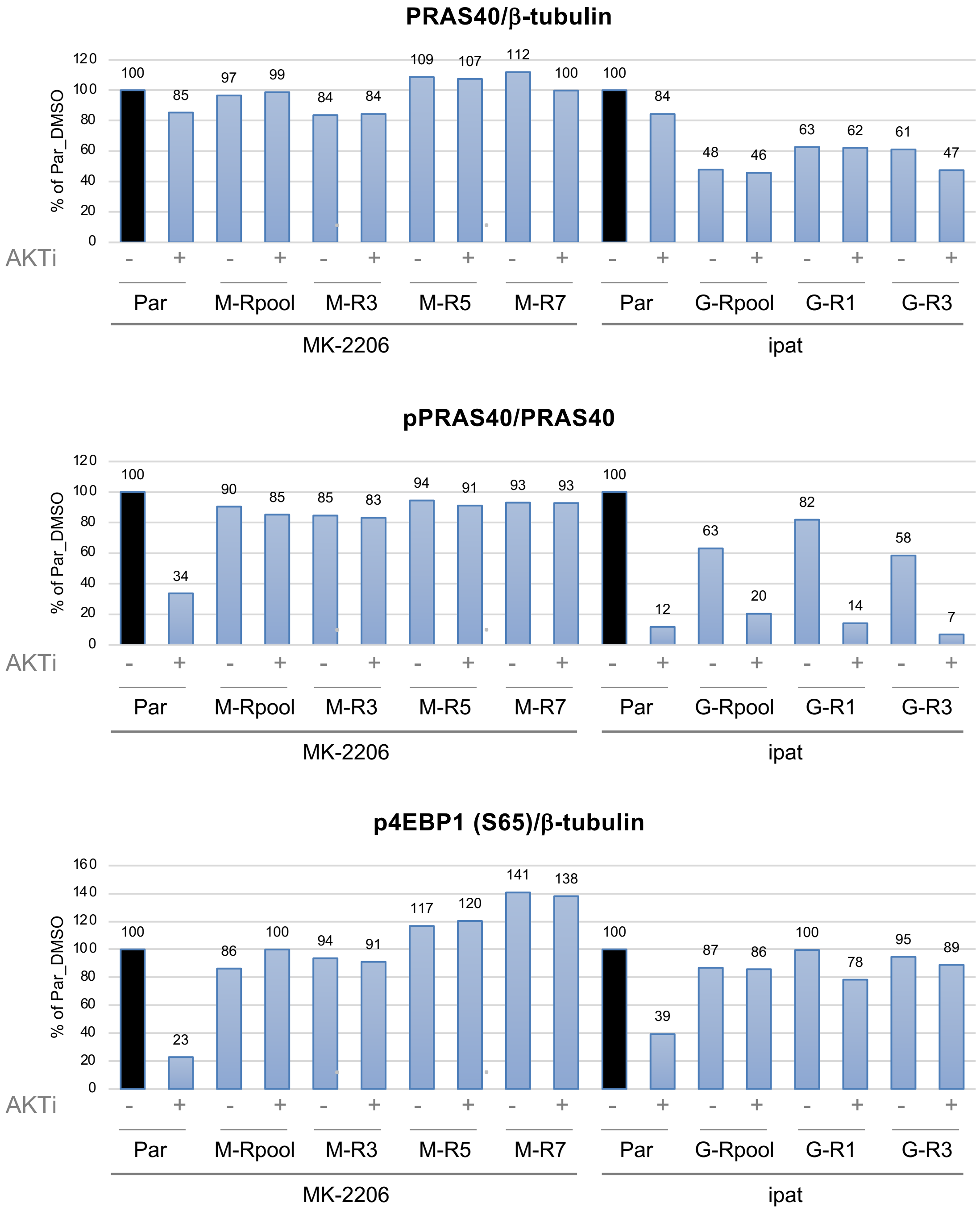

Supplementary Fig. 1, related to Fig. 1. Overview of LNCaP Par and AKTi-R cell characteristics. Par: parental, ipat: ipatasertib. **a,** Comparison of AKT pathway activity between LNCaP (PTEN-null), HCT 116 (PTEN WT), 22RV1 (PTEN WT), DU145 (PTEN WT) and PC-3 (PTEN null) cells, as measured by immunoblotting for PTEN, pAKT (T308), pAKT (S473) and pGSK-3β (S9) (a site phosphorylated by AKT). **b,** IC_{50} values of ipatasertib and MK2006 in the indicated cell lines as measured with a 4-day viability assay. Absolute IC_{50} values from independent experiments are plotted in scatter plots. Data are presented as Mean ± SD; n = 5 independent experiments. **c,** Schematic depicting establishment and analysis of AKTi-R lines. **d,** Levels of indicated total or phosphorylated proteins were assessed by immunoblotting in indicated cell lines after 5 μM of MK-2206 or ipat treatment for 3 hours. **e,** Immunoblotting of indicated markers in G-R1 and M-R7 cells cultured in the presence of the respective AKTi (IP), or with AKTi withdrawal for 11 passages (IW). Par cells treated with or without each AKTi for 16 hours are included for comparison. **f,** Quantification of total PRAS40, pPRAS40 and p4EBP1 (S65) in **d**, normalized to β-tubulin or total PRAS40 as indicated, and expressed as percentage of Par treated with DMSO. Source data are provided as a Source Data file.

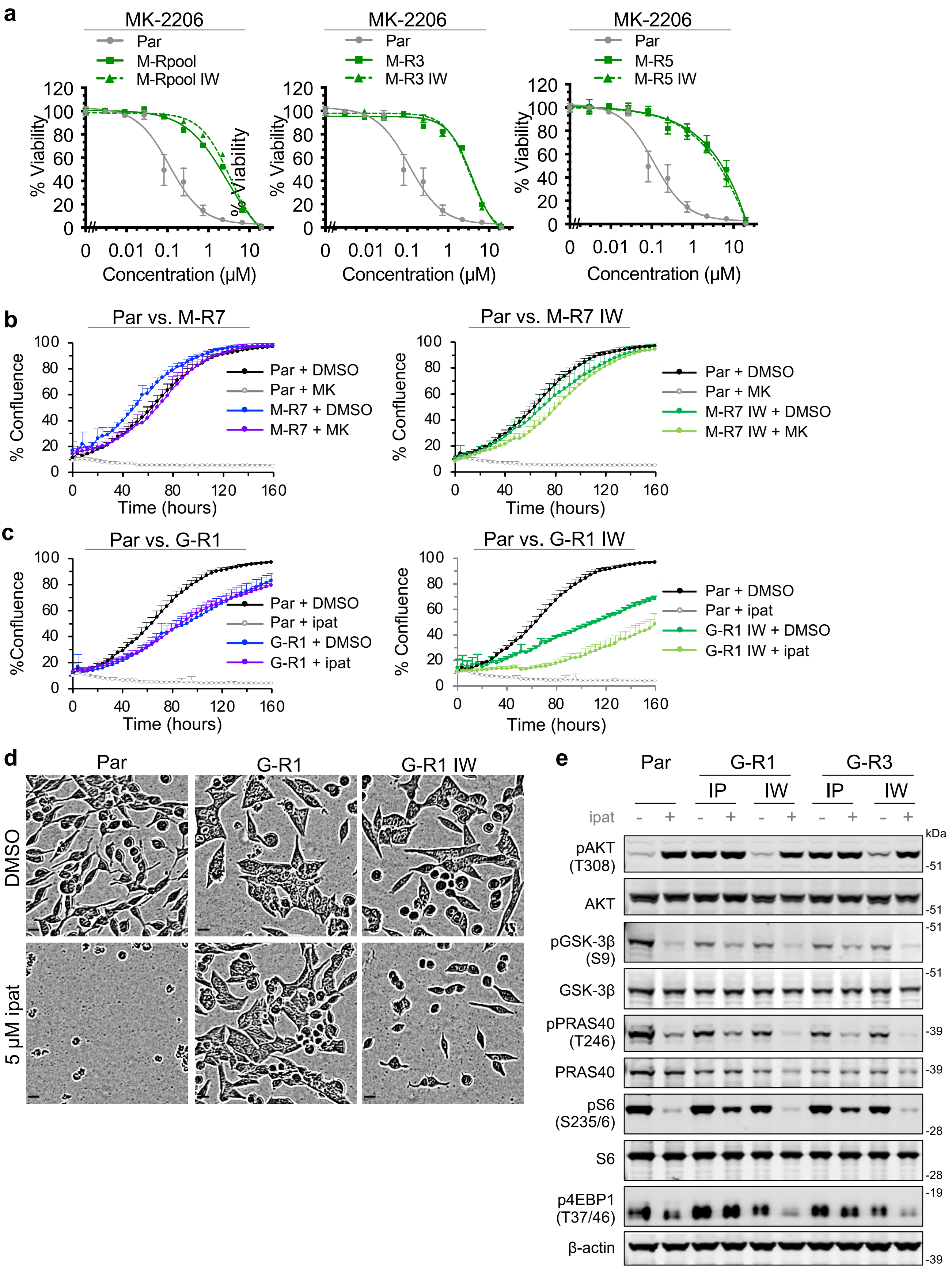
a
MK-2206
Par
M-Rpool
M-Rpool IW
M-R3
M-R3 IW
M-R5
M-R5 IW
% Viability
Concentration (µM)
b
Par vs. M-R7
Par vs. M-R7 IW
% Confluence
Time (hours)
Par + DMSO
Par + MK
M-R7 + DMSO
M-R7 + MK
M-R7 IW + DMSO
M-R7 IW + MK
c
Par vs. G-R1
Par vs. G-R1 IW
%Confluence
Par + ipat
G-R1 + DMSO
G-R1 + ipat
G-R1 IW + DMSO
G-R1 IW + ipat
d
Par
G-R1
G-R1 IW
DMSO
5 µM ipat
e
G-R3
IP
IW
ipat
kDa
pAKT (T308)
AKT
pGSK-3β (S9)
GSK-3β
pPRAS40 (T246)
PRAS40
pS6 (S235/6)
S6
p4EBP1 (T37/46)
β-actin

Supplementary Fig. 2, related to Fig. 1. Partial reversion of AKTi resistance is observed in G-R but not M-R cells. a, The impact on MK-2206 **w**ithdrawal for 11 passages (IW) was assessed using a 4-day viability assay in additional M-R cell lines (see Figure 1**e**). Error bars represent SEM; n = 4 replicates. **b,c,** Parental (Par) or IW cell lines were plated in DMSO-containing medium in 384 well plates incubated at 37°C under 5% CO_2. The next day, cells were treated with DMSO control, 5 µM MK-2206 (MK) or 5 µM ipatasertib (ipat). M-R7 or G-R1 cells were plated in DMSO-containing medium or 5 µM MK or 5 µM ipat and maintained in this treatment throughout the experiment at 37°C under 5% CO_2. Beginning one day after cell plating, cells were imaged every 4 hours (2 images per well) for 160 hours using the IncuCyte® ZOOM live cell analysis system (Essen Bioscience) and a 10x objective. Images were subjected to confluence analysis and percent confluence values from 8 replicates per condition were averaged. Scatter plots depict percent confluence over time, with error bars indicating SD. Graphs at left compare data from Par with M-R7 (**b**) or G-R1 (**c**) cells treated with DMSO or MK (**b**) or ipat (**c**) while graphs at right compare data from Par with M-R7 IW (**b**) or G-R1 IW (**c**) cells treated with DMSO or MK (**b**) or ipat (**c**) (data associated with Par cells is identical in left and right graphs). **d,** Representative images from day 4 are presented. Images were cropped and adjusted using identical settings. Note that cellular morphology is altered in G-R1 cells in comparison with Par cells. Scale bars, 10 µm. **e,** Par, G-R1 or G-R3 cells that were either maintained continuously in the presence of 5 µM ipat (IP), or IW lines were plated in fresh medium (not containing AKTi). The following day, cells were treated with 5 µM ipatasertib and 3 hours later, cells were harvested and lysates were subjected to immunoblot analysis of the indicated proteins. Source data are provided as a Source Data file.

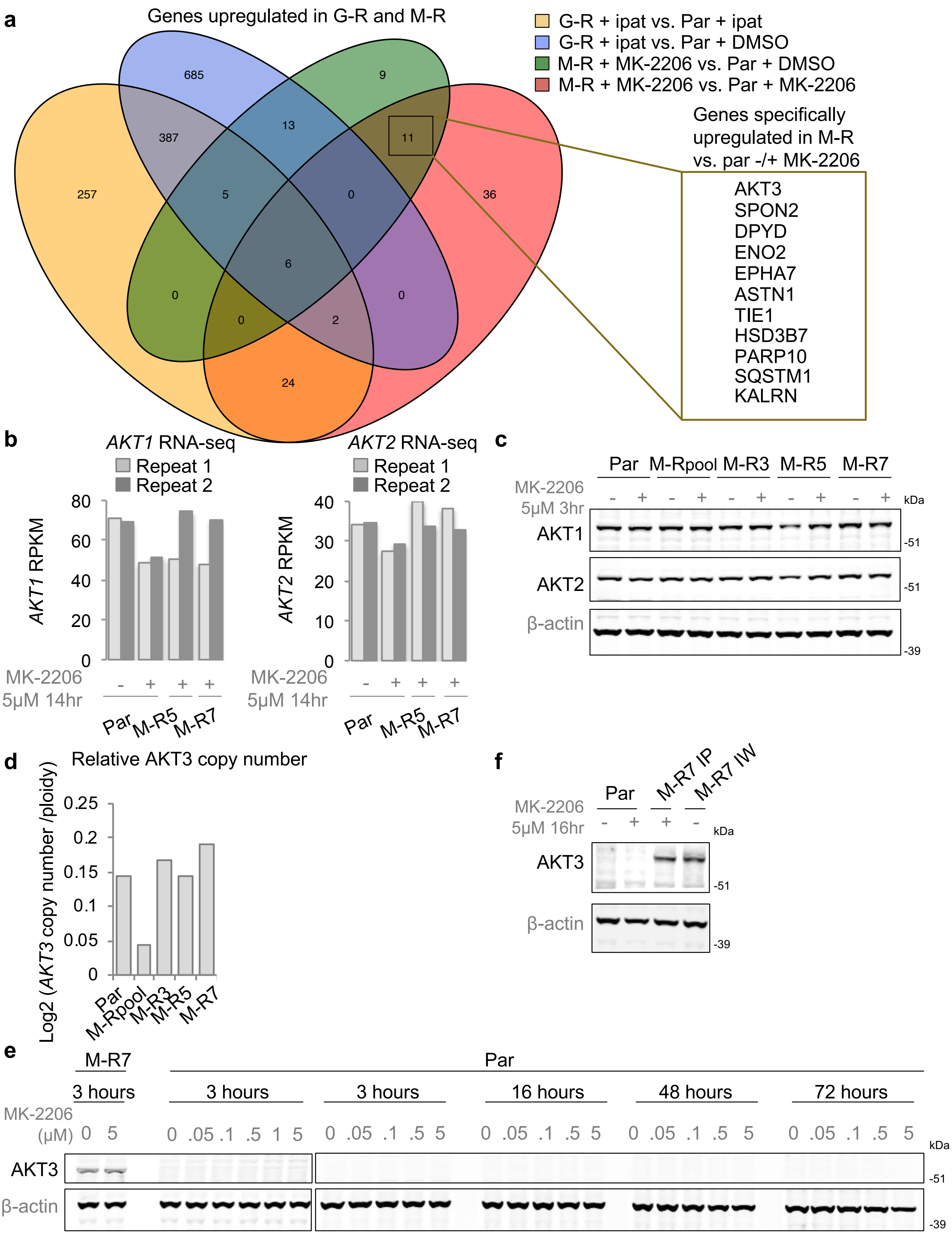
a
Genes upregulated in G-R and M-R
G-R + ipat vs. Par + ipat
G-R + ipat vs. Par + DMSO
M-R + MK-2206 vs. Par + DMSO
M-R + MK-2206 vs. Par + MK-2206
685
9
387
13
11
257
5
0
36
6
0
0
0
2
24
Genes specifically upregulated in M-R vs. par -/+ MK-2206
AKT3
SPON2
DPYD
ENO2
EPHA7
ASTN1
TIE1
HSD3B7
PARP10
SQSTM1
KALRN
b
AKT1 RNA-seq
Repeat 1
Repeat 2
AKT1 RPKM
MK-2206 5μM 14hr
Par
M-R5
M-R7
AKT2 RNA-seq
AKT2 RPKM
c
Par
M-Rpool
M-R3
M-R5
M-R7
MK-2206 5μM 3hr
kDa
AKT1
AKT2
β-actin
-51
-39
d
Relative AKT3 copy number
Log2 (AKT3 copy number /ploidy)
Par
M-Rpool
M-R3
M-R5
M-R7
f
Par
M-R7 IP
M-R7 IW
MK-2206 5μM 16hr
AKT3
β-actin
e
M-R7
Par
3 hours
16 hours
48 hours
72 hours
MK-2206 (μM)
0 5
0 .05 .1 .5 1 5
0 .05 .1 .5 5
AKT3
β-actin
kDa
-51
-39

Supplementary Fig. 3, related to Fig. 2. Identification of genes specifically upregulated in M-R vs. Par -/+ MK-2206 and AKT isoform expression and copy number in LNCaP and M-R cells. a, Venn diagram depicts the number of overlapping and nonoverlapping genes differentially upregulated in ipatasertib-treated G-R cells vs. DMSO or ipatasertib-treated par or MK-2206-treated M-R cells vs. DMSO or MK-2206-treated Par. Genes with adjusted *p*-value < 0.05 and absolute value of log2FC ≥ 1 were considered to be differentially expressed. Genes specifically upregulated in M-R vs. DMSO or MK-2206-treated Par cells are highlighted and listed. **b,** RPKM values associated with the *AKT1* and *AKT2* loci, as detected by RNA-seq, are plotted. **c,** Levels of indicated proteins after 3 hour treatment with 5μM MK-2206 were detected by immunoblot. **d,** *AKT3* DNA copy number was determined in Par or M-R cell lines using Illumina HumanOnmi2.5-8 arrays. Log2 values of of total *AKT3* copy number normalized to cell ploidy are plotted. **e,** M-R7 or Par cells were treated with the indicated doses of MK-2206 for indicated times and levels of indicated proteins were assessed by immunoblot. **f,** Levels of indicated proteins were assessed by immunoblot in DMSO or MK-2206-treated Par, M-R7 cells that were maintained continuously in the presence of 5 μM ipat (IP), or IW lines.

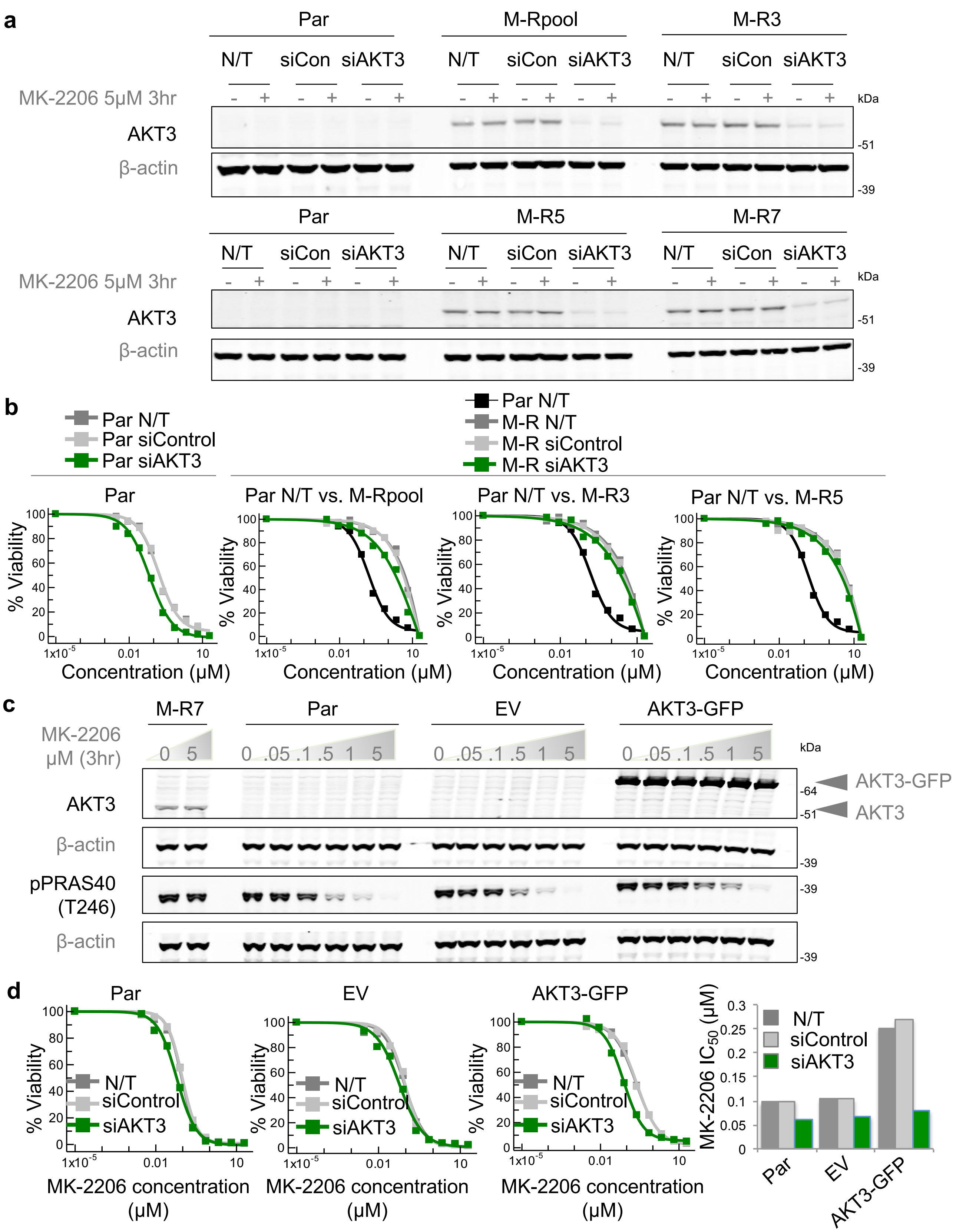
a
Par
M-Rpool
M-R3
N/T siCon siAKT3
MK-2206 5μM 3hr
- + - + - +
kDa
AKT3
β-actin
-51
-39
Par
M-R5
M-R7
b
Par N/T
Par siControl
Par siAKT3
Par N/T
M-R N/T
M-R siControl
M-R siAKT3
Par
Par N/T vs. M-Rpool
Par N/T vs. M-R3
Par N/T vs. M-R5
% Viability
Concentration (μM)
c
M-R7
EV
AKT3-GFP
MK-2206 μM (3hr)
0 5
0 .05 .1 .5 1 5
pPRAS40 (T246)
-64
AKT3-GFP
d
N/T
siControl
siAKT3
MK-2206 concentration (μM)
MK-2206 IC50 (μM)
Par
EV
AKT3-GFP

Supplementary Fig. 4, related to Fig. 2. The impact of AKT3 KD or overexpression on Par vs. additional M-R cell lines. a, AKT3 levels were assessed in non-transfected (N/T) or siRNA-transfected Par and M-R cell lines as described in Fig 2**d**. β-actin served as a loading control. **b,** The response of the indicated cells to MK-2206 was assessed following siRNA transfection as described in Figure 2**e**. Non-transfected controls were included. The dose response curve associated with non-transfected Par cells (Par N/T) was included in the three right graphs as a comparison. **c,** Par, EV, and AKT3-GFP cell lines were treated with DMSO control (0 μM MK-2206) or a range of MK-2206 concentrations (0.05-5 μM, increasing from left to right) and harvested 3 hours later for cell lysis. Lysates from M-R7 cells treated with 0 μM (DMSO only) or 5 μM MK-2206 for 3 hours were included as a comparison. Indicated total or phosphorylated proteins were then assessed by immunoblotting. β-actin served as a loading control. The first portion of the AKT3 and β-actin immunoblots (associated with M-R7 and Par cells) is the same displayed in the left portion of Figure S3**e**. **d,** The response of LNCaP Par, EV, or AKT3-GFP cells to MK-2206 following siRNA transfection vs. N/T was assessed as in Fig. 2**e**. Dose response curves and bar plot depicting absolute MK-2206 IC_{50} values calculated from dose response curves are presented. Error bars represent SEM; n = 4 replicates in **b** and **d**.

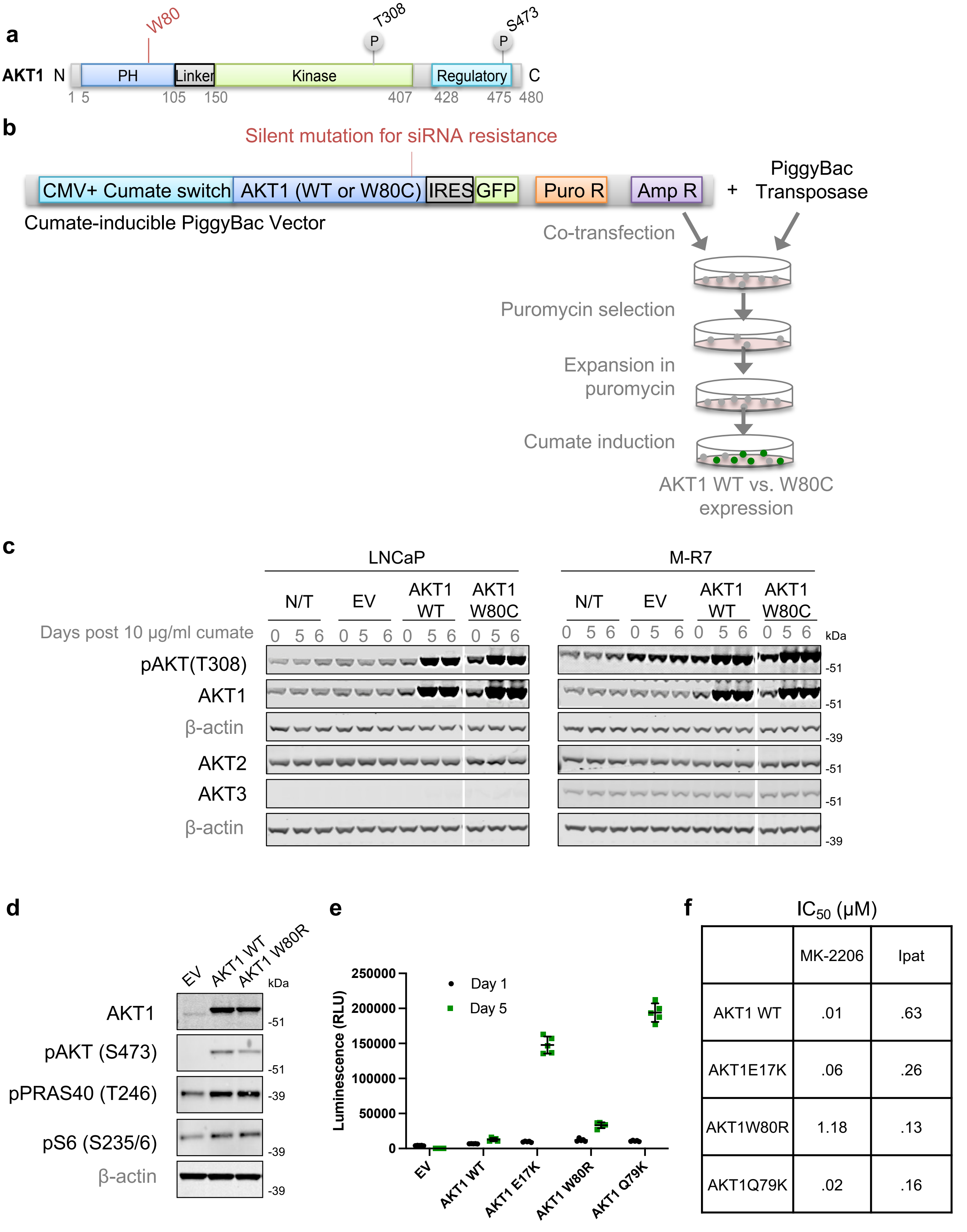

IC50 (µM)

	MK-2206	Ipat
AKT1 WT	.01	.63
AKT1E17K	.06	.26
AKT1W80R	1.18	.13
AKT1Q79K	.02	.16

Supplementary Fig. 5, related to Fig. 3. The AKT1 W80C mutation and its assessment using a cumate-inducible PiggyBac system. a, Schematic of human AKT1 domains and key activating phosphorylation sites, with the W80 residue (found to be mutated in M-R cells) indicated in red text. N: amino-terminus, C: carboxyl-terminus. **b,** Schematic describing cumate-inducible PiggyBac system used to examine the impact of expressing AKT1 WT vs. W80C. A silent mutation disrupting the binding site of siAKT1 was introduced in the sequence of the WT or W80C version of human AKT1. These sequences were then cloned into a PiggyBac expression vector which includes a cumate switch (enabling cumate-dependent expression of downstream genes), an IRES-GFP sequence (enabling co-expression of GFP and AKT1), as well as puromycin-resistance (PuroR) and ampicillin-resistance (Amp R) sequences for selection purposes. Cells (represented by grey solid circles in dishes) were co-transfected with the PiggyBac expression vector and the transposase and, 2 days later, subjected to puromycin selection. Cells were then expanded and maintained in the presence of puromycin. Upon induction with cumate, cells stably expressing the genes introduced via the PiggyBac system can be identified by GFP expression (indicated by green solid circles). **c,** LNCaP or M-R7 cells stably overexpressing the cumate-inducible AKT1 WT or W80C cells were treated with 10 μg/ml cumate for the indicated number of days and harvested for lysis. Cells expressing empty vector (EV) or non-transfected cells (N/T) were included as controls. Levels of indicated phosphorylated or total proteins were assessed by immunoblot. β-actin served as a loading control. Blots were cropped to display relevant lanes. All LNCaP lysates were run on the same blots, all M-R7 lysates were run on the same blots. **d,** Levels of indicated phosphorylated or total proteins were assessed by immunoblot in Ba/F3 cells stably overexpressing EV, AKT1 WT, or AKT1 W80R. β-actin served as a loading control. **e,** Luminescence values by CellTiter-Glo® assay from Ba/F3 cells co-expressing MEK1 N3 and EV, AKT1 WT, E17K, W80R or Q79K, 1 or 5 days following plating in the absence of IL3. Data are presented as Mean ± SD; n = 5 replicates. Representative data of 3 independent experiments are shown. **f,** Absolute IC50 values from data depicted in Fig. 3**g** are presented. Source data are provided as a Source Data file.

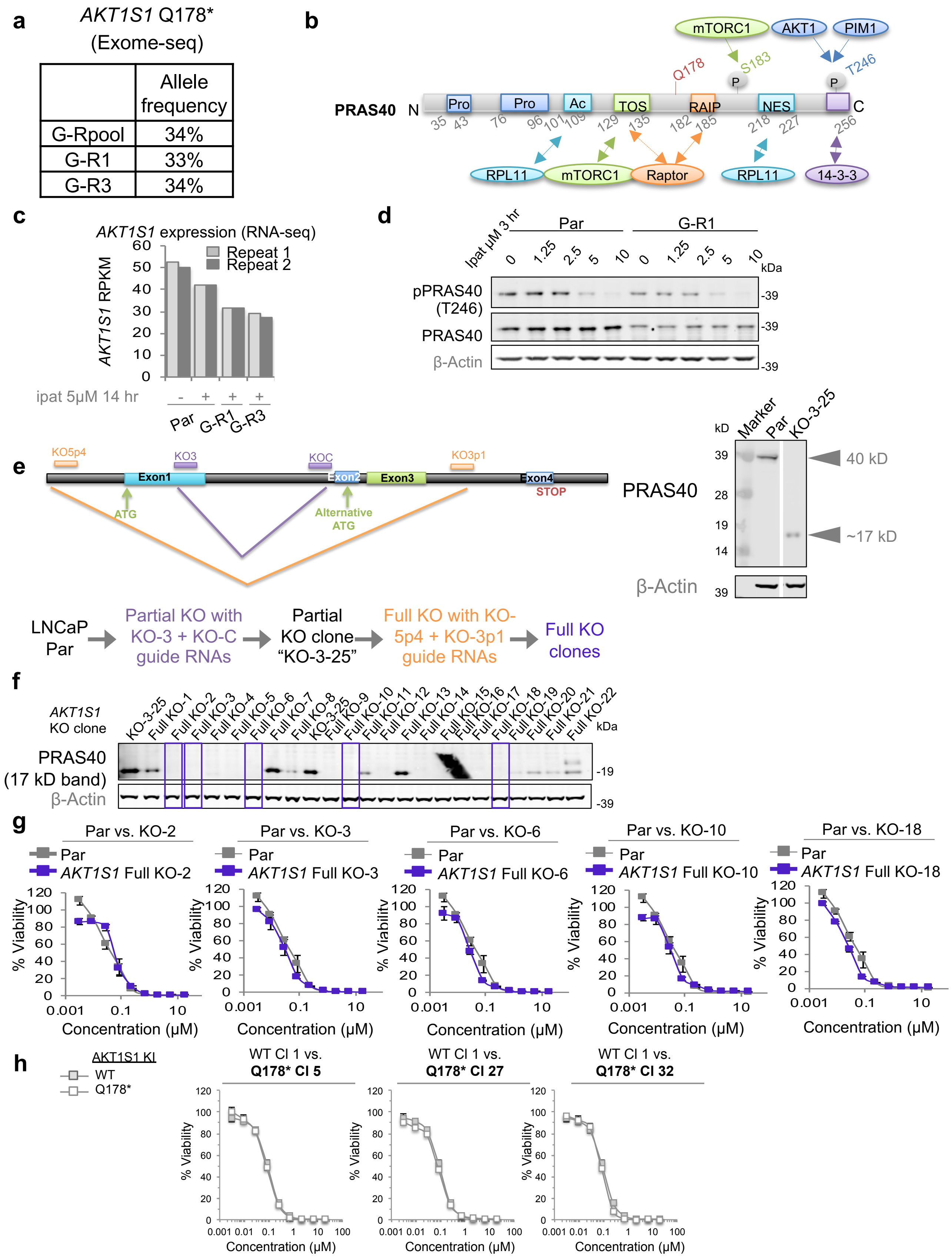
a
AKT1S1 Q178*
(Exome-seq)
Allele frequency
G-Rpool 34%
G-R1 33%
G-R3 34%
b
mTORC1
AKT1
PIM1
S183
T246
Q178
P
PRAS40
N
C
Pro
Pro
Ac
TOS
RAIP
NES
35 43 76 96 101 109 129 135 182 185 218 227 256
RPL11
mTORC1
Raptor
RPL11
14-3-3
c
AKT1S1 expression (RNA-seq)
AKT1S1 RPKM
Repeat 1
Repeat 2
ipat 5μM 14 hr
Par
G-R1
G-R3
d
Ipat μM 3 hr
Par
G-R1
0 1.25 2.5 5 10 0 1.25 2.5 5 10
kDa
pPRAS40 (T246)
PRAS40
β-Actin
-39
e
KO5p4
KO3
KOC
KO3p1
Exon1
Exon2
Exon3
Exon4
ATG
Alternative ATG
STOP
kD
Marker
Par
KO-3-25
PRAS40
40 kD
~17 kD
β-Actin
LNCaP Par
Partial KO with KO-3 + KO-C guide RNAs
Partial KO clone "KO-3-25"
Full KO with KO-5p4 + KO-3p1 guide RNAs
Full KO clones
f
AKT1S1 KO clone
KO-3-25
Full KO-1 … Full KO-8
KO-3-25
Full KO-9 … Full KO-22
kDa
PRAS40 (17 kD band)
β-Actin
-19
-39
g
Par vs. KO-2
Par vs. KO-3
Par vs. KO-6
Par vs. KO-10
Par vs. KO-18
Par
AKT1S1 Full KO-2
AKT1S1 Full KO-3
AKT1S1 Full KO-6
AKT1S1 Full KO-10
AKT1S1 Full KO-18
% Viability
Concentration (μM)
h
AKT1S1 KI
WT
Q178*
WT Cl 1 vs. Q178* Cl 5
WT Cl 1 vs. Q178* Cl 27
WT Cl 1 vs. Q178* Cl 32
% Viability
Concentration (μM)

Supplementary Fig. 6, related to Fig. 4. *AKT1S1*/PRAS40 Q178* mutation in G-R cells and effect of CRISPR/Cas9-mediated KO of *AKT1S1* or knock-in of the *AKT1S1* Q178* mutation in LNCaP cells. Par: parental, ipat: ipatasertib. **a,** As in Figure 3**b** except table depicts allele frequency of *AKT1S1* Q178* mutation in G-R cell lines. **b,** Schematic of domains, key mTORC1 or AKT1/PIM1 phosphorylation sites (S183 and T246, respectively), and the RPL11, mTORC1, Raptor, or 14-3-3 interaction sites (indicated by lines with double arrowheads) of human *AKT1S1*/PRAS40. Pro: proline-rich, Ac: acidic region, TOS: mTOR signaling motif, NES: Nuclear export signal, RPL11: ribosomal protein L11, N: amino-terminus, C: carboxyl-terminus. The Q178 residue altered in G-R cells is depicted in red. The Q178* mutation could result in expression of a truncated protein product lacking components including the RAIP domain (a motif critical for the interaction with Raptor), the NES (therefore impacting the interaction with RPL11 and perhaps subcellular localization), the interaction with 14-3-3 (perhaps impacting sequestering of PRAS40), mTORC1-mediated phosphorylation at S183, and AKT or PIM1-mediated phosphorylation at T246. Alternatively, or possibly in addition to this, the presence of the premature truncating mutation may result in nonsense mediated decay and thus decreased overall expression. **c,** *AKT1S1* transcript levels detected by RNA-seq in Par or G-R cell lines are depicted as described in Figure 2**b**. A reduction in *AKT1S1* transcript levels can be detected in Par cells treated with ipat for 14 hr and a further reduction can be detected in G-R1 and G-R3 cells maintained in ipat. **d,** Expression of pPRAS40 (T246) or total PRAS40 levels was examined in Par or G-R1 cells treated with indicated concentration of ipat for 3 hr by immunoblot. β-actin served as a loading control. The pPRAS40 (T246) and total PRAS40 bands presented here are associated with molecular weight (MW) of 40 kilodaltons (kDa) and therefore likely represent WT protein. A reduction in both phosphorylated and total PRAS40 can be detected in G-R1 cells in comparison to Par. No bands for the predicted MW for truncated PRAS40 Q178* (25 kDa) could be clearly detected in G-R cells in this or other experiments using a monoclonal antibody raised to the full length protein (Invitrogen/ThermoFisher #AHO1031). This may reflect that truncated protein is not expressed or only expressed at very low levels (below the limit of detection) or that the antibody does not recognize the truncated form. **e-g,** CRISPR/Cas9-mediated knockout (KO) of the *AKT1S1*/PRAS40 gene was performed. Initially, KO with paired guide RNAs (gRNAs) KO3 and KOC (depicted in schematic) resulted in expression of a ~17 kD protein recognized by the PRAS40 antibody (expression in clone KO3-25 is demonstrated in immunoblot data). Analysis of potential alternative transcriptional start sites resulting in in-frame truncated protein expression revealed an alternative ATG within exon 2 and downstream of the original gRNA pair. Therefore, as depicted in the schematic, additional gRNAs, KO5p4 and KO3p1, were designed to KO a larger region of *AKT1S1* including the alternative start site identified. The partial KO clone KO3-25 was subjected to CRISPR/Cas9 editing with KO5p4 and KO3p1. We confirmed complete loss of PRAS40 protein expression (including loss of the ~17 kD band) in various clones including Full KO-2, 3, 6, 10 and 18 (as depicted in **f**). The response of each clone to ipat was then assessed with a 4 day viability assay and compared with that of Par cells. Full PRAS40 protein KO in LNCaP cells had little impact on the response to ipatasertib, as measured with this assay. **h,** CRISPR/Cas9-mediated knock-in of the *AKT1S1* Q178* mutation does not impact the response of LNCaP cells to ipatasertib as measured in a 4 day viability assay. Error bars represent SEM; n = 4 replicates in **g** and **h**.

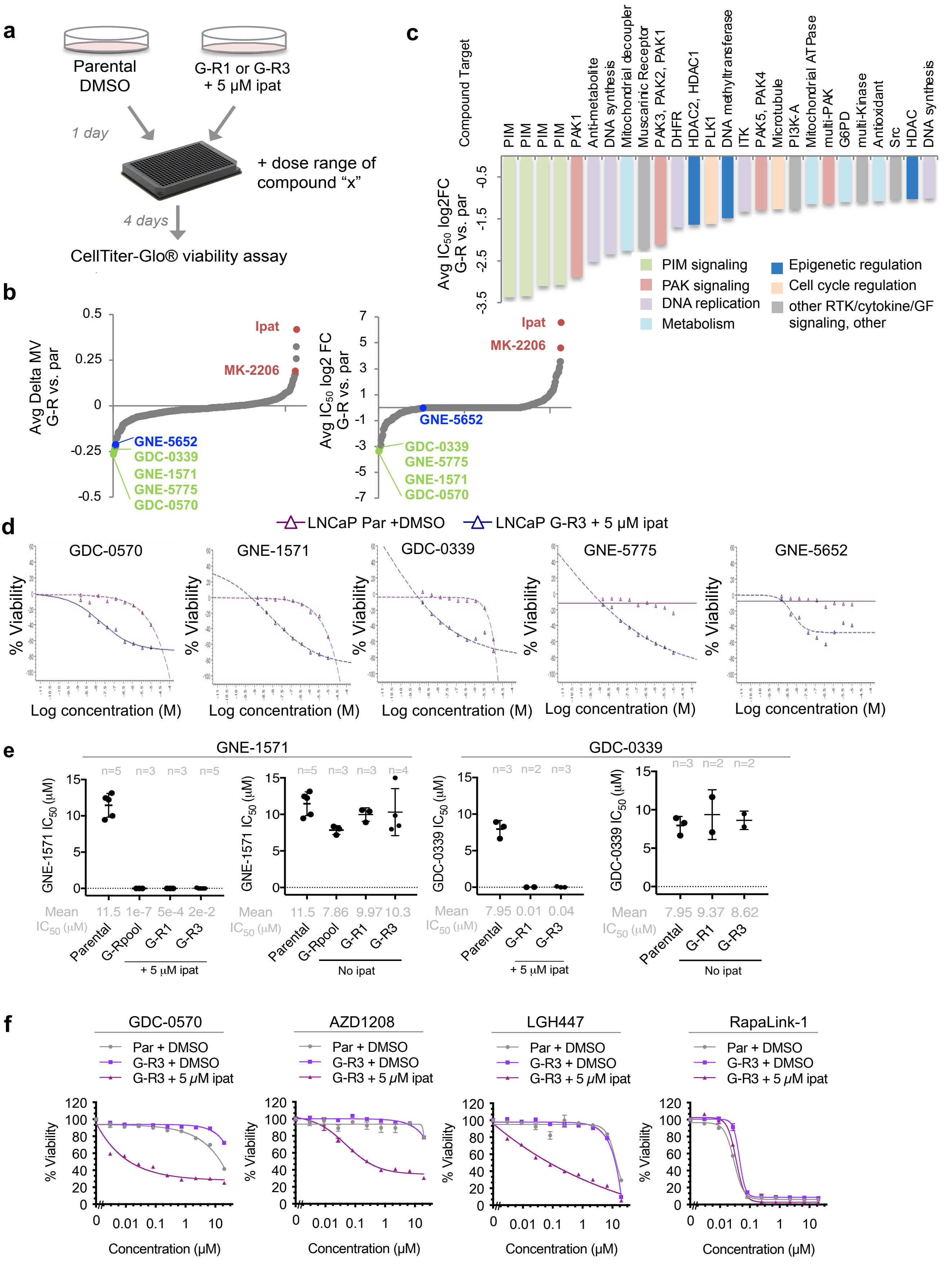

a
Parental DMSO
G-R1 or G-R3 + 5 μM ipat
1 day
+ dose range of compound "x"
4 days
CellTiter-Glo® viability assay
b
Avg Delta MV G-R vs. par
Ipat
MK-2206
GNE-5652
GDC-0339
GNE-1571
GNE-5775
GDC-0570
Avg IC50 log2 FC G-R vs. par
c
Compound Target
PIM
PIM
PIM
PIM
PAK1
Anti-metabolite
DNA synthesis
Mitochondrial decoupler
Muscarinic Receptor
PAK3, PAK2, PAK1
DHFR
HDAC2, HDAC1
PLK1
DNA methyltransferase
ITK
PAK5, PAK4
Microtubule
PI3K-A
Mitochondrial ATPase
multi-PAK
G6PD
multi-Kinase
Antioxidant
Src
HDAC
DNA synthesis
Avg IC50 log2FC G-R vs. par
PIM signaling
PAK signaling
DNA replication
Metabolism
Epigenetic regulation
Cell cycle regulation
other RTK/cytokine/GF signaling, other
d
LNCaP Par +DMSO
LNCaP G-R3 + 5 μM ipat
GDC-0570
GNE-1571
GDC-0339
GNE-5775
GNE-5652
% Viability
Log concentration (M)
e
GNE-1571
GDC-0339
GNE-1571 IC50 (μM)
GDC-0339 IC50 (μM)
n=5 n=3 n=3 n=5
n=5 n=3 n=3 n=4
n=3 n=2 n=3
n=3 n=2 n=2
Mean IC50 (μM) 11.5 1e-7 5e-4 2e-2
Mean IC50 (μM) 11.5 7.86 9.97 10.3
Mean IC50 (μM) 7.95 0.01 0.04
Mean IC50 (μM) 7.95 9.37 8.62
Parental G-Rpool G-R1 G-R3
+ 5 μM ipat
No ipat
f
GDC-0570
AZD1208
LGH447
RapaLink-1
Par + DMSO
G-R3 + DMSO
G-R3 + 5 μM ipat
% Viability
Concentration (μM)

Supplementary Fig. 7, related to Fig. 4. Design of the chemical genetics screen and the identification of PIM kinase inhibitors as screen hits. Par: parental, ipat: ipatasertib. **a,** Schematic of chemical genetics screen procedure (see text and experimental methods for description and more details). **b,** Scatter plot depicts average mean viability difference (Avg Delta MV, left) or average IC_{50} log2 fold change (Avg IC_{50} log2 FC, right) values of G-R1 and G-R3 cells vs. par for each inhibitor included in the screen. Values are plotted in ascending order. The values for ipat and MK-2206 are highlighted in red and labeled, and those for PIM kinase inhibitors included in the screen are highlighted in green and orange and labeled. **c,** Bar plot depicts average log2 fold change (FC) values of the chemical genetics screen hits. Compounds with average log2 FC ≤ -1 (i.e. > 2x greater sensitivity in the G-R cells relative to Par cells) are plotted in ascending order. Compound targets are indicated above bar plot. Colors correspond to pathways targeted by each compound as indicated in legend. The mean log2 FC of all compounds screened is 0.03 (see also Table S5). **d,** Dose response curves of all 5 PIM kinase inhibitors included in the library screen. The 5th PIM inhibitor, GNE-5652 (highlighted in blue in **b**), also showed significant reduction of cell viability in G-R3 cells compared to Par cells, although it did not reach 50% inhibition of viability in the fitted curve and hence was included as a hit in Figure **4a** but not Supplementary Fig. 7**c**. Par cells were plated in DMSO-containing medium, G-R3 cells were plated in 5 μM ipat-containing medium. **e,** The cellular response of G-Rpool, G-R1 and G-R3 cells (maintained in the presence of 5 μM ipat-containing medium) or Par cells to the PIMis GNE-1571 and GDC-0339 was assessed using a 4-day viability assay. IC_{50} values from independent experiments are depicted in scatter plots. Error bars represent SEM of the indicated number of independent experiments. **f,** Dose response of Par or G-R3 cells plated in DMSO-control medium or G-R3 cells plated in ipat-containing medium to the PIMis GDC-0570, AZD1208 or LGH447 or the mTORC1 kinase inhibitor RapaLink-1 was assessed using a 4-day viability assay. Error bars represent SEM; n = 4 replicates. Source data are provided as a Source Data file.

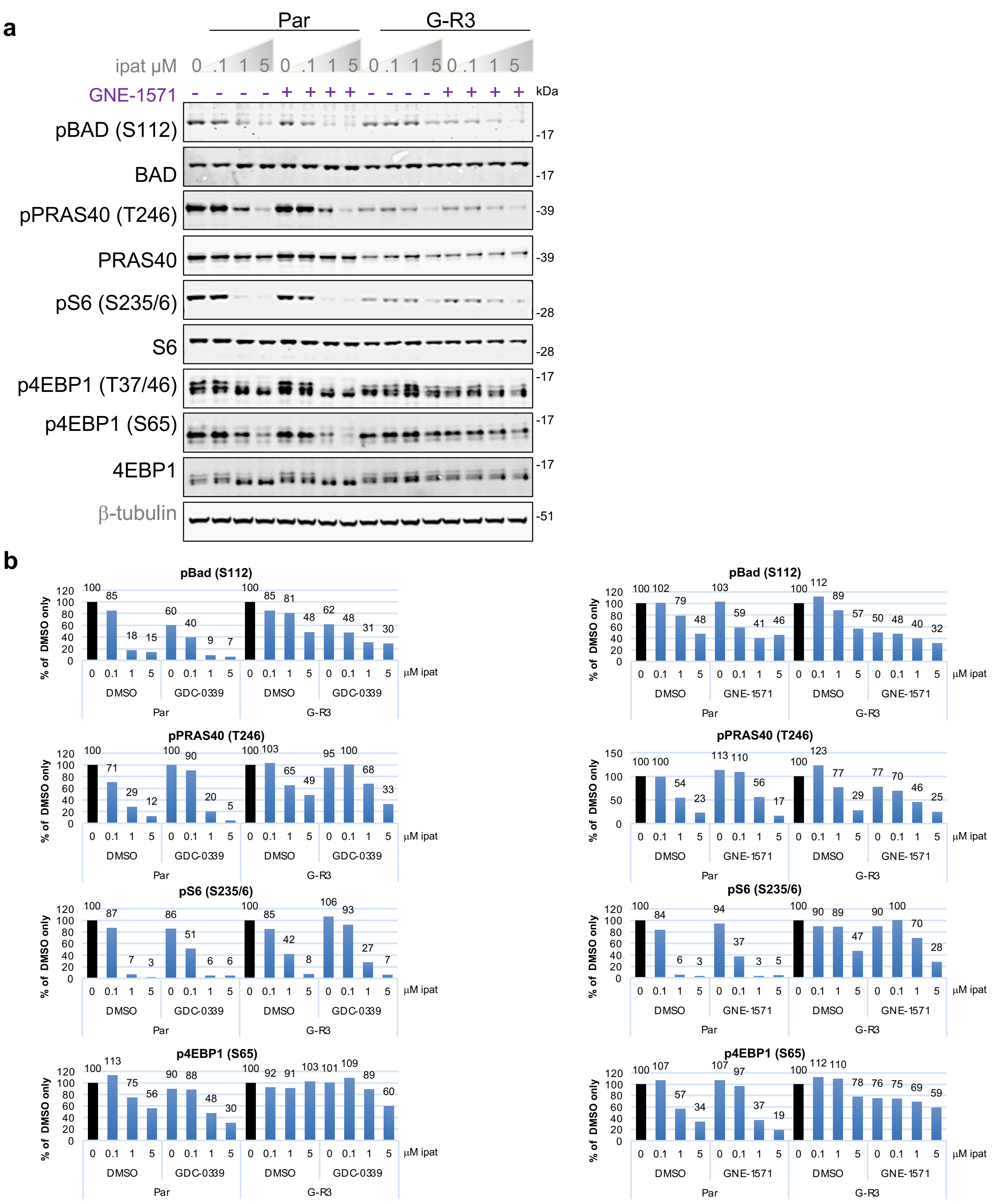

Supplementary Fig. 8, related to Fig. 4. Immunoblot analysis of the combination effects between ipat and PIMi in parental and G-R3 cells. a, Immunoblot analysis of the indicated proteins following treatment with the indicated concentrations of ipatasertib and 0.1 µM GNE-1571 for 24 hours, in Par or G-R3 cells. **b,** Quantification of the indicated protein markers shown in **a** (right) and Fig. 4**c** (left), normalized to β-actin (left) or β-tubulin (right) and expressed as percentage of the value in Par or G-R3 cells treated with DMSO only. Source data are provided as a Source Data file.

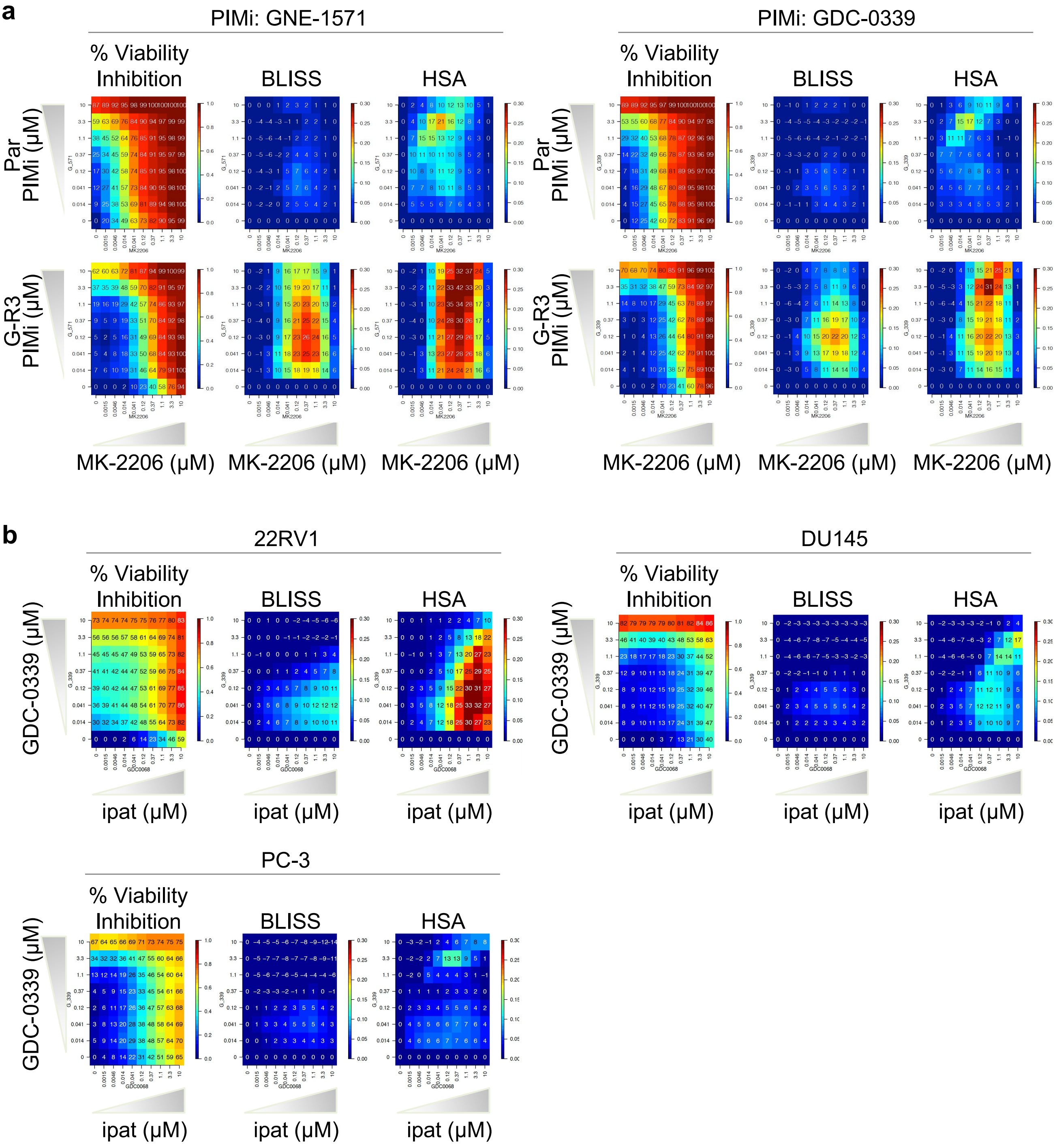

Supplementary Fig. 9, related to Fig. 4. Combination effects between AKTi and PIMi in prostate cancer cell lines. a, Heatmaps depict % viability inhibition, Bliss or HSA scores associated with each dose combination treatment of Par or G-R3 cells with MK-2206 and PIMi (GNE-1571 or GDC-0339). **b,** Heatmaps depict % viability inhibition, Bliss or HSA scores associated with each dose combination treatment of 22RV1, DU145 or PC-3 cells with ipatasertib and GDC-0339.

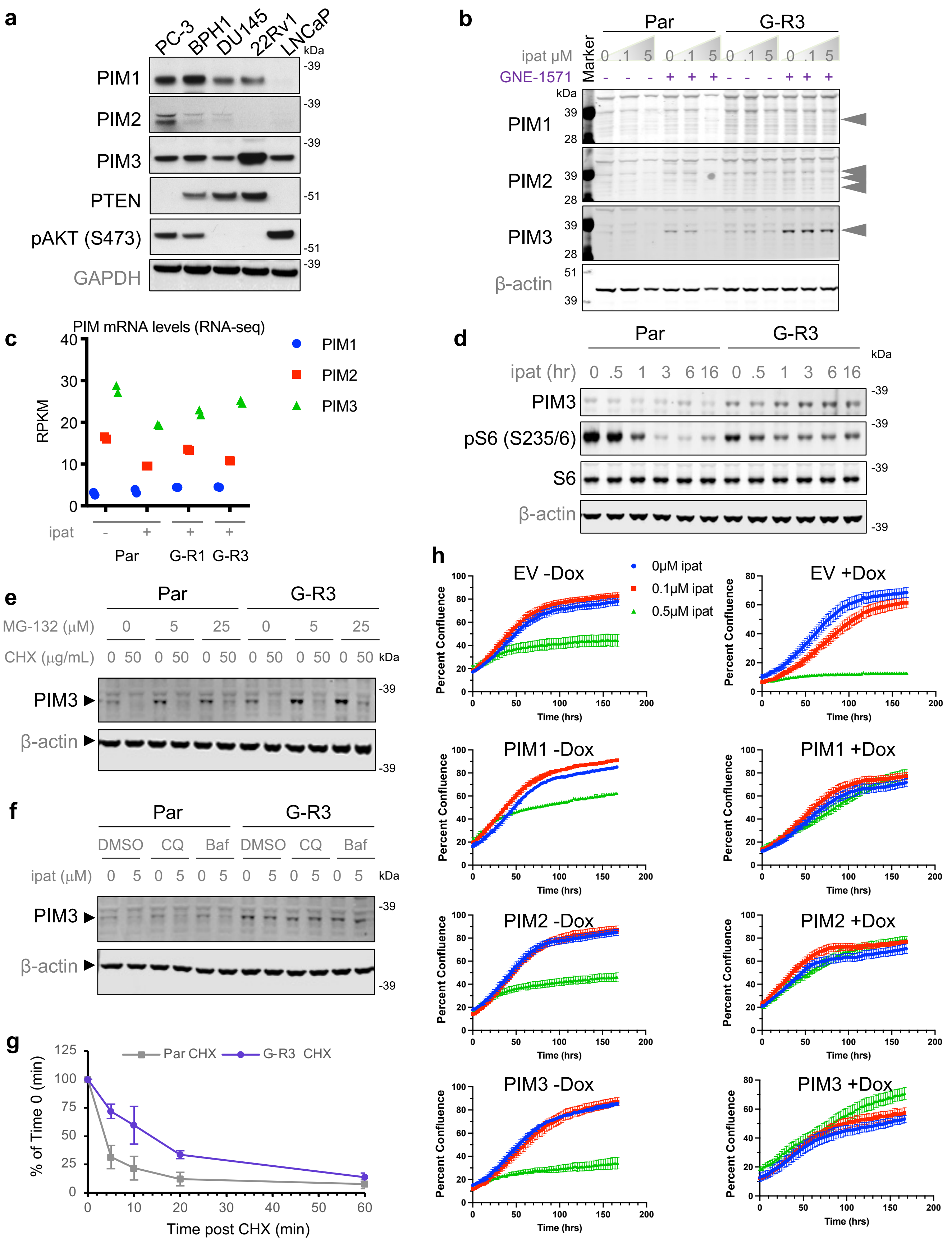
a
PC-3
BPH1
DU145
22Rv1
LNCaP
kDa
PIM1
PIM2
PIM3
PTEN
pAKT (S473)
GAPDH
-39
-51
b
Par
G-R3
ipat μM
0 .1 5
GNE-1571
Marker
kDa
PIM1
PIM2
PIM3
β-actin
39
28
51
c
PIM mRNA levels (RNA-seq)
RPKM
PIM1
PIM2
PIM3
ipat
Par
G-R1
G-R3
d
ipat (hr)
0 .5 1 3 6 16
PIM3
pS6 (S235/6)
S6
β-actin
e
MG-132 (μM)
0 5 25
CHX (μg/mL)
0 50
PIM3
β-actin
f
DMSO
CQ
Baf
ipat (μM)
0 5
g
Par CHX
G-R3 CHX
% of Time 0 (min)
Time post CHX (min)
h
EV -Dox
EV +Dox
PIM1 -Dox
PIM1 +Dox
PIM2 -Dox
PIM2 +Dox
PIM3 -Dox
PIM3 +Dox
0μM ipat
0.1μM ipat
0.5μM ipat
Percent Confluence
Time (hrs)

Supplementary Fig. 10, related to Fig. 5. Characterization of PIM1, 2, 3 expression and inducible PIM expression in LNCaP cells. Par: parental, ipat: ipatasertib. **a,** Protein levels of PIM1, 2, or 3 were assessed in the indicated prostate cell lines by immunoblot analysis. **b,** Protein levels of PIM1, 2, or 3 were assessed in Par or G-R3 cells treated with indicated concentrations of ipat with or without 0.1 μM of GNE-1571 for 24 hours by immunoblot analysis. Expected molecular weights of PIM1, 2 and 3 are indicated with grey arrow heads. **c,** RNA expression levels of PIM1, 2, or 3 was assessed in LNCaP par or G-R cells cultured in the absence or presence of 5 μM ipatasertib by RNA-seq. **d,** Par or G-R3 cells were treated with 5 μM ipatasertib and indicated protein levels were assessed by immunoblot. **e,** PIM3 protein levels in the presence or absence of 50 μg/mL cycloheximide (CHX) and the indicated concentrations of MG-132 for 2 hours in LNCaP Par or G-R3 cells. **f,** PIM3 protein levels in the presence or absence of 5 μM ipatasertib and the indicated compounds for 2 hours in LNCaP Par or G-R3 cells. CQ, chloroquine, 20 μM; Baf, bafilomycin A1, 10 nM. **g,** Quantification of PIM3 protein levels in Par and G-R3 cells normalized to β-tubulin at each time point post treatment with 50 μg/mL CHX, expressed as percentage of the levels at time 0. Error bars represent SEM; n = 3 independent experiments. Representative immunoblots are shown in Figure 5**b**. **h,** Dox-inducible expression of PIM1, 2, 3 reduced sensitivity to ipatasertib. LNCaP cells stably transfected with Dox-inducible empty vector (EV), PIM1, PIM2 or PIM3 were cultured in the presence of 100 ng/mL Dox for 3 days and sorted for median levels of GFP expression (driven by an IRES on the vector) and allowed to recover. The cells were plated in 384 well plates in the absence or presence of 100 ng/mL Dox and treated with DMSO, 0.1 or 0.5 μM ipatasertib and imaged every 2 hours (2 images per well) for 166 hours using the IncuCyte® ZOOM live cell analysis system (Essen Bioscience) and a 10x objective. Scatter plots depict percent confluence over time. Error bars represent SEM; n = 8 replicates. Representative data from 2 independent experiments are shown. Source data are provided as a Source Data file.

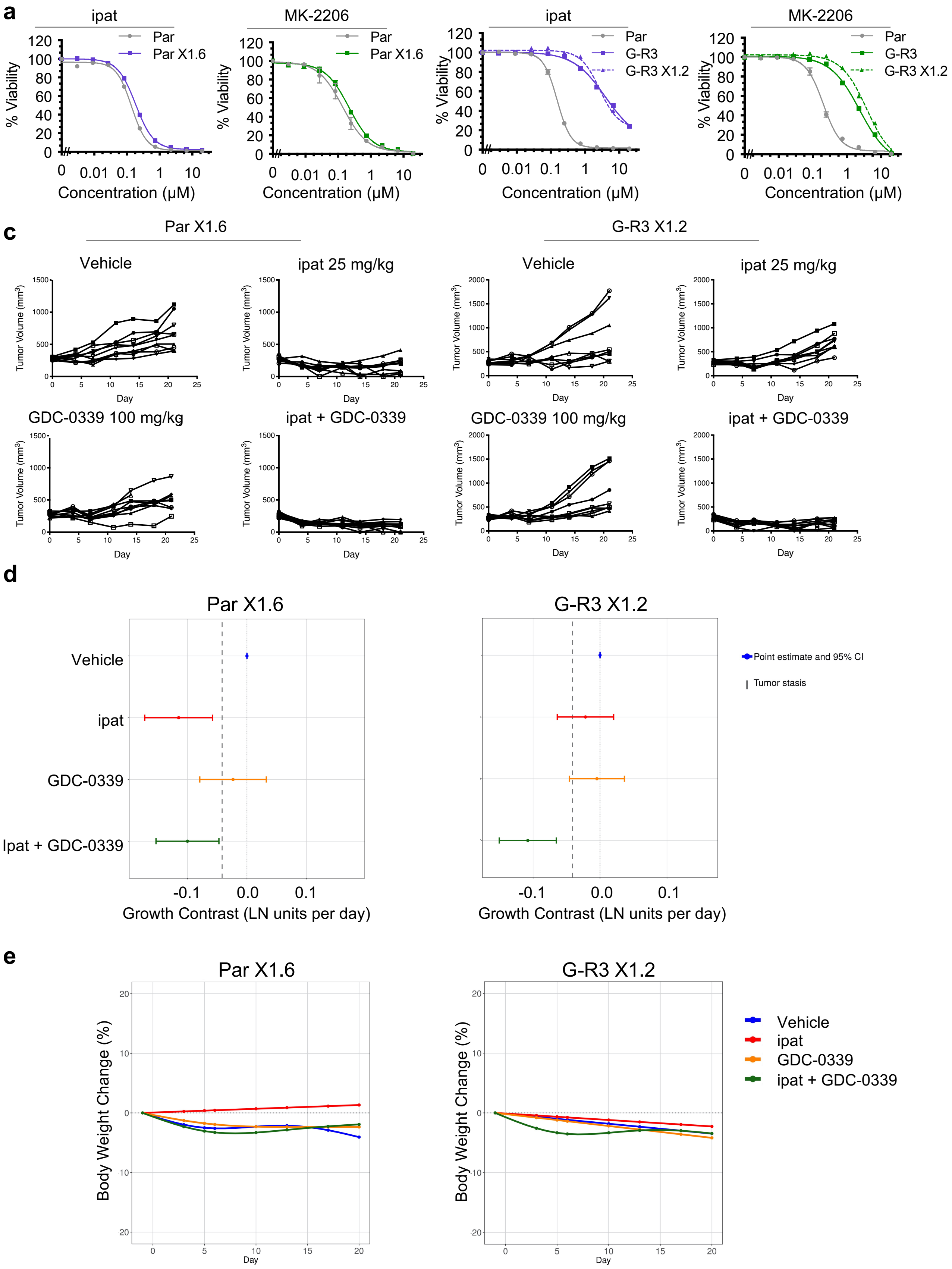
a
ipat
MK-2206
Par
Par X1.6
G-R3
G-R3 X1.2
% Viability
Concentration (μM)
c
Par X1.6
G-R3 X1.2
Vehicle
ipat 25 mg/kg
GDC-0339 100 mg/kg
ipat + GDC-0339
Tumor Volume (mm3)
Day
d
Par X1.6
G-R3 X1.2
Vehicle
ipat
GDC-0339
Ipat + GDC-0339
Point estimate and 95% CI
Tumor stasis
Growth Contrast (LN units per day)
e
Par X1.6
G-R3 X1.2
Body Weight Change (%)
Day
Vehicle
ipat
GDC-0339
ipat + GDC-0339

Supplementary Fig. 11, related to Fig. 6. Combined treatment with a PIMi overcomes resistance to ipatasertib *in vivo* in an ipatasertib-resistant model established *in vitro*. a, Representative AKTi dose response curves from a 4-day viability assay with LNCaP Par cells and the in vivo-selected line, Par X1.6. **b,** Representative AKTi dose response curves from a 4-day viability assay with LNCaP Par, G-R3, and the in vivo-selected G-R3 X1.2 cells. Error bars represent SEM; n = 4 replicates in **a** and **b**. **c,** Individual tumor volumes from the studies shown in Figure 6a. **d**, Tumor growth contrast between the treatment groups and the vehicle control group from the study in **c**. See Methods for details. **e,** Fitted percent body weight changes of mice in the vehicle control and treatment groups. Source data are provided as a Source Data file.

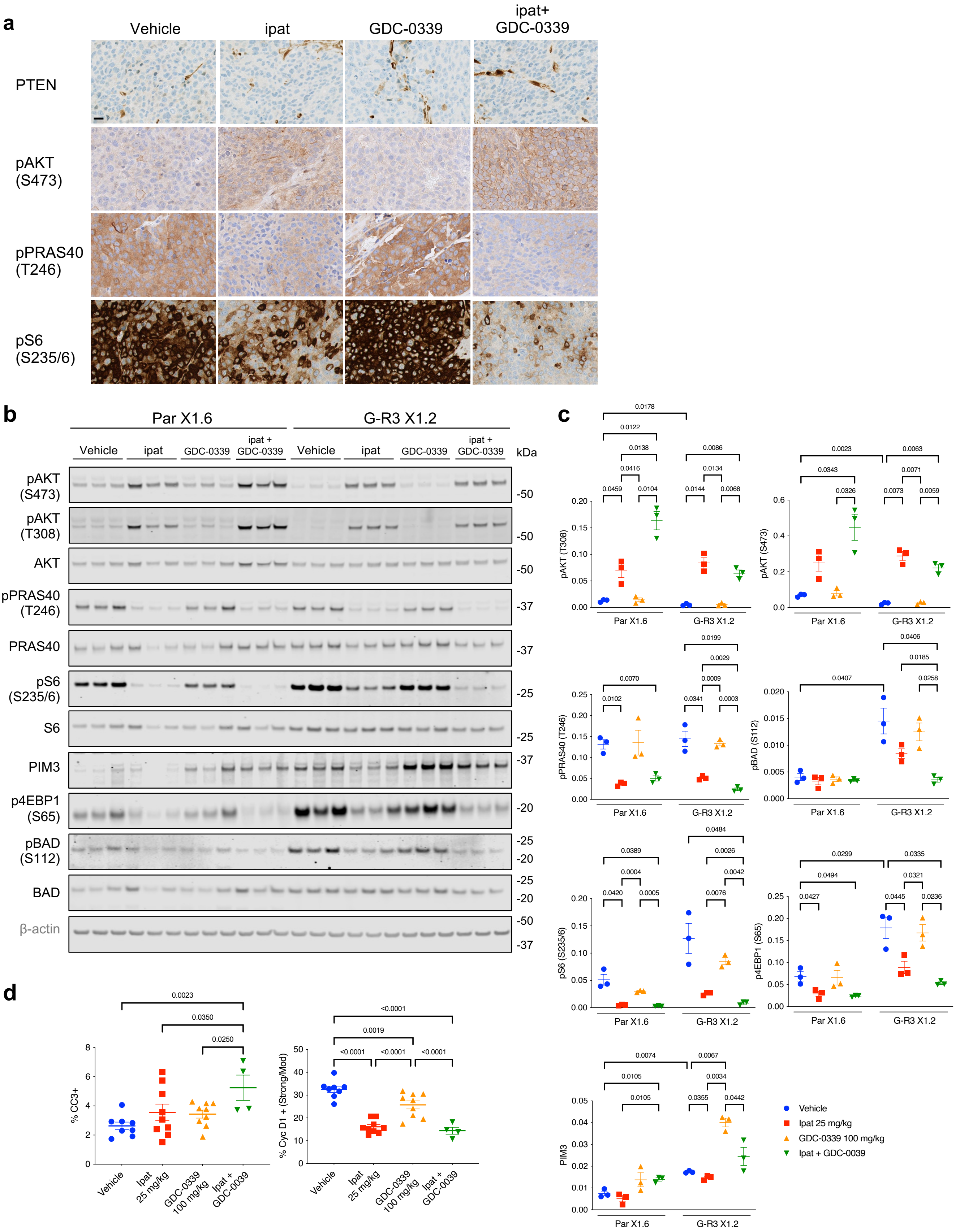
a
Vehicle
ipat
GDC-0339
ipat+ GDC-0339
PTEN
pAKT (S473)
pPRAS40 (T246)
pS6 (S235/6)
b
Par X1.6
G-R3 X1.2
Vehicle
ipat
GDC-0339
ipat + GDC-0339
kDa
pAKT (S473)
pAKT (T308)
AKT
pPRAS40 (T246)
PRAS40
pS6 (S235/6)
S6
PIM3
p4EBP1 (S65)
pBAD (S112)
BAD
β-actin
-50
-37
-25
-20
c
pAKT (T308)
pAKT (S473)
pPRAS40 (T246)
pBAD (S112)
pS6 (S235/6)
p4EBP1 (S65)
PIM3
Par X1.6
G-R3 X1.2
Vehicle
Ipat 25 mg/kg
GDC-0339 100 mg/kg
Ipat + GDC-0039
d
% CC3+
% Cyc D1 + (Strong/Mod)
Vehicle
Ipat 25 mg/kg
GDC-0339 100 mg/kg
Ipat + GDC-0039
0.0023
0.0350
0.0250
<0.0001
0.0019

Supplementary Fig. 12, related to Fig. 6. Biomarker analysis of the tumors. a, Representative immunohistochemistry images of the indicated markers in tumor samples collected 3 hours after the last dose of the indicated agents from the studies shown in Fig. 6a. Scale bar, 20 μM. **b,** Immunoblot analysis of the indicated markers in tumor lysates collected 3 hours after the last dose of the indicated agents from the studies shown in Fig. 6a**. c,** Quantification of immunoblots of the indicated markers normalized to β-actin**.** Error bars represent SEM; n = 3 tumors per treatment group; p values < 0.05 are indicated using Brown-Forsythe and Welch ANOVA tests, with unpaired t with Welch's correction and individual variances computed for each comparison. **d,** Quantification of IHC data with percentage of cleaved caspase 3 (CC3) positive tumor area normalized to total tumor nuclei area, and percentage of cyclin D1 (Cyc D1) strongly and moderately positive tumor area. Error bars represent SEM; n = 8, 9, 9, 4 tumors for vehicle, ipat, GDC-0339 and ipat + GDC-0339 treatment groups, respectively; p values < 0.05 are indicated using ordinary one-way ANOVA with uncorrected Fisher's LSD with a single pooled variance. Source data are provided as a Source Data file.

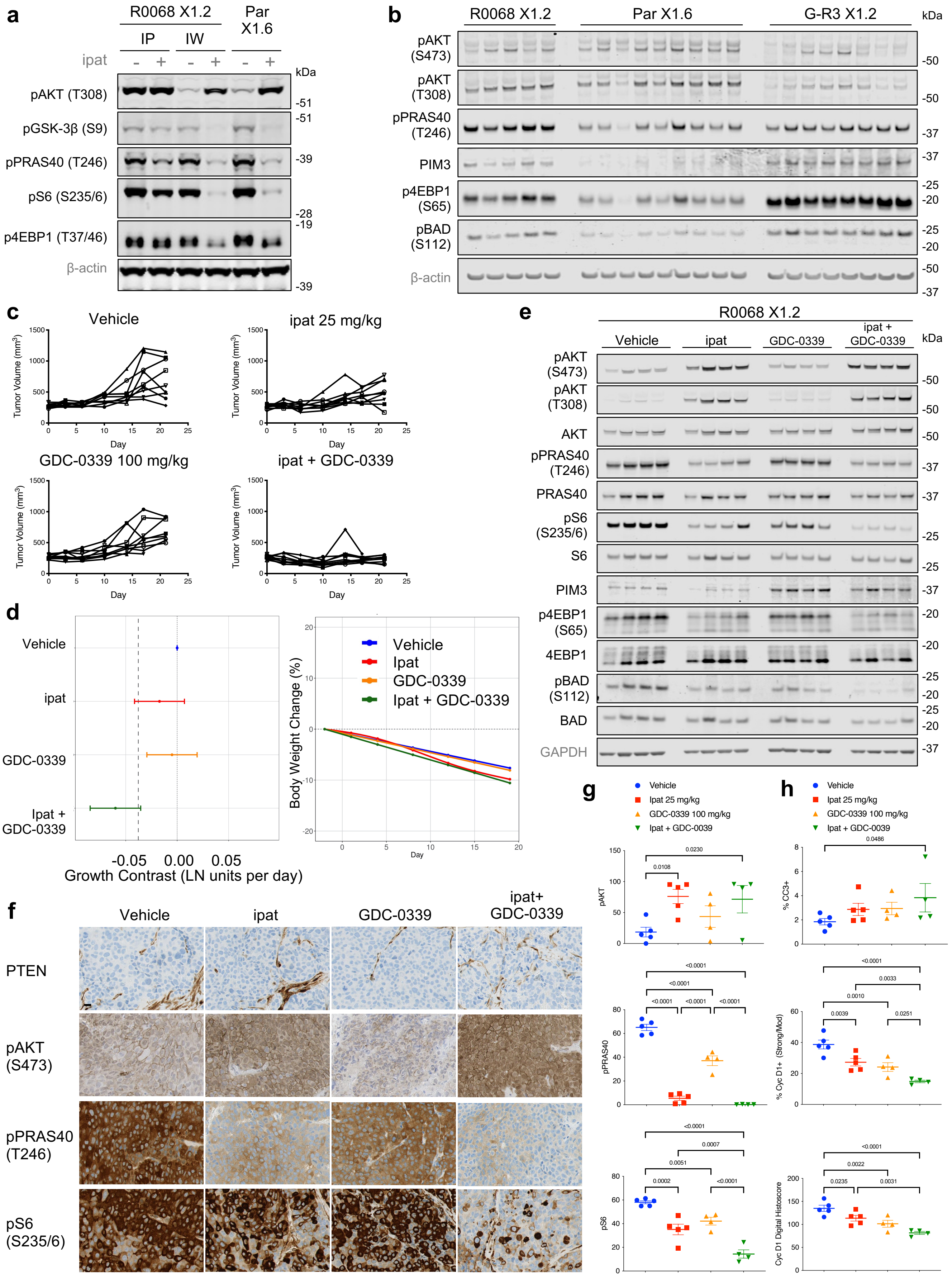
a
R0068 X1.2
IP
IW
Par X1.6
ipat
- + - + - +
kDa
pAKT (T308)
pGSK-3β (S9)
pPRAS40 (T246)
pS6 (S235/6)
p4EBP1 (T37/46)
β-actin
-51
-51
-39
-28
-19
-39
b
R0068 X1.2
Par X1.6
G-R3 X1.2
kDa
pAKT (S473)
pAKT (T308)
pPRAS40 (T246)
PIM3
p4EBP1 (S65)
pBAD (S112)
β-actin
-50
-50
-37
-37
-25
-20
-25
-20
-50
-37
c
Vehicle
ipat 25 mg/kg
GDC-0339 100 mg/kg
ipat + GDC-0339
Tumor Volume (mm³)
Day
d
Vehicle
ipat
GDC-0339
Ipat + GDC-0339
-0.05 0.00 0.05
Growth Contrast (LN units per day)
Body Weight Change (%)
Day
Vehicle
Ipat
GDC-0339
Ipat + GDC-0339
e
R0068 X1.2
Vehicle
ipat
GDC-0339
ipat + GDC-0339
kDa
pAKT (S473)
pAKT (T308)
AKT
pPRAS40 (T246)
PRAS40
pS6 (S235/6)
S6
PIM3
p4EBP1 (S65)
4EBP1
pBAD (S112)
BAD
GAPDH
-50
-50
-50
-37
-37
-25
-25
-37
-20
-20
-25
-20
-25
-20
-37
f
Vehicle
ipat
GDC-0339
ipat+ GDC-0339
PTEN
pAKT (S473)
pPRAS40 (T246)
pS6 (S235/6)
g
Vehicle
Ipat 25 mg/kg
GDC-0339 100 mg/kg
Ipat + GDC-0039
pAKT
0.0108
0.0230
pPRAS40
<0.0001
pS6
0.0002
0.0051
0.0007
<0.0001
h
Vehicle
Ipat 25 mg/kg
GDC-0339 100 mg/kg
Ipat + GDC-0039
% CC3+
0.0486
% Cyc D1+ (Strong/Mod)
0.0039
0.0010
0.0033
0.0251
<0.0001
Cyc D1 Digital Histoscore
0.0235
0.0022
0.0031
<0.0001

Supplementary Fig. 13, related to Fig. 6. Combined treatment with a PIMi overcomes resistance to ipatasertib *in vivo* in an ipatasertib-resistant model established *in vivo*. a, Indicated proteins were assessed by immunoblots following a 3-hour treatment with 5 μM ipatasertib in Par X1.6 or R0068 X1.2 cells either cultured continuously in the presence of ipat (IP) or with ipat withdrawal for 11 passages (IW). **b,** Immunoblot analysis of the indicated markers in tumors treated with vehicle only. **c,** Individual tumor volumes from the studies shown in Figure 6**e**. **d,** Tumor growth contrast between the treatment groups and the vehicle control group (left) and fitted percent body weight changes of mice in the vehicle control and treatment groups (right) from the study in **c**. **e,** Immunoblot analysis of the indicated markers in tumor lysates collected 3 hours after the last dose of indicated agents. **f,** Representative immunohistochemistry images of the indicated markers in tumor samples collected 3 hours after the last dose of indicated agents. Scale bar, 20 μM. **g,** Quantification of percent positive area per tumor area stained with each marker as in **f**. **h,** Quantification of percentage of cleaved caspase 3 (CC3) positive tumor area normalized to total tumor nuclei area, percentage of cyclin D1 (Cyc D1) strongly and moderately positive tumor area and cyclin D1 digital histoscore. Data are presented as Mean $\pm$ SEM; n = 5, 5, 4, 4 tumors for vehicle, ipat, GDC-0339 and ipat + GDC-0339 groups, respectively; p values < 0.05 are indicated using ordinary one-way ANOVA with uncorrected Fisher's LSD with a single pooled variance (**g-h**). Source data are provided as a Source Data file.

Appendix

Raw Immunoblot Images

c

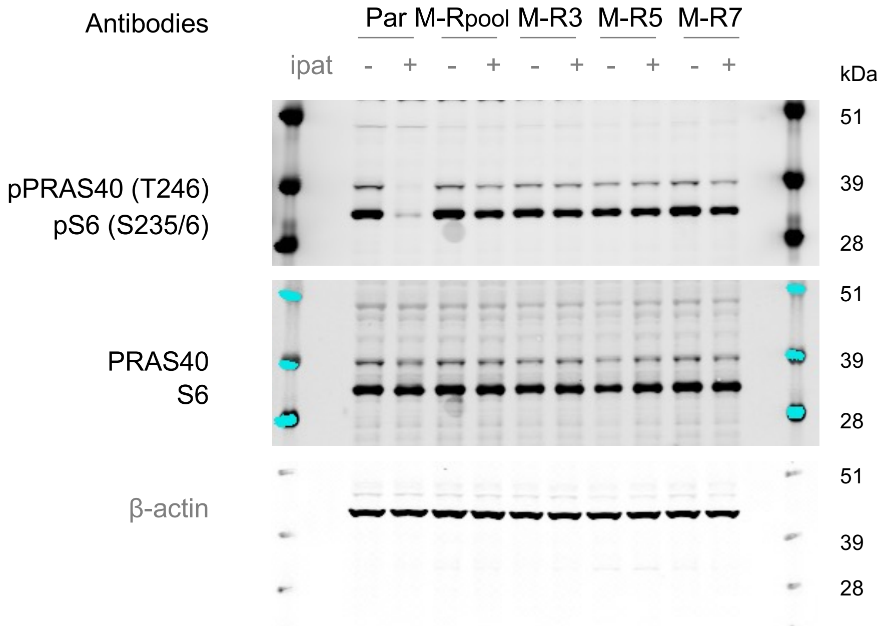

d

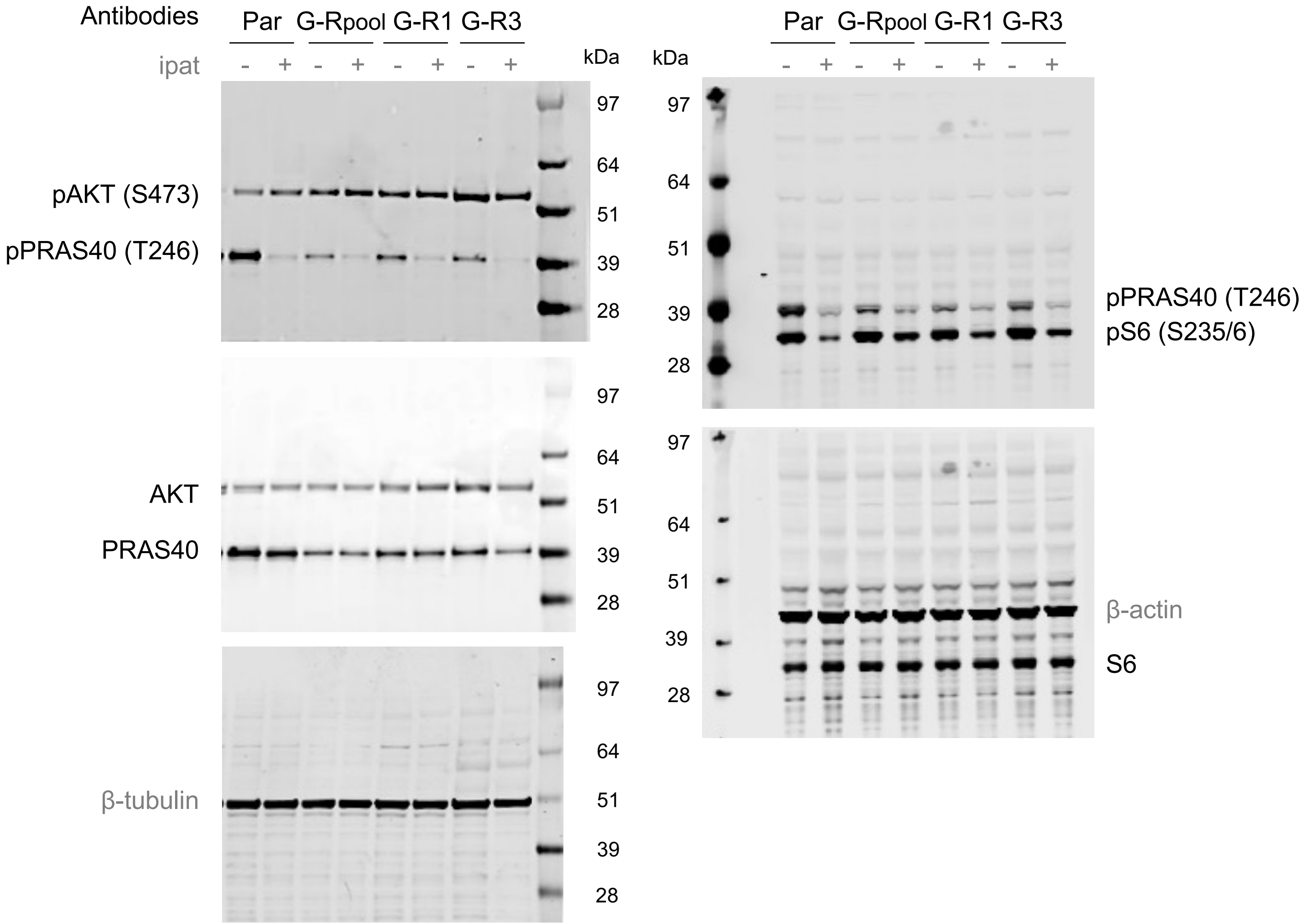

Blots cut below 51 kDa and then probed with the indicated antibodies

Figure 1c & d

c

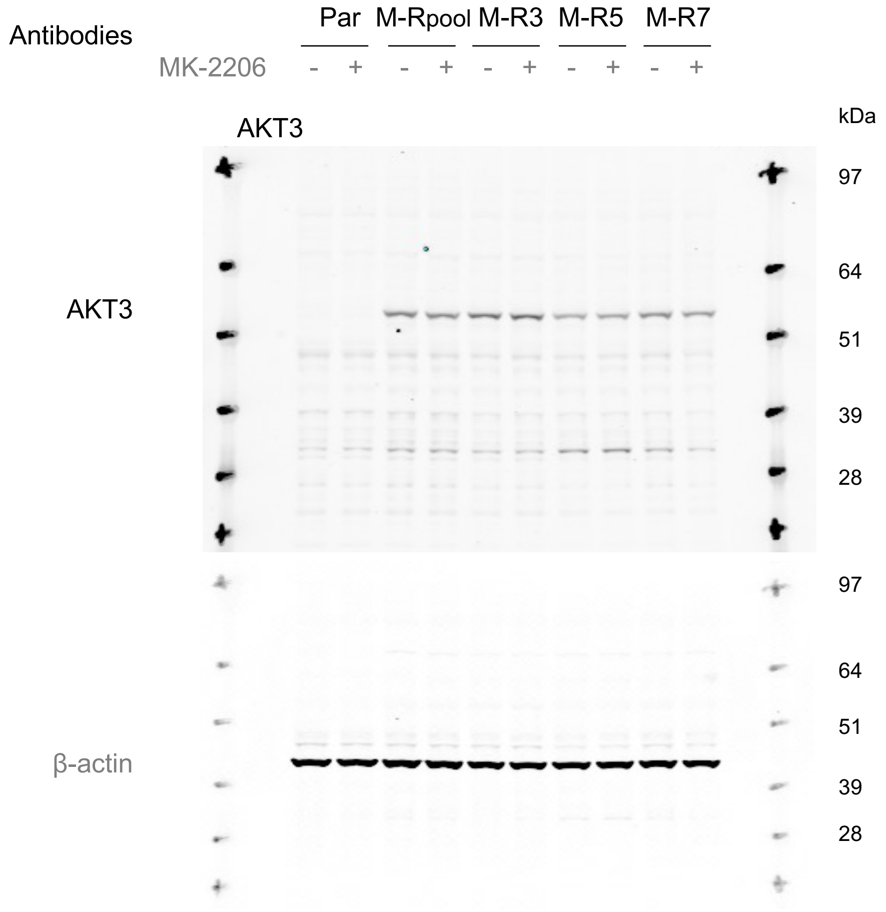

d

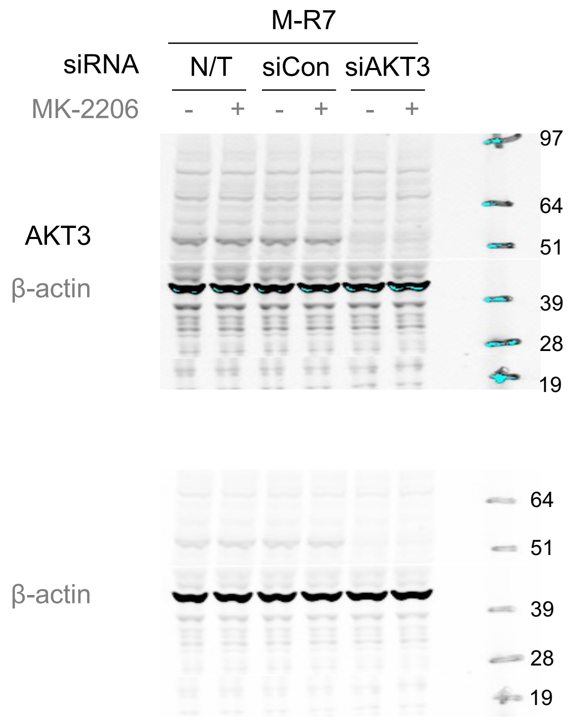

Figure 2c & d

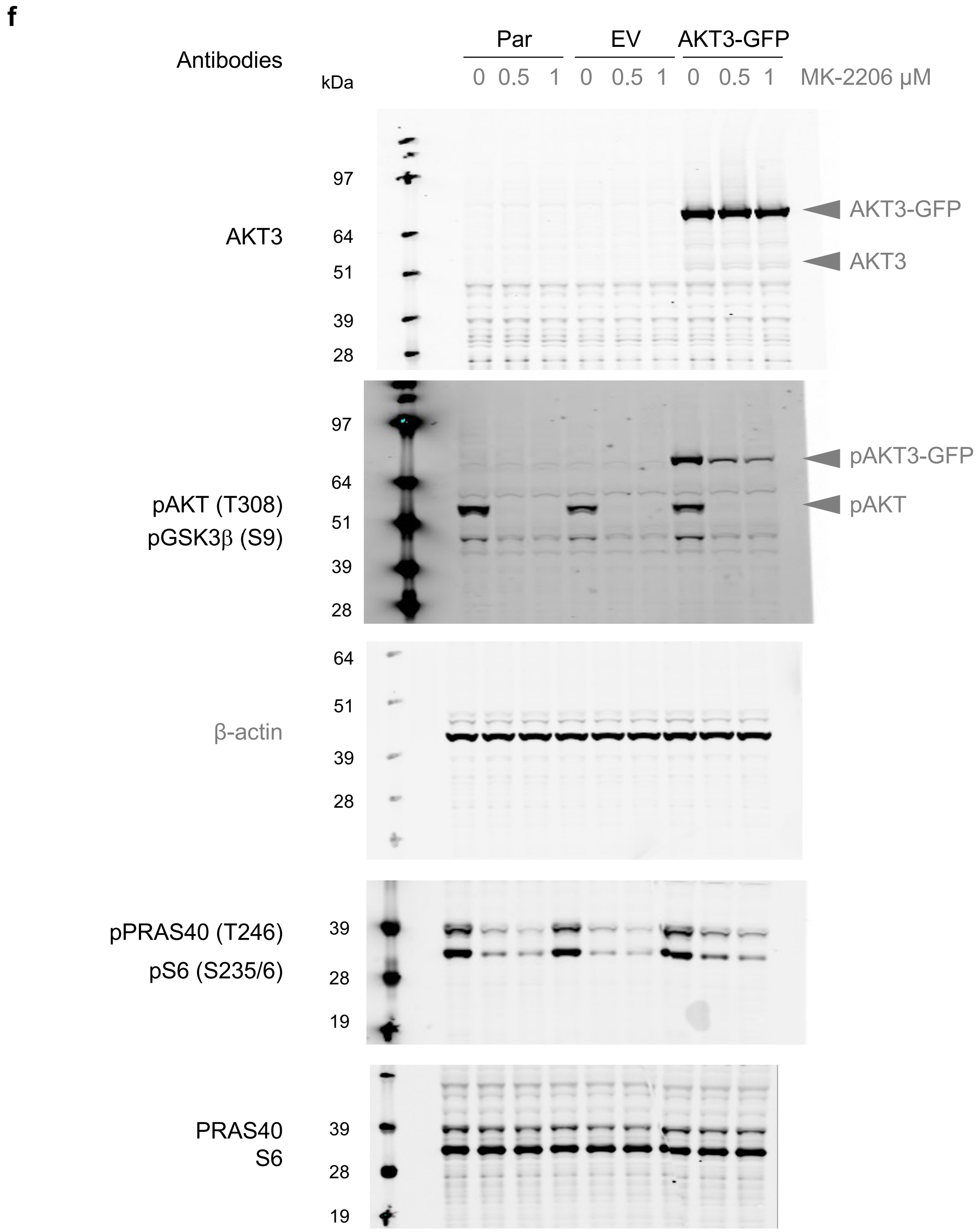
f
Antibodies
kDa
Par
EV
AKT3-GFP
0 0.5 1 0 0.5 1 0 0.5 1 MK-2206 µM
AKT3
97
64
51
39
28
AKT3-GFP
AKT3
pAKT (T308)
pGSK3β (S9)
97
64
51
39
28
pAKT3-GFP
pAKT
β-actin
64
51
39
28
pPRAS40 (T246)
pS6 (S235/6)
39
28
19
PRAS40
S6
39
28
19

Figure 2f

a

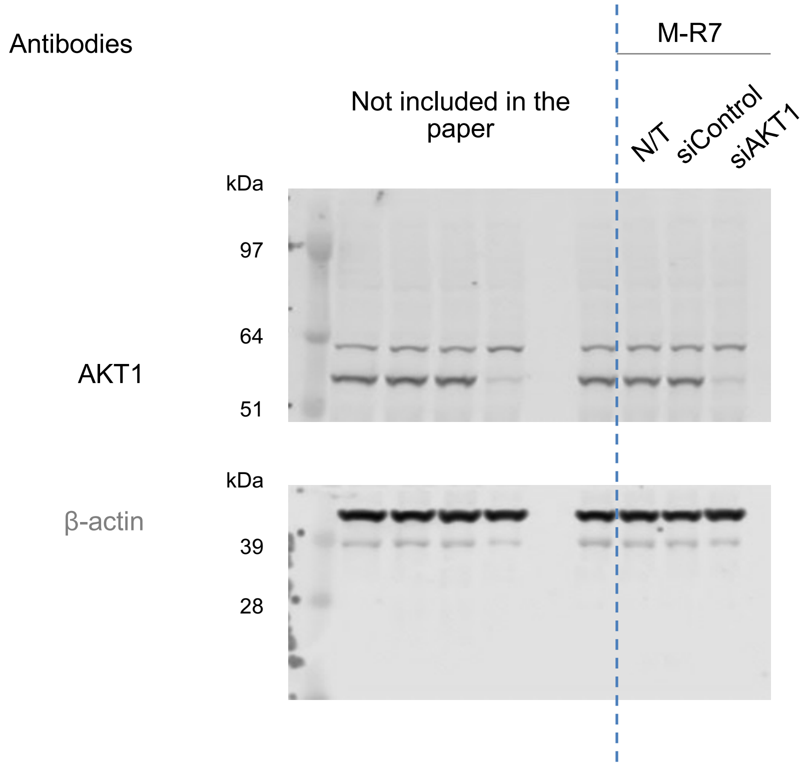

Blots cut below 51 kDa and then probed with the indicated antibodies

Figure 3a

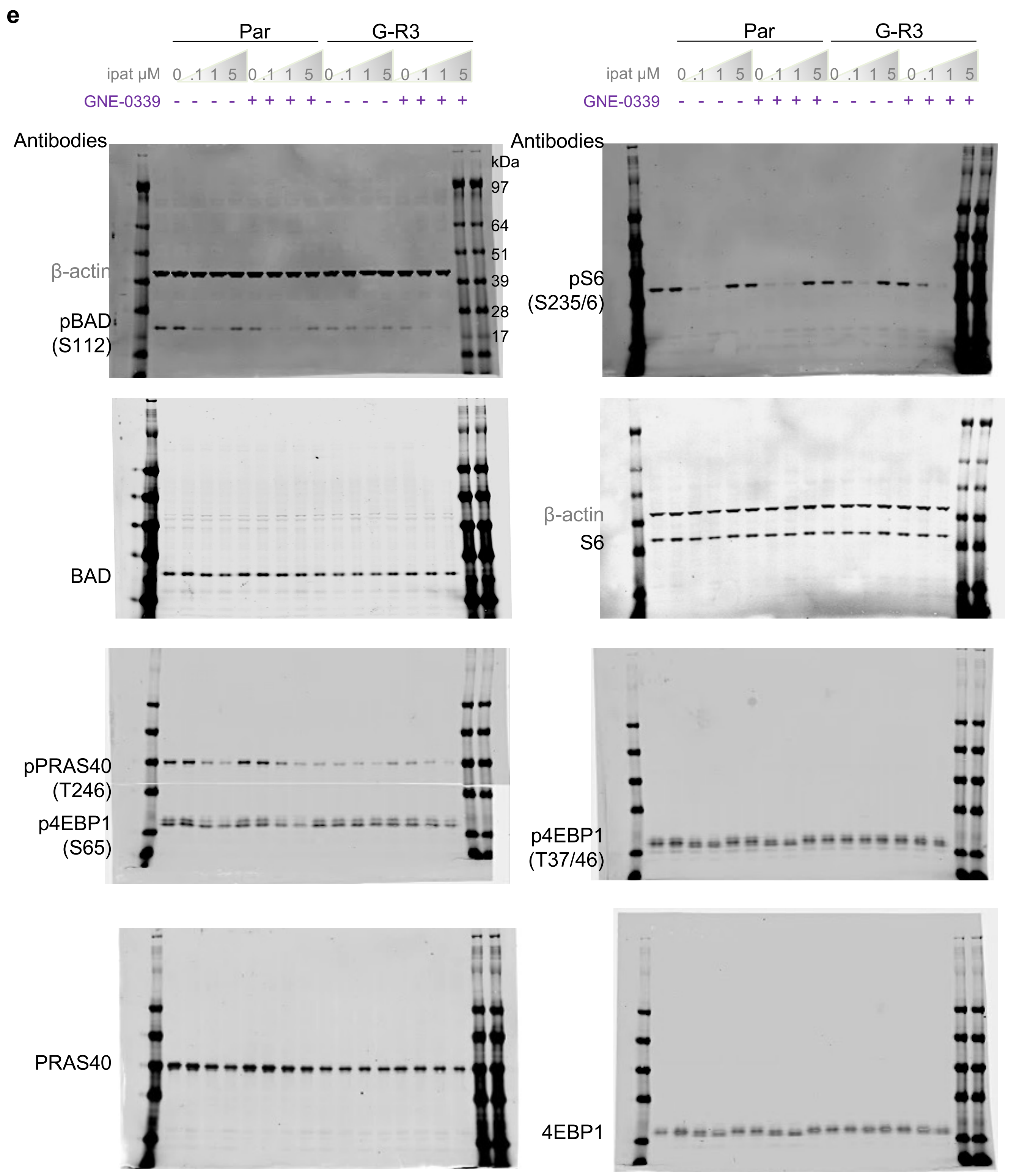
e
Par
G-R3
ipat µM 0 .1 1 5 0 .1 1 5 0 .1 1 5 0 .1 1 5
GNE-0339 - - - - + + + + - - - - + + + +
Par
G-R3
ipat µM 0 .1 1 5 0 .1 1 5 0 .1 1 5 0 .1 1 5
GNE-0339 - - - - + + + + - - - - + + + +
Antibodies
kDa
97
64
51
39
28
17
β-actin
pBAD (S112)
Antibodies
pS6 (S235/6)
BAD
β-actin
S6
pPRAS40 (T246)
p4EBP1 (S65)
p4EBP1 (T37/46)
PRAS40
4EBP1

Figure 4e

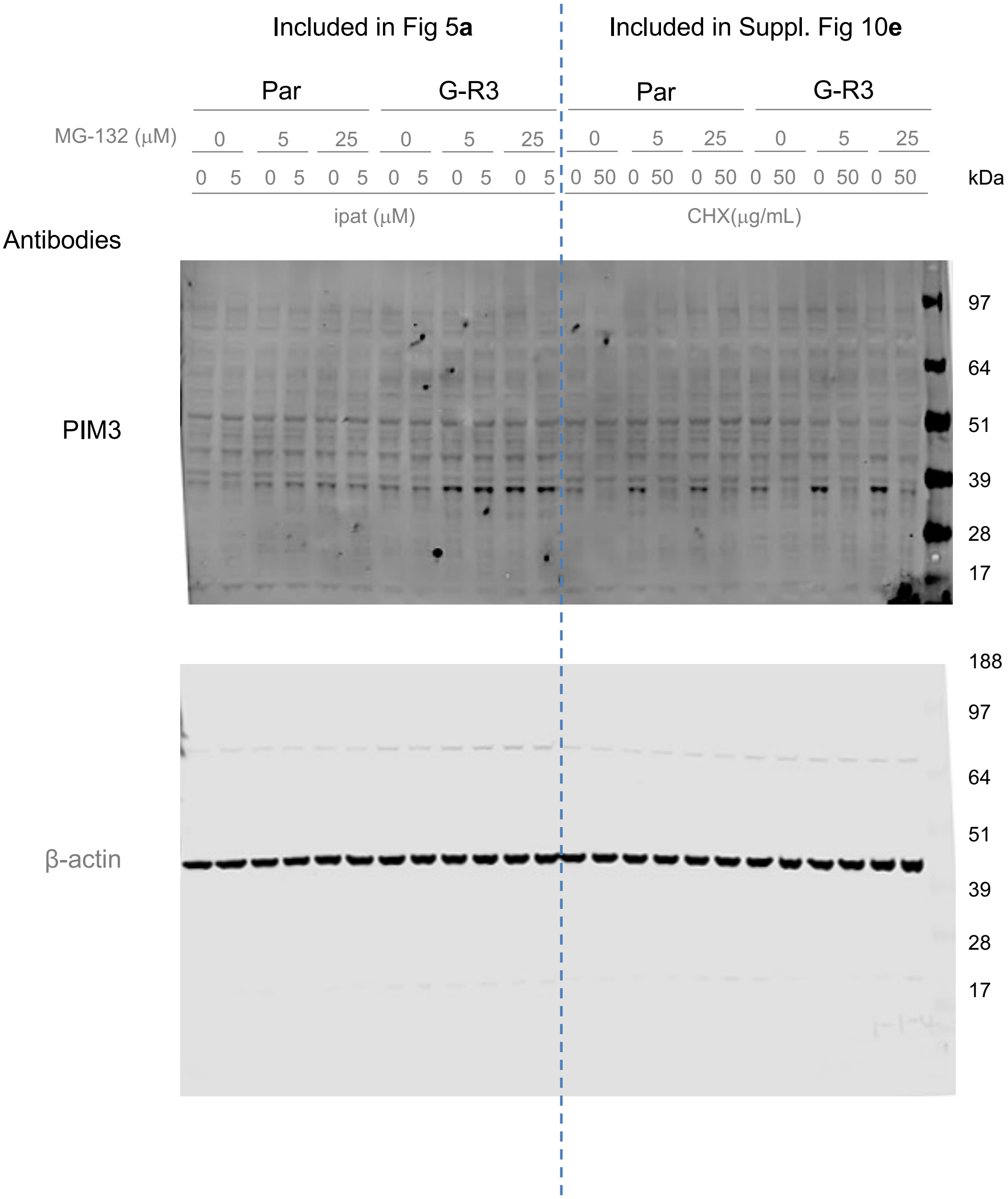

Figure 5a & Supplementary Fig. 10e

b

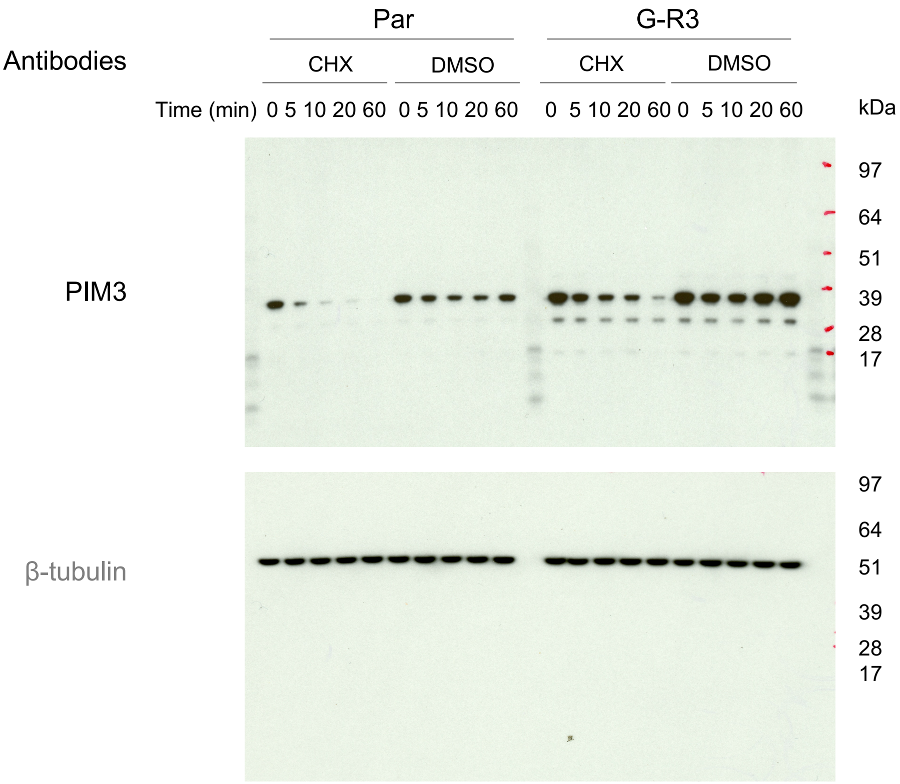
Par
G-R3
Antibodies
CHX
DMSO
CHX
DMSO
Time (min) 0 5 10 20 60 0 5 10 20 60 0 5 10 20 60 0 5 10 20 60
kDa
PIM3
97
64
51
39
28
17
β-tubulin
97
64
51
39
28
17

Figure 5b

c

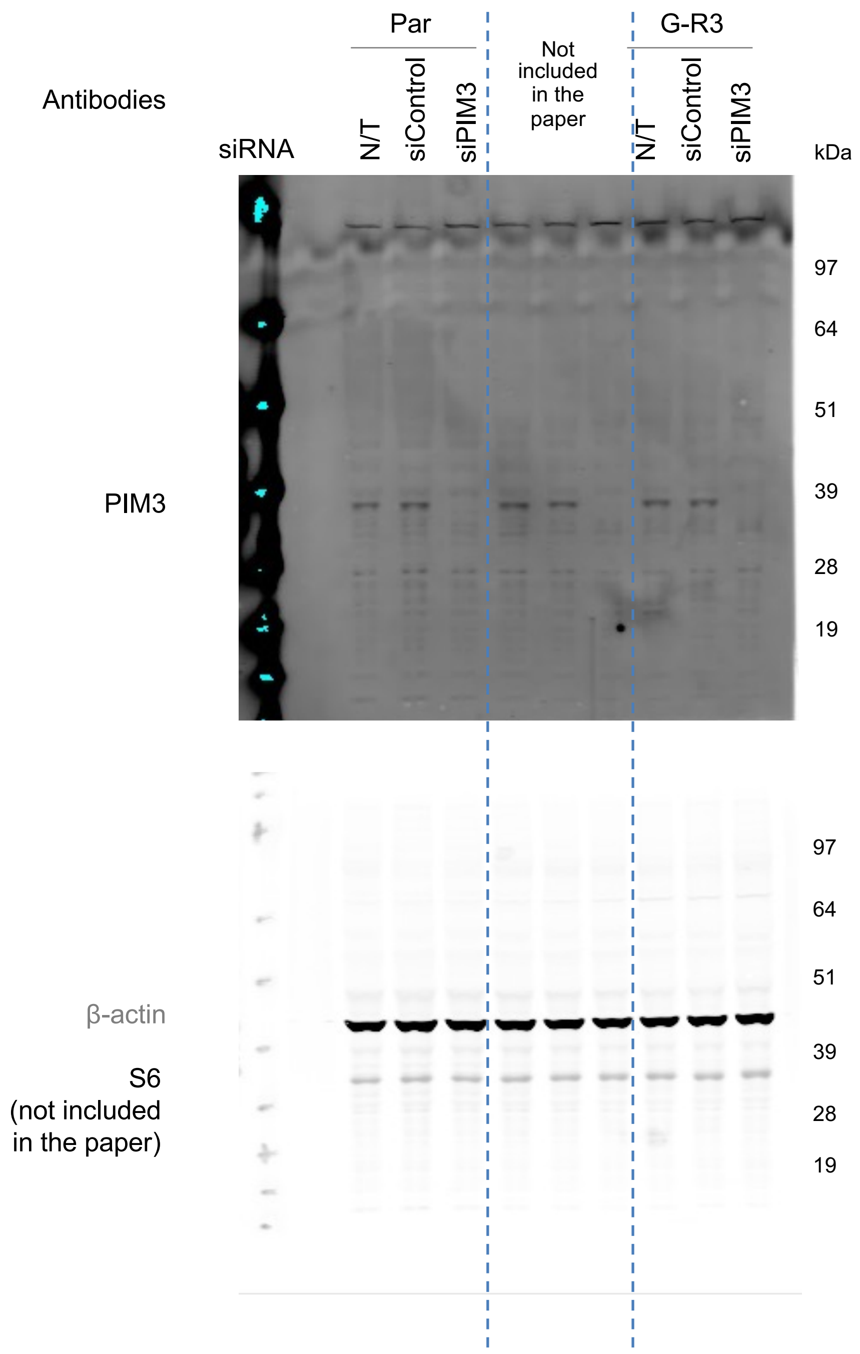
Par
G-R3
Not included in the paper
Antibodies
siRNA
N/T
siControl
siPIM3
N/T
siControl
siPIM3
kDa
97
64
51
39
28
19
PIM3
97
64
51
39
28
19
β-actin
S6 (not included in the paper)

Figure 5c

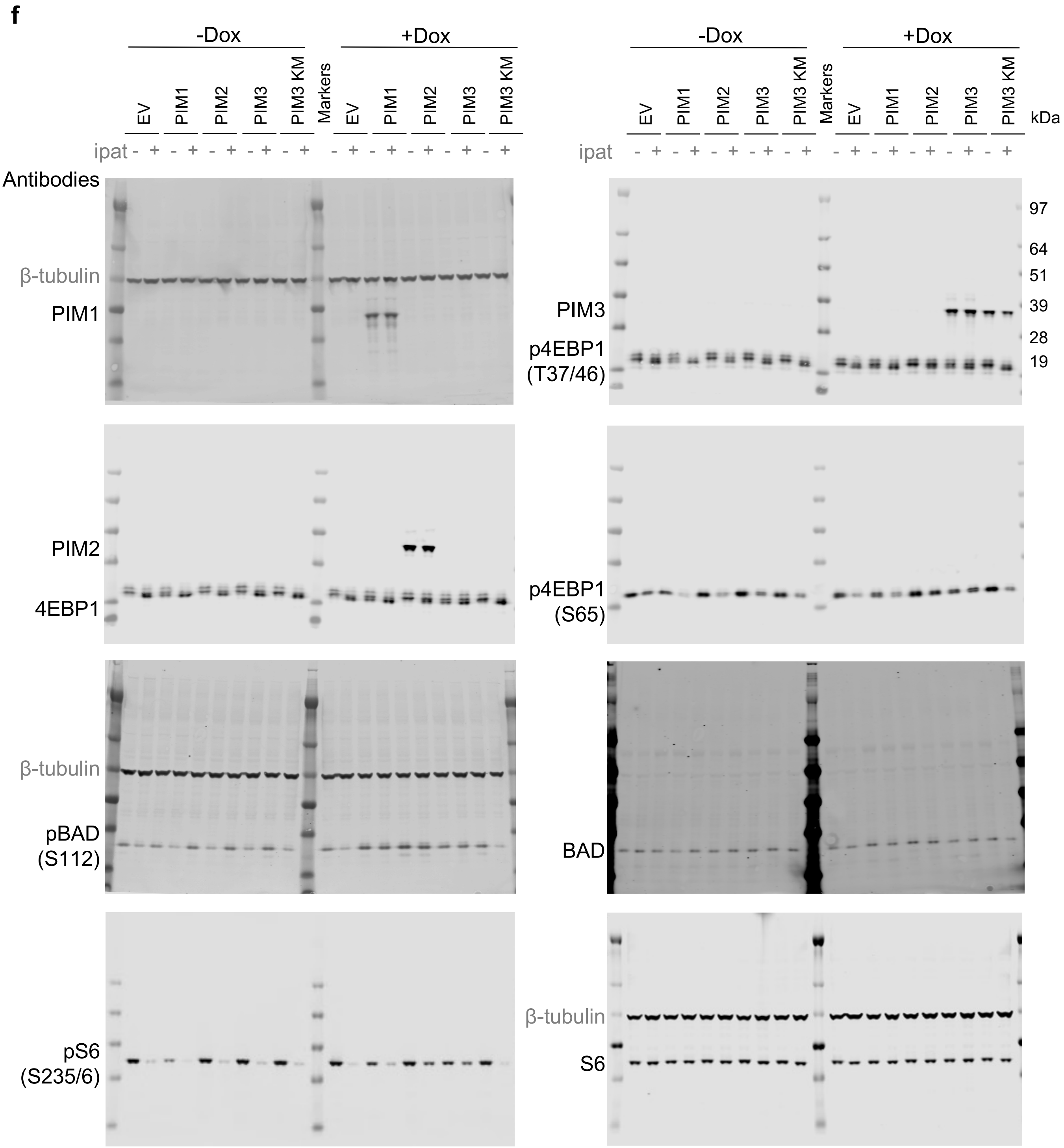
f
-Dox
+Dox
EV
PIM1
PIM2
PIM3
PIM3 KM
Markers
kDa
ipat
Antibodies
β-tubulin
PIM1
PIM3
p4EBP1 (T37/46)
97
64
51
39
28
19
PIM2
4EBP1
p4EBP1 (S65)
β-tubulin
pBAD (S112)
BAD
pS6 (S235/6)
β-tubulin
S6

Figure 5f

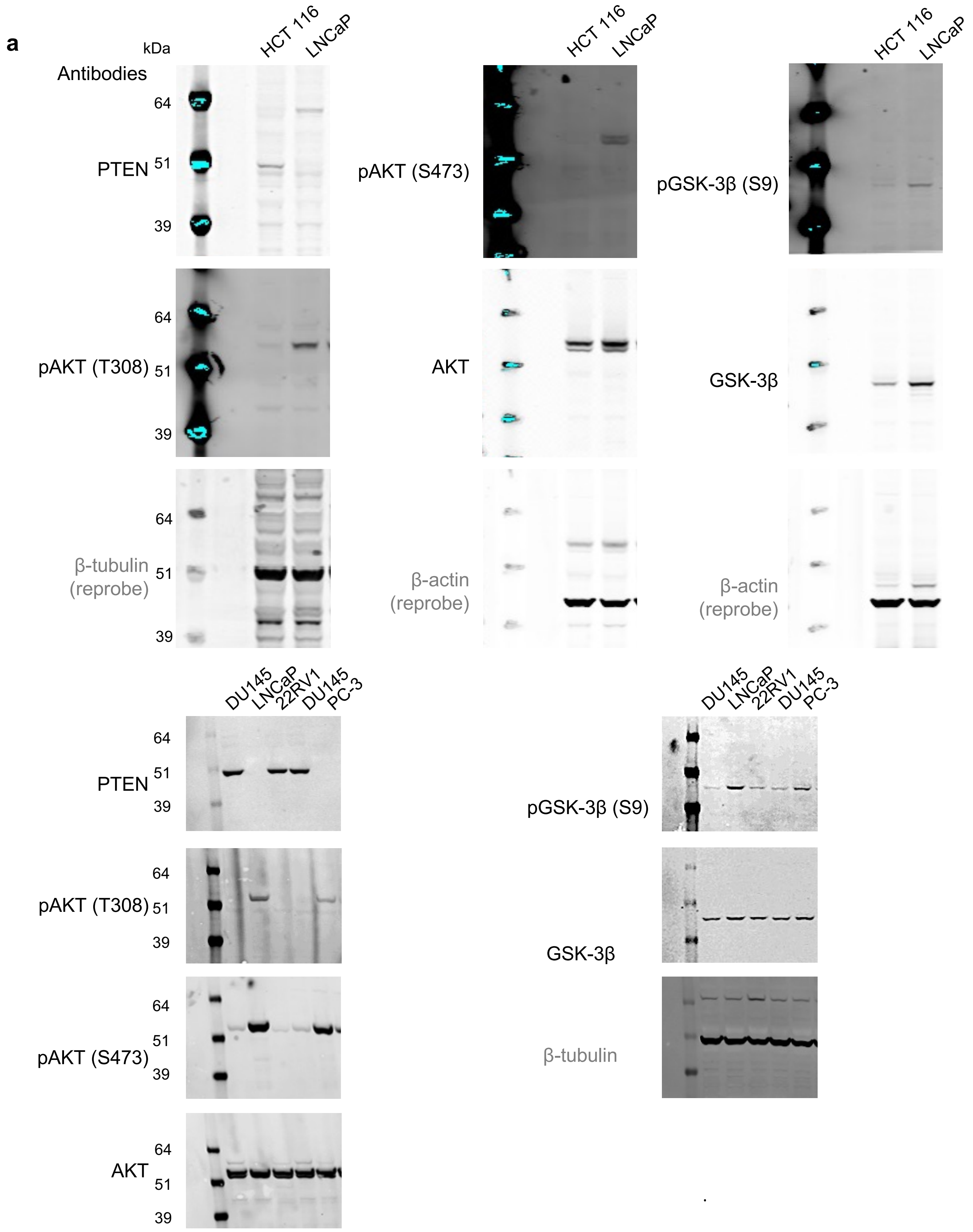

Supplementary Fig. 1a

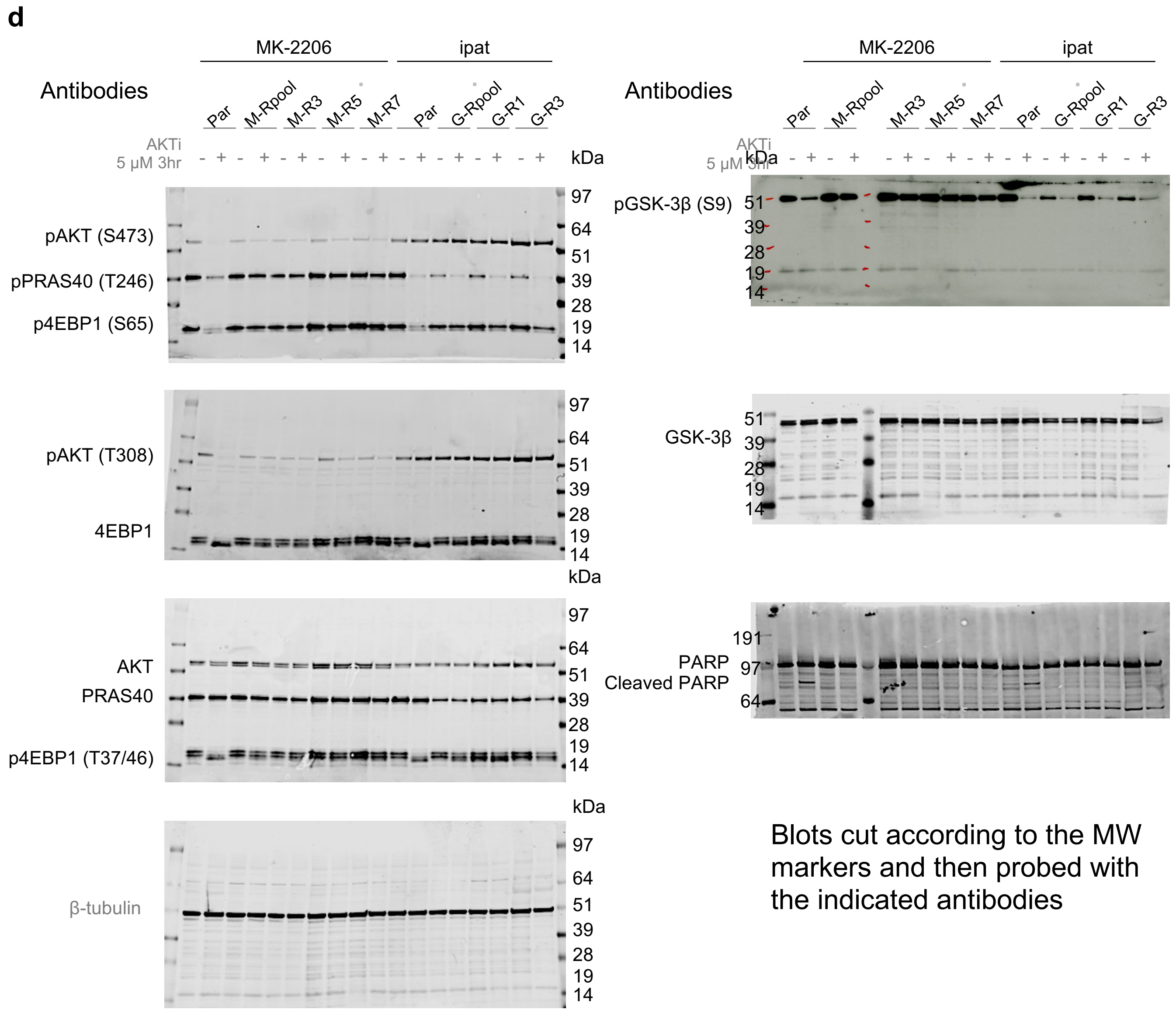

Supplementary Fig. 1d

e

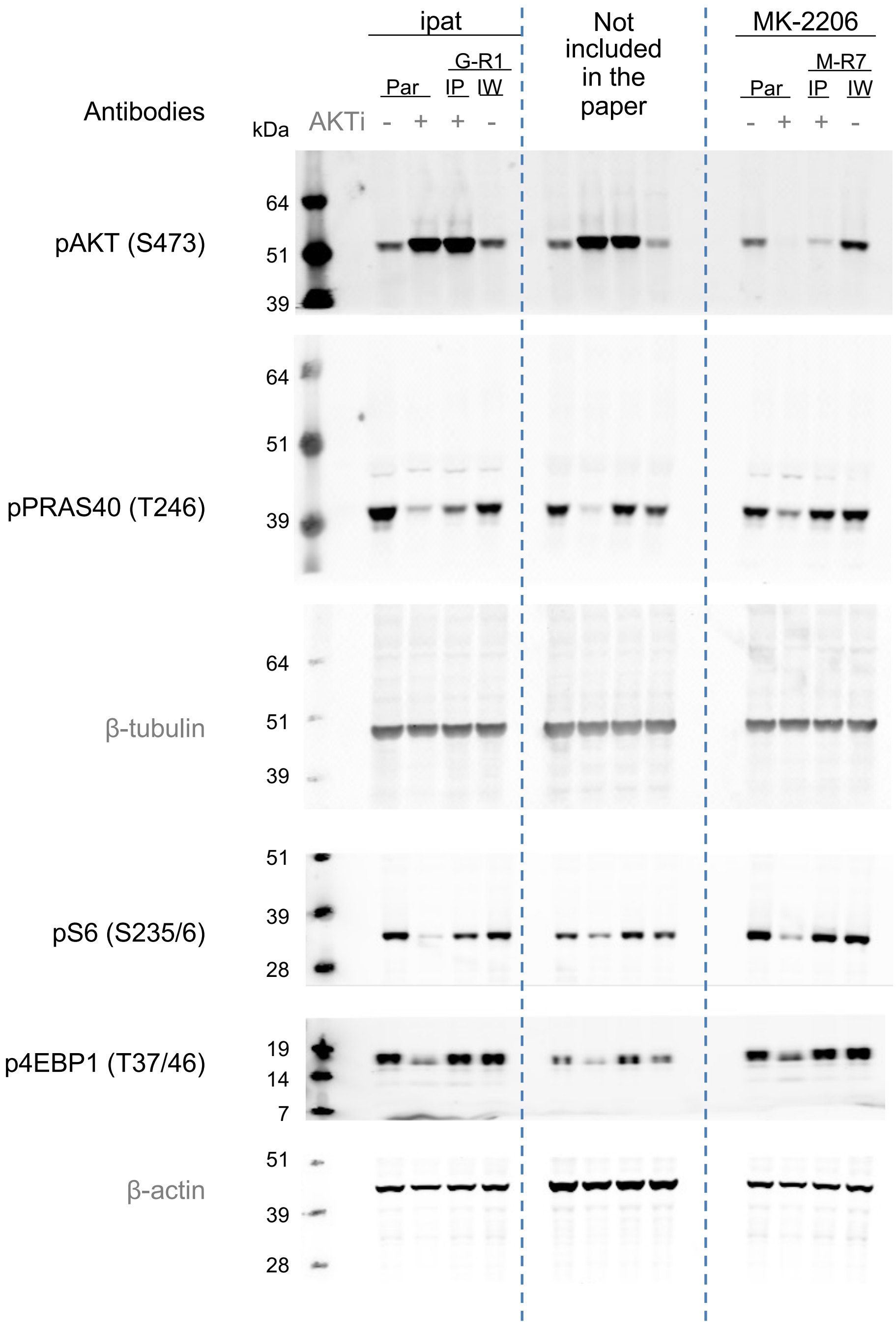

Blots cut according to the MW markers and then probed with the indicated antibodies

Supplementary Fig. 1e

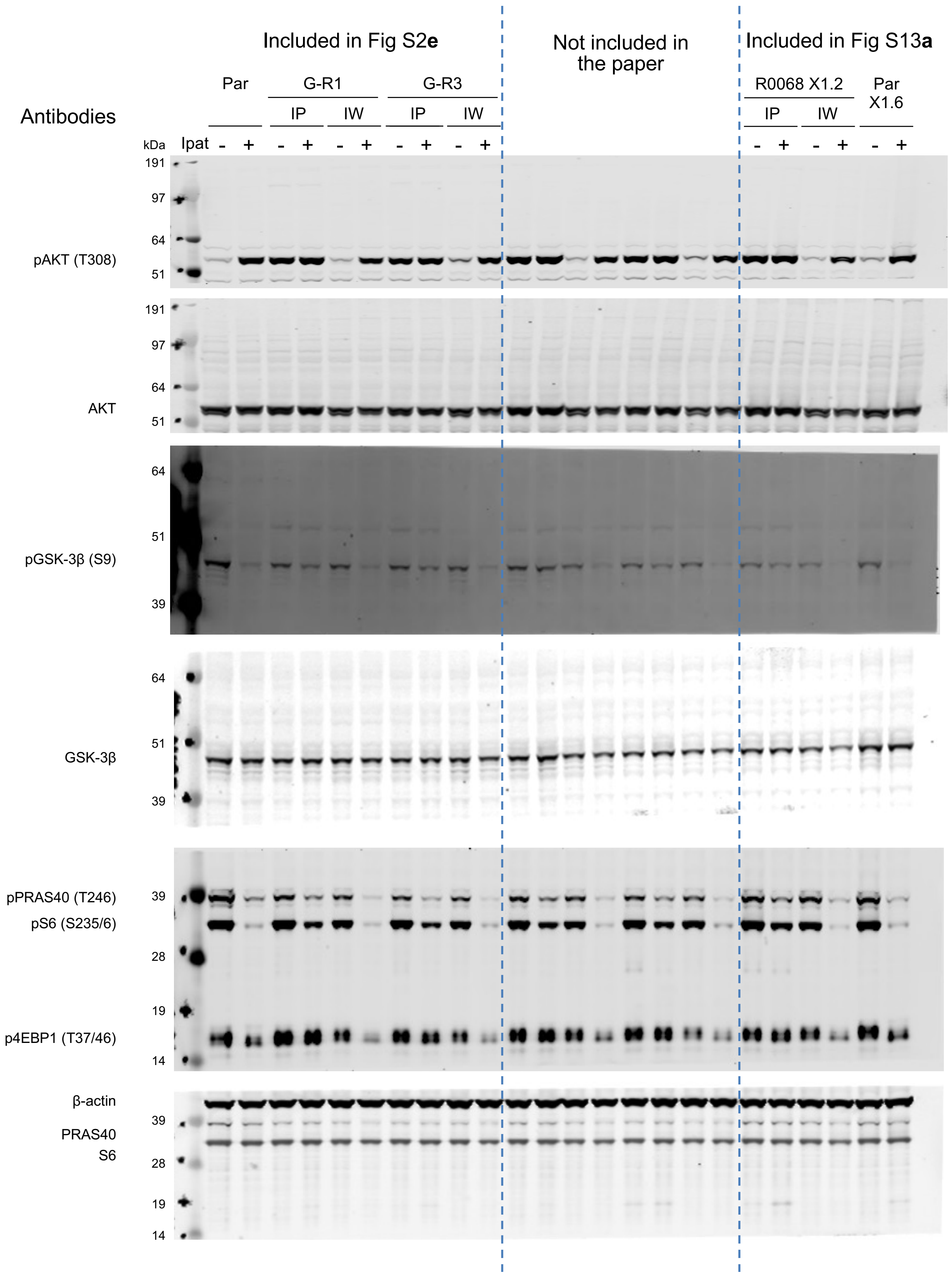

Blots cut according to the MW markers and then probed with the indicated antibodies

Supplementary Fig. 2e & 13a

c

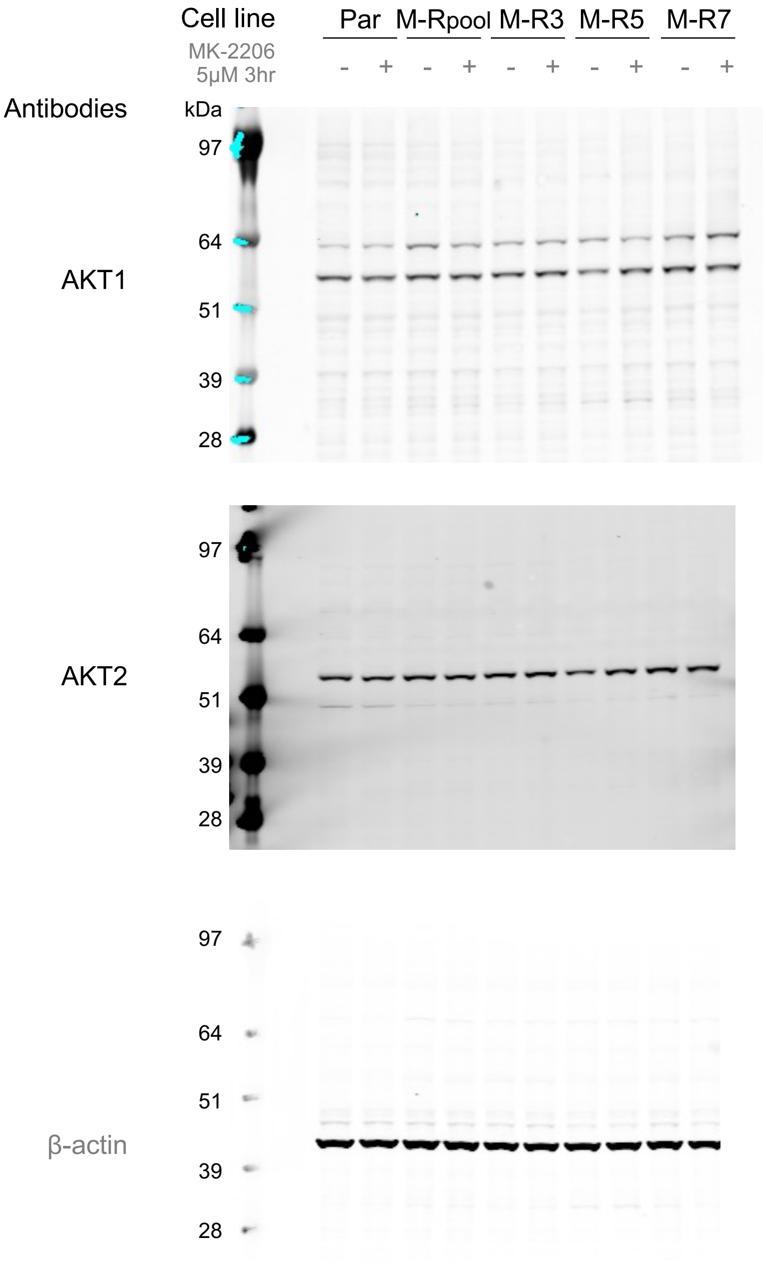

Supplementary Fig. 3c

e

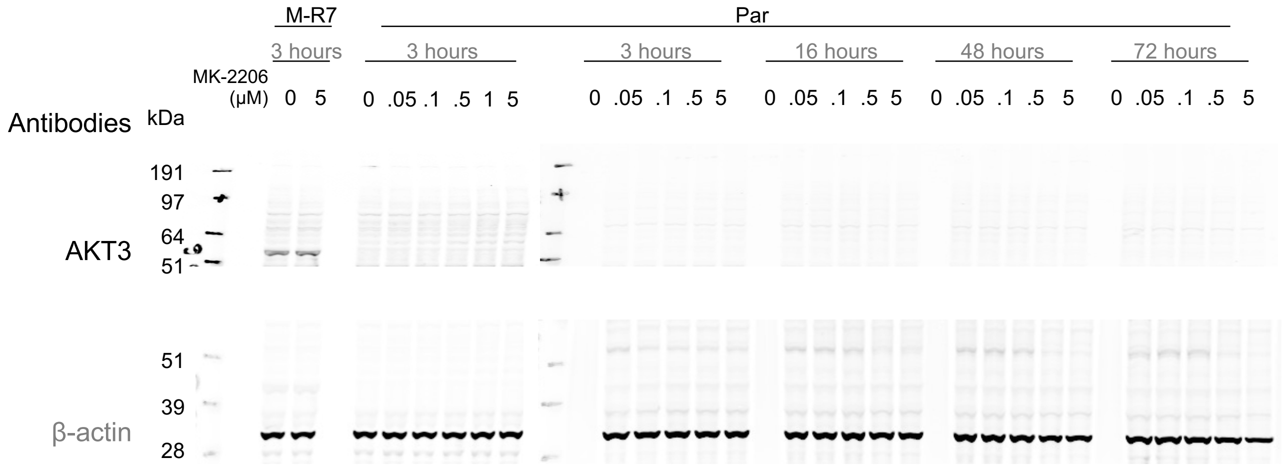

f

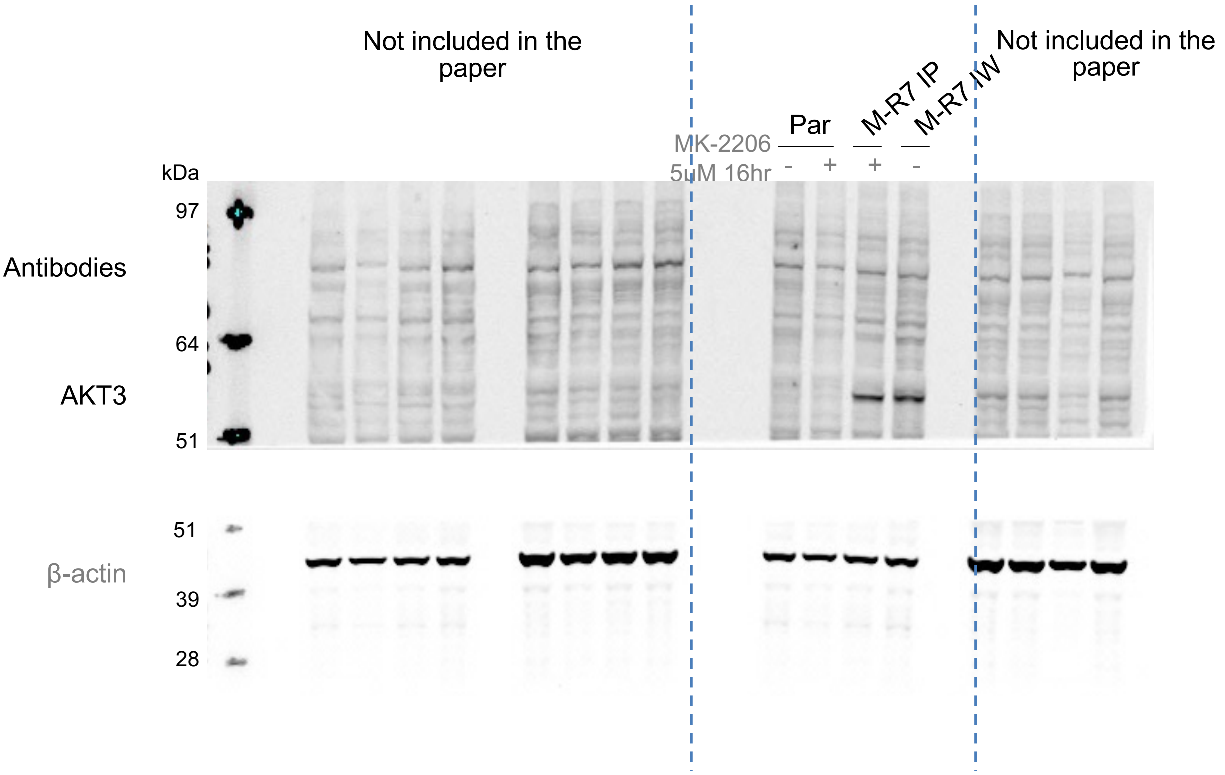

Supplementary Fig. 3e

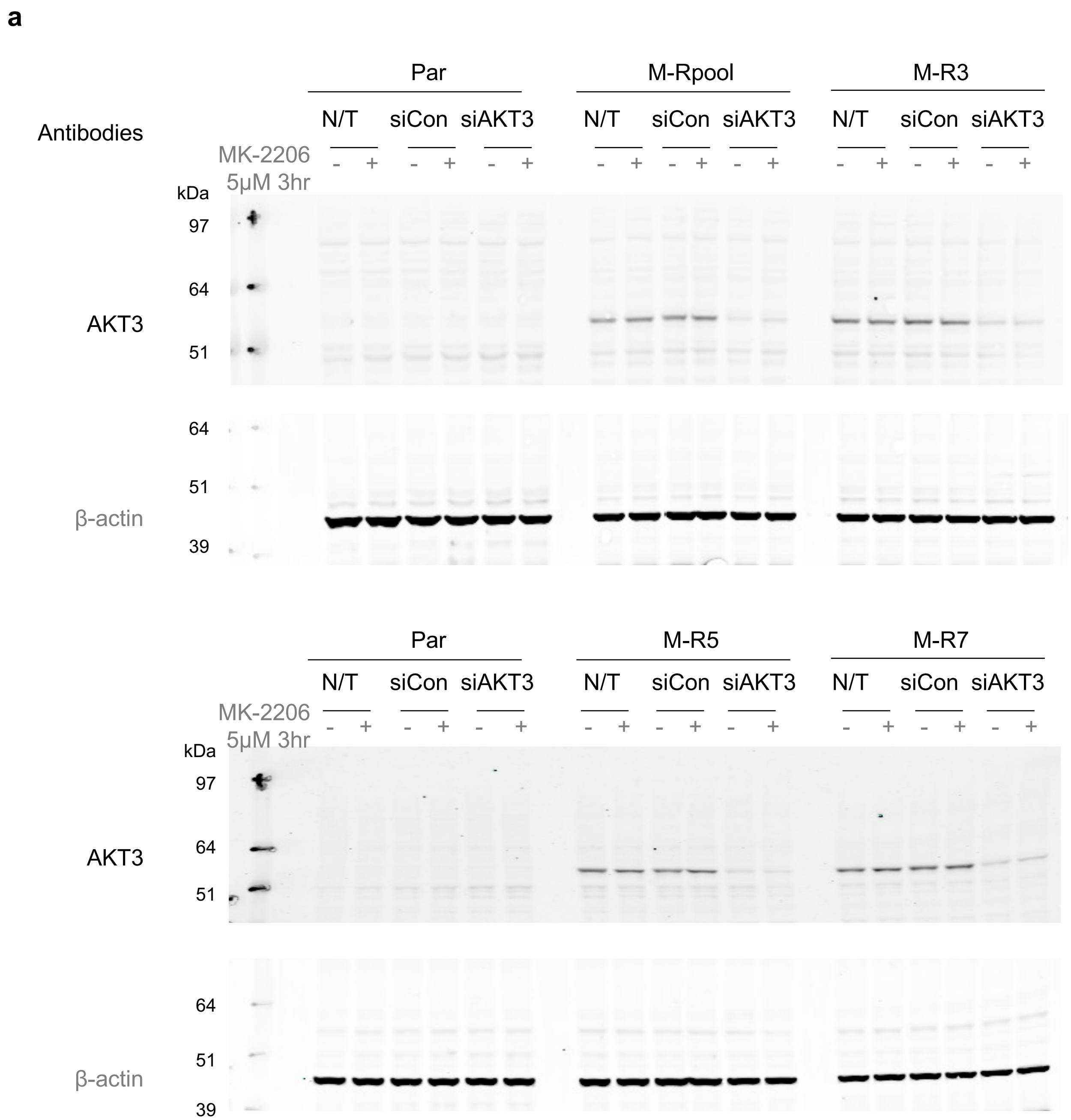

Supplementary Fig. 4a

c

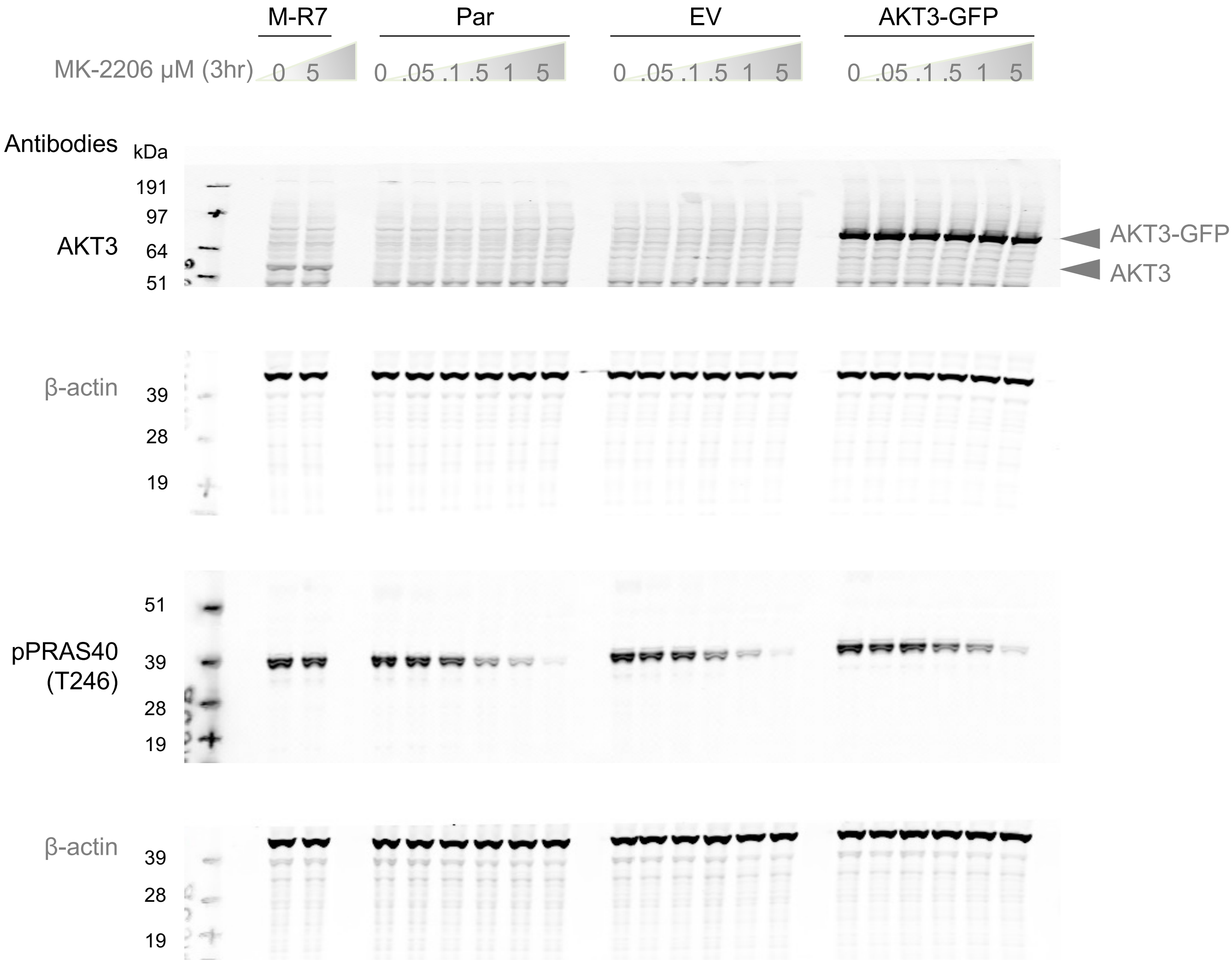

Blots cut according to the MW markers and then probed with the indicated antibodies

Supplementary Fig. 4c

c

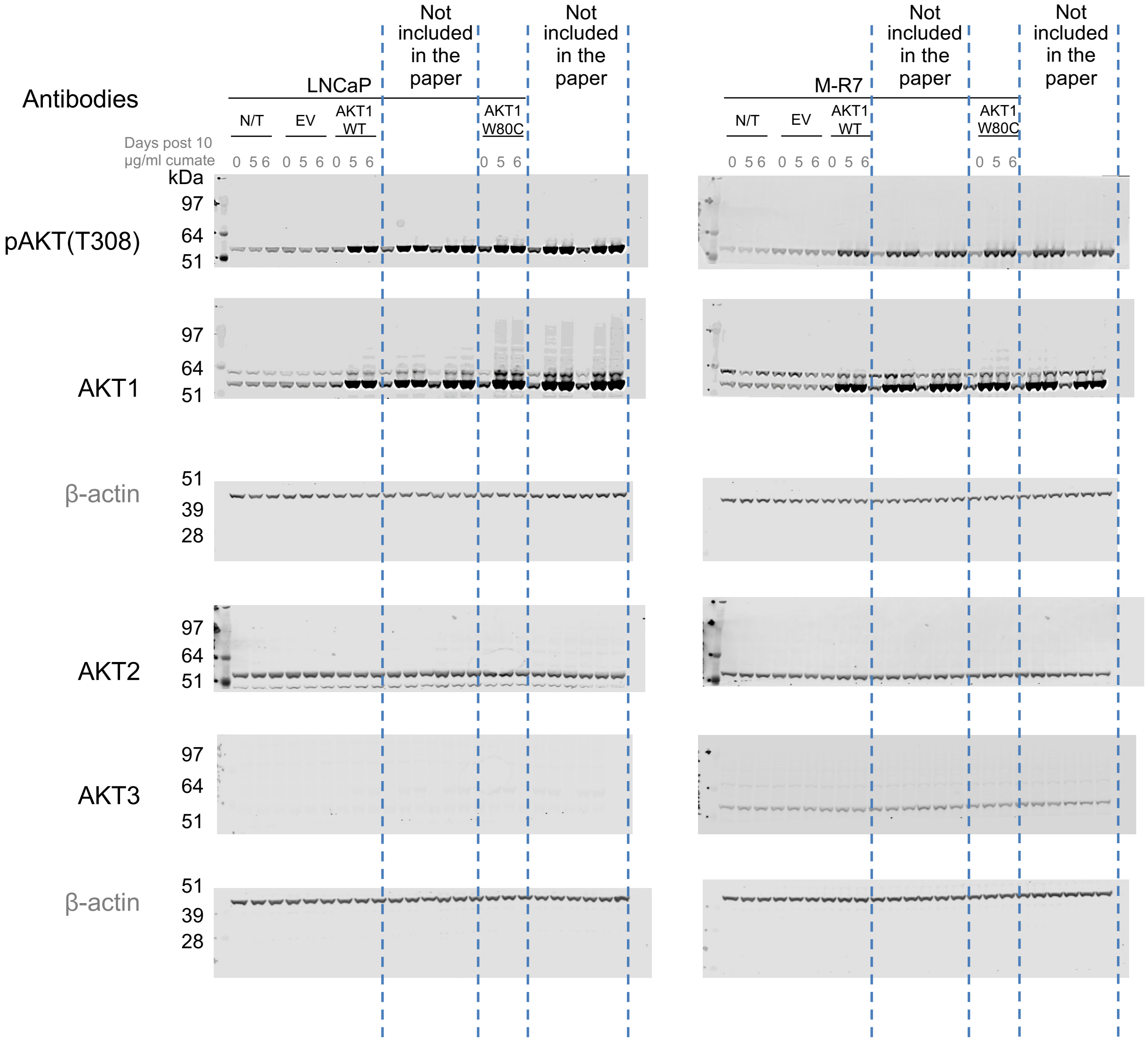

Blots cut according to the MW markers and then probed with the indicated antibodies

Supplementary Fig. 5c

d

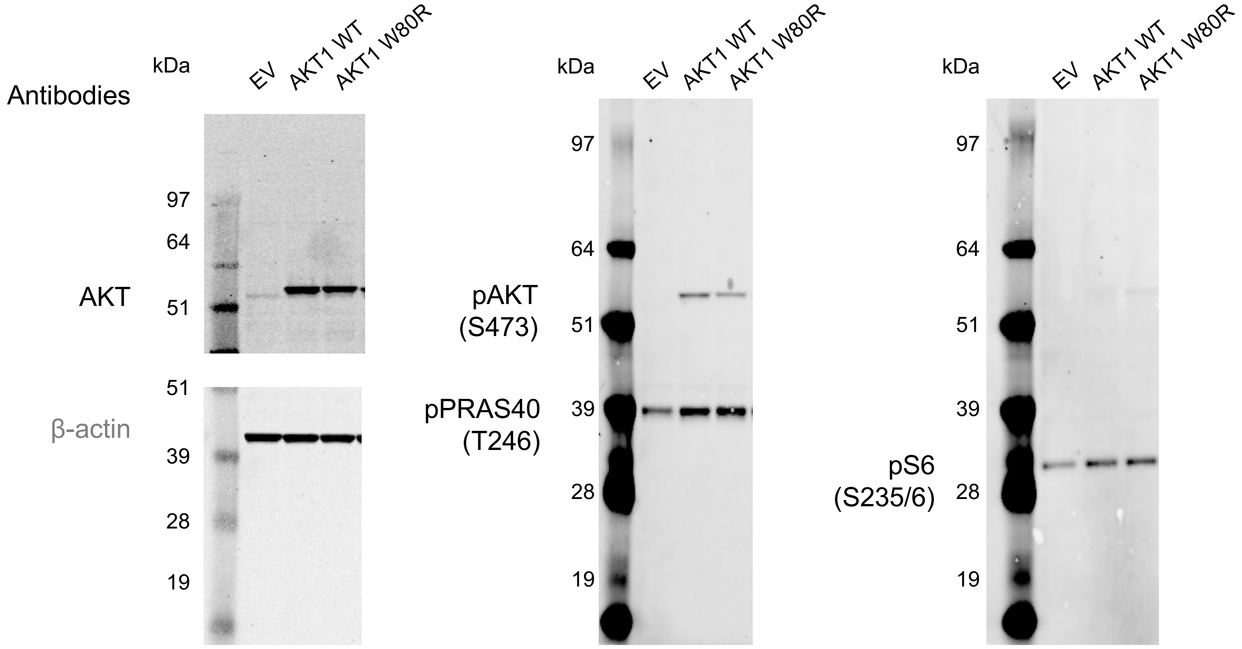

Blots cut according to the MW markers and then probed with the indicated antibodies

f

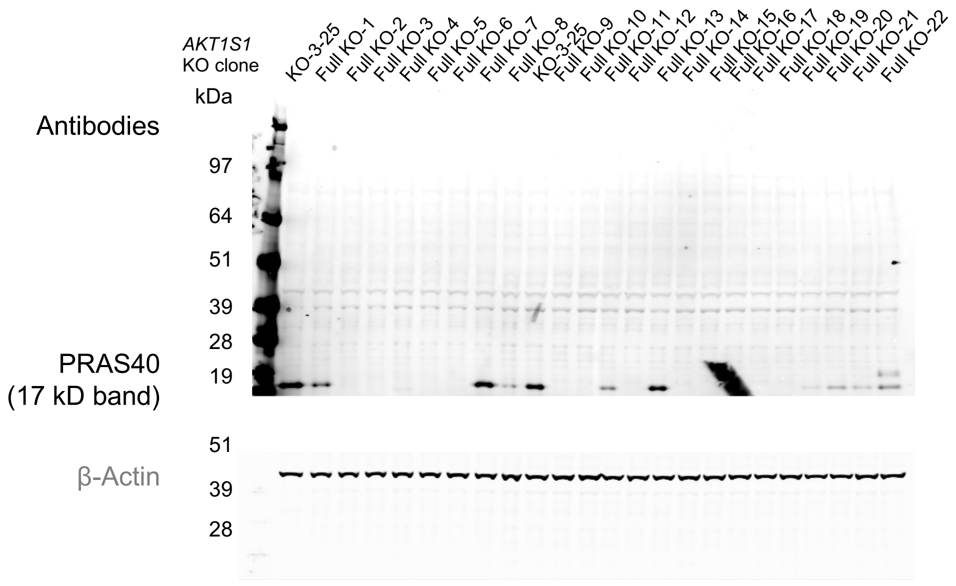

Blots cut according to the MW markers and then probed with the indicated antibodies

Supplementary Fig. 6f

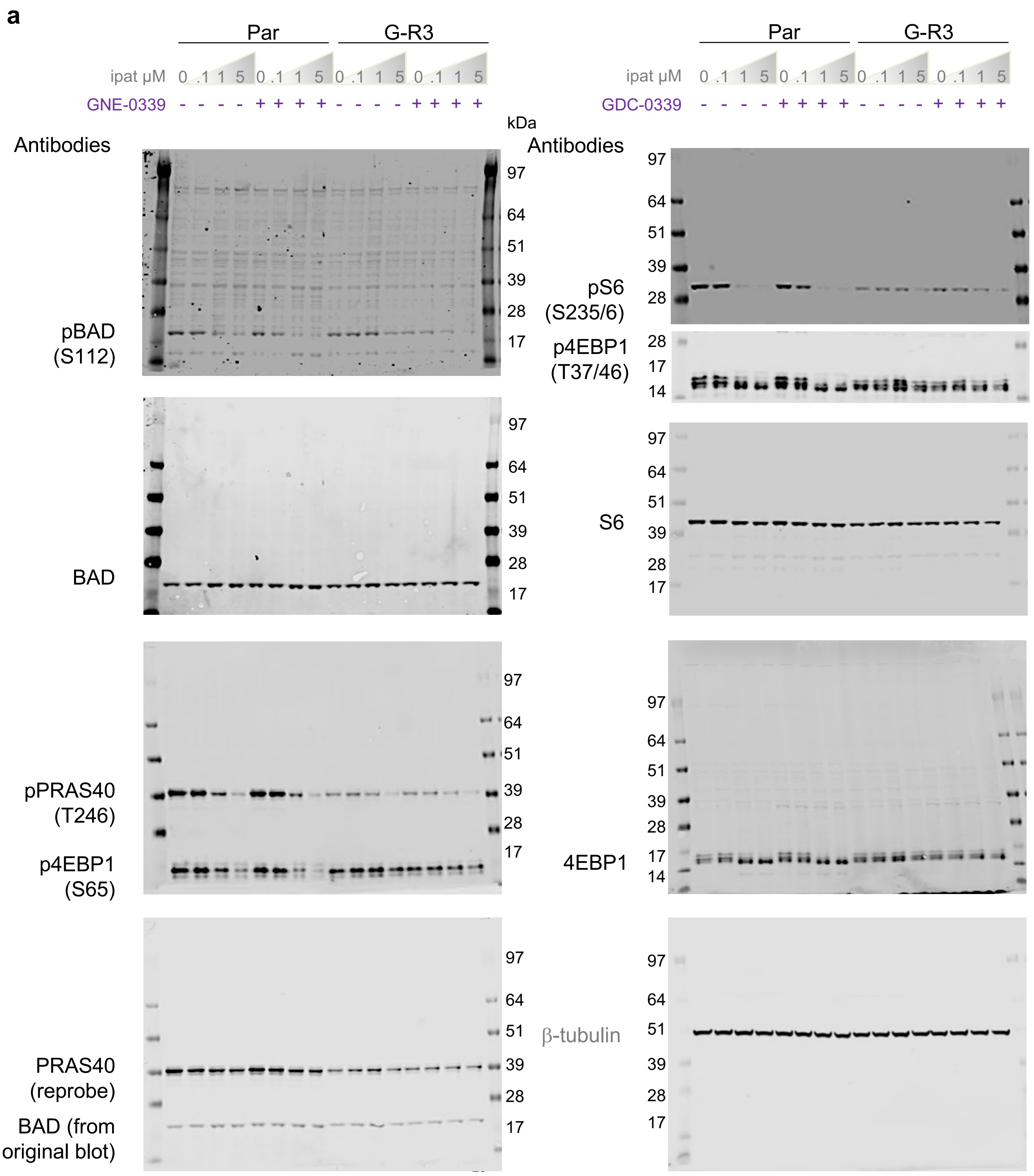

Supplementary Fig. 8a

b

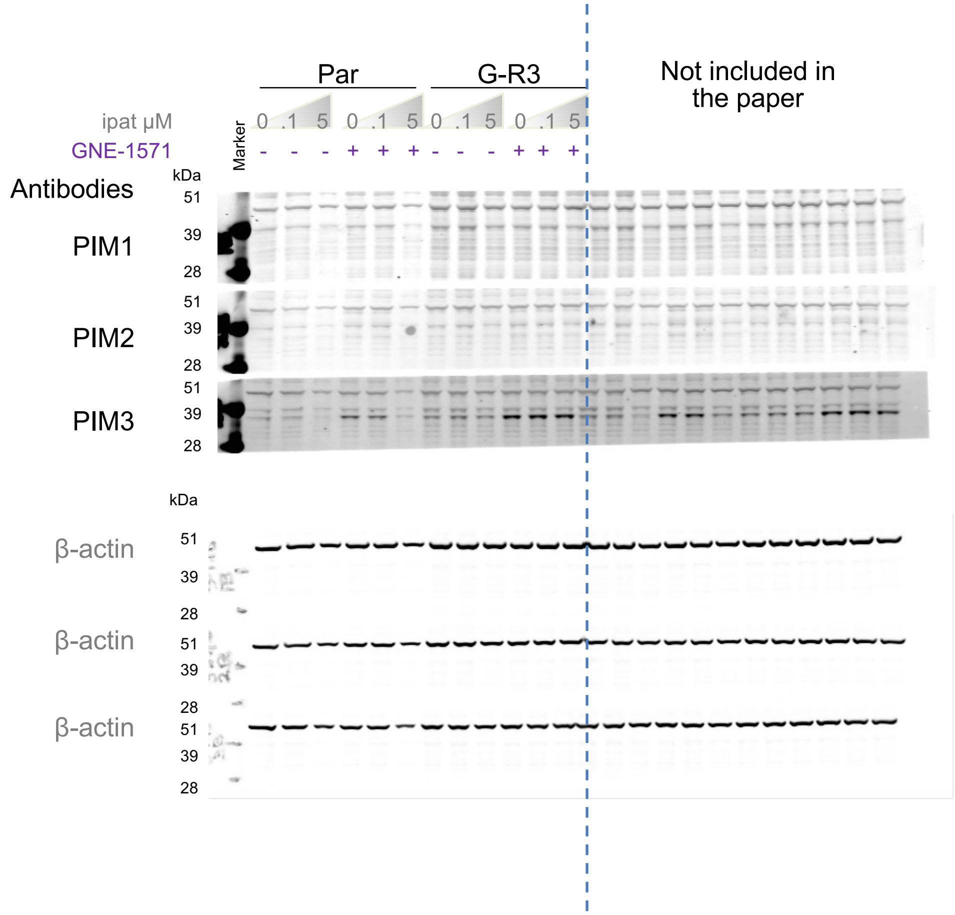

Blots cut according to the MW markers and then probed with the indicated antibodies

Supplementary Fig. 10b

d

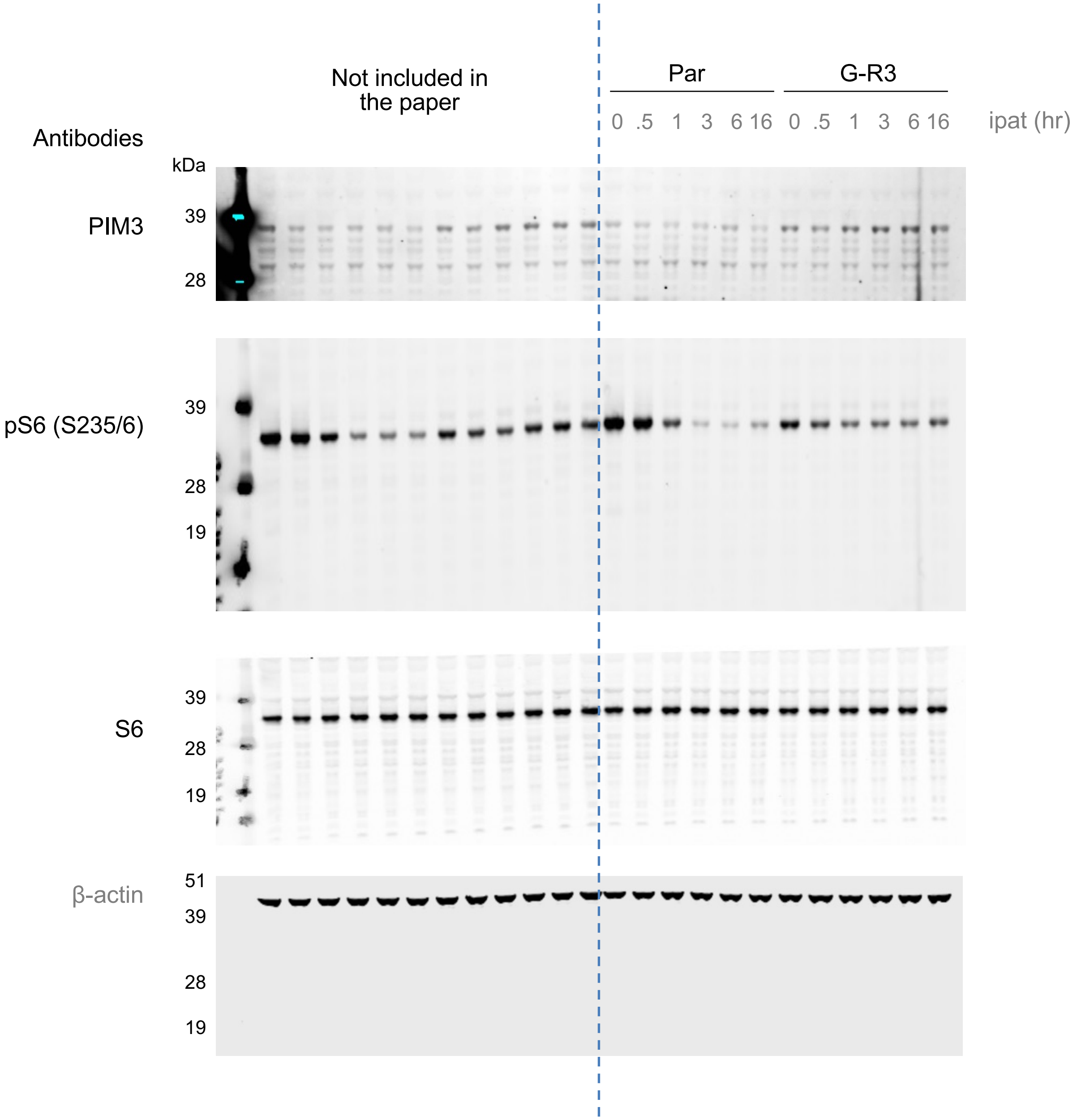

Blots cut according to the MW markers and then probed with the indicated antibodies

Supplementary Fig. 10d

f

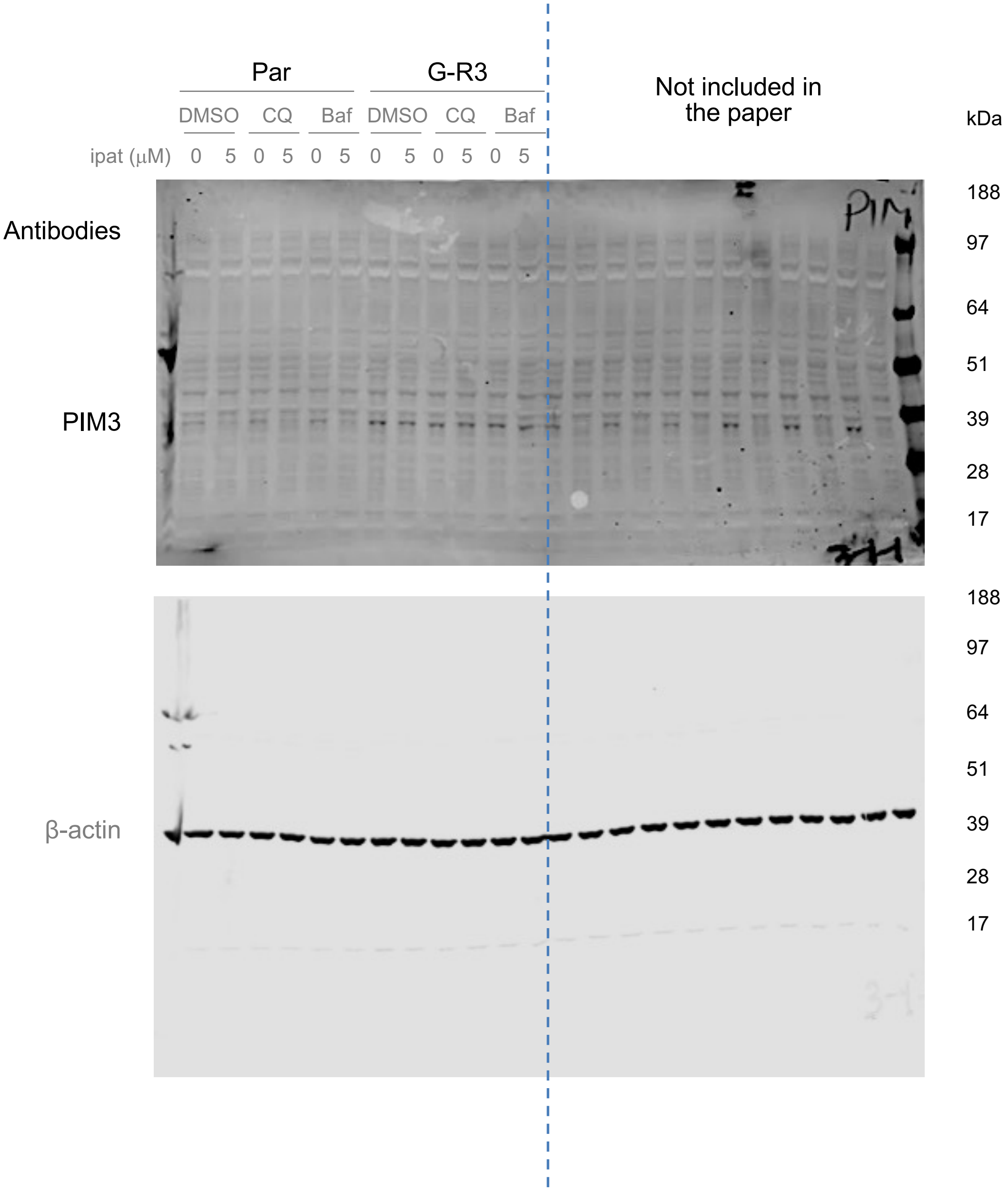

Supplementary Fig. 10f

b

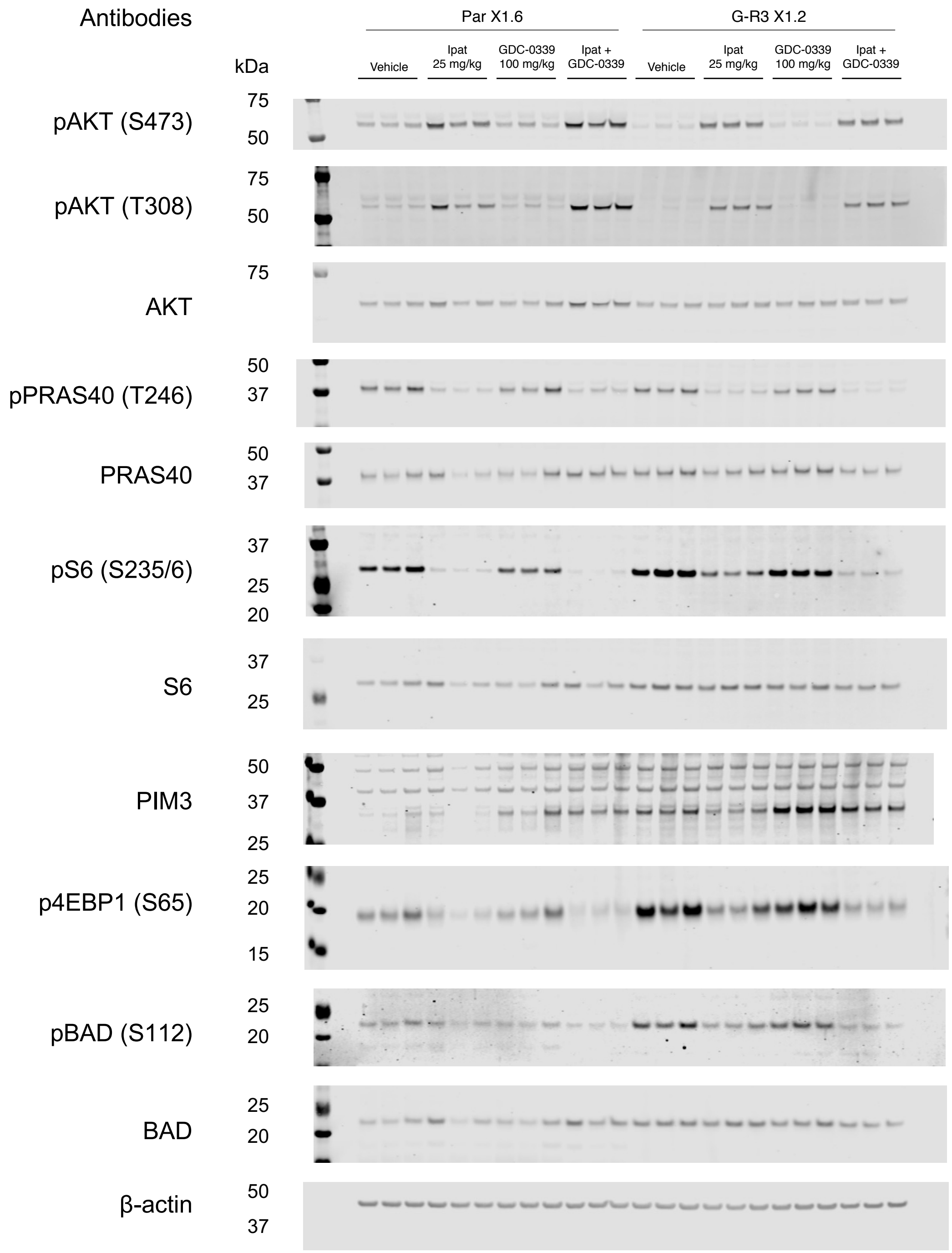

Blots cut according to the MW markers and then probed with the indicated antibodies

Supplementary Fig. 12b

b

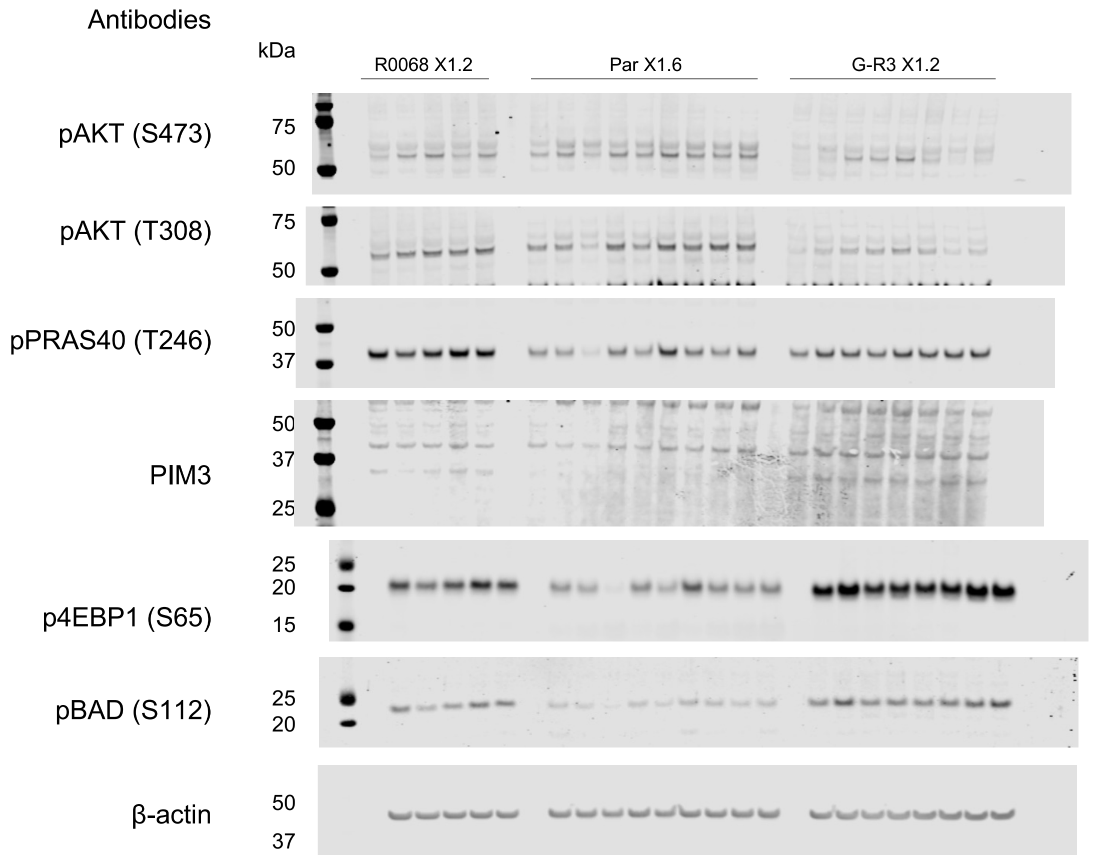

Blots cut according to the MW markers and then probed with the indicated antibodies

Supplementary Fig. 13b

e

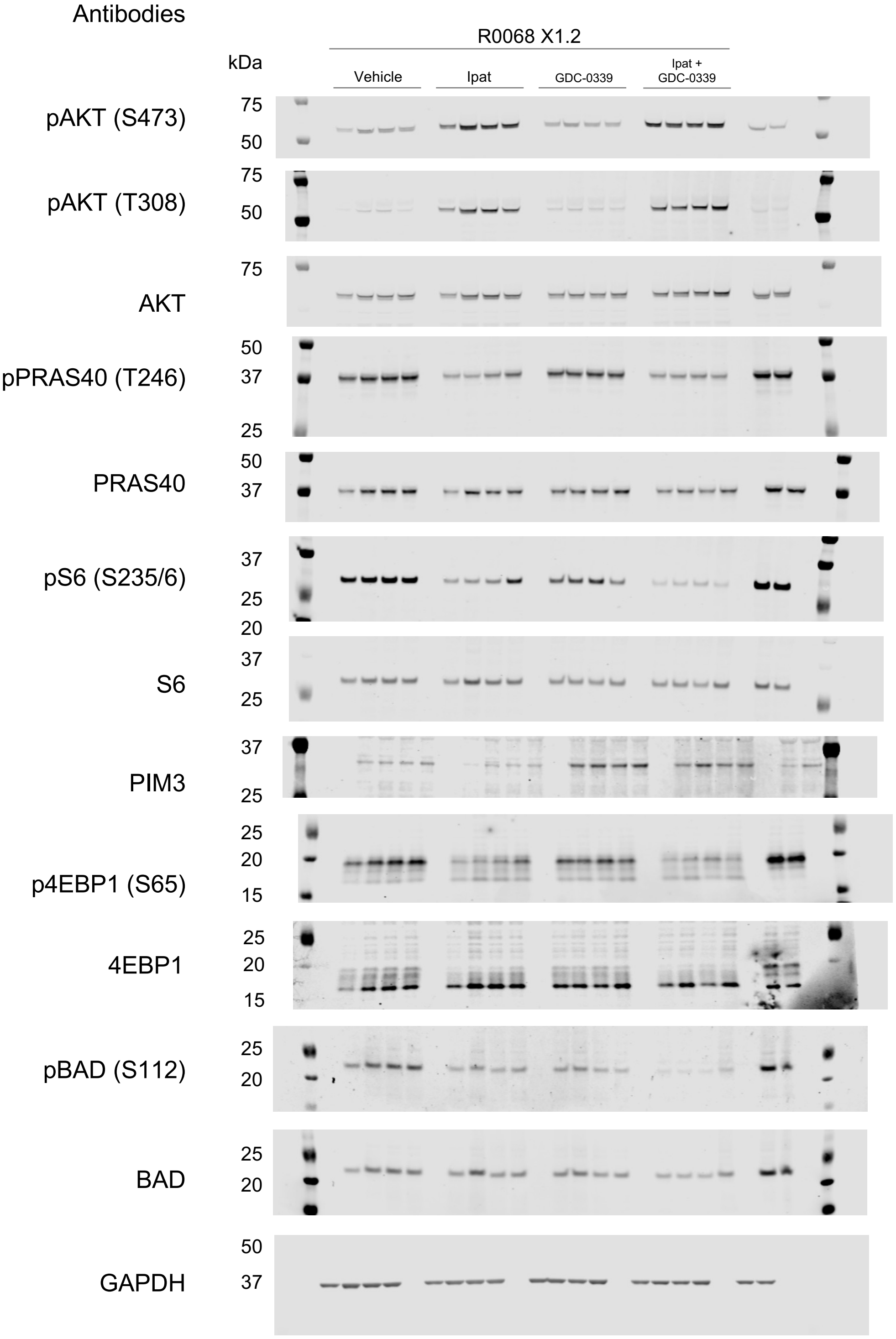

Blots cut according to the MW markers and then probed with the indicated antibodies

Supplementary Fig. 13e
