# Supplementary material for: Distinct resistance mechanisms arise to allosteric vs. ATP-competitive AKT inhibitors
Source: Nat Commun. 2022 Apr 19;13:2057. doi: 10.1038/s41467-022-29655-0 (PMC9019088; doi:10.1038/s41467-022-29655-0)
Supplement: Supplementary file 2 — Inventory of Supporting Information [file 41467_2022_29655_MOESM2_ESM.docx]

**Inventory of Supporting Information:**

1. **Supplementary Information (PDF file) with the following content:**

Supplementary Fig. 1, related to Fig. 1. Overview of LNCaP Par and AKTi-R cell characteristics

Supplementary Fig. 2, related to Fig. 1. Partial reversion of AKTi resistance is observed in G-R but not M-R cells

Supplementary Fig. 3, related to Fig. 2. Identification of genes specifically upregulated in M-R vs. Par -/+ MK-2206 and AKT isoform expression and copy number in LNCaP and M-R cells

Supplementary Fig. 4, related to Fig. 2. The impact of AKT3 knockdown or overexpression on Par vs. additional M-R cell lines

Supplementary Fig. 5, related to Fig. 3. The AKT1 W80C mutation and its assessment using a cumate-inducible piggyBac system

Supplementary Fig. 6, related to Fig. 4. *AKT1S1*/PRAS40 Q178* mutation in G-R cells and effect of CRISPR/Cas9-mediated KO of *AKT1S1* or knock-in of the *AKT1S1* Q178* mutation in LNCaP cells

Supplementary Fig. 7, related to Fig. 4. Design of the chemical genetics screen and the identification of PIM kinase inhibitors as screen hits.

Supplementary Fig. 8, related to Fig. 4. Immunoblot analysis of the combination effects between ipat and PIMi in parental and G-R3 cells.

Supplementary Fig. 9, related to Fig. 4. Combination effects between AKTi and PIMi in prostate cancer cell lines.

Supplementary Fig. 10, related to Fig. 5. Characterization of PIM1, 2, 3 expression and inducible PIM expression in LNCaP cells.

Supplementary Fig. 11, related to Fig. 6. Combined treatment with a PIMi overcomes resistance to ipat *in vivo* in an ipat-resistant model established *in vitro*.

Supplementary Fig. 12, related to Fig. 6. Biomarker analysis of the tumors.

Supplementary Fig. 13, related to Fig. 6. Combined treatment with a PIMi overcomes resistance to ipat *in vivo* in an ipat-resistant model established *in vivo*.

Appendix: Raw Immunoblot Images.

1. **Supplementary Data Files (5 excel files):**

Supplementary Data 1, related to Fig. 2. Gene expression changes in AKTi-R cells from RNA-seq analysis and alignment to GRCh37 (hg19)

Differential expression was determined by comparison of RPKM values in individual or multiple AKTi-R clones with that of Par cells. For comparisons with multiple clones, the mean RPKM value of the AKTi-R clones was determined and compared with that of Par cells. Expression was considered to be altered when associated with p ≤ 0.05 and log2 fold change ≥ 1. Various comparisons between individual or multiple G-R or M-R clones (Group 2) and Par (Group 1) are depicted in individual worksheets as indicated by sheet titles.

Supplementary Data 2, related to Fig. 2. Gene copy number in AKTi-R and Par cells from SNP array analysis

Log2 (total gene copy number/sample ploidy) values are depicted. Values >0.3 indicate copy number gain and values <0.3 indicate copy number loss. Values <-0.5 or >0.5 are highlighted.

Supplementary Data 3, related to Fig. 3. Genetic alterations in AKTi-R cells from exome-seq analysis

Genetic alterations detected in the various AKTi-R pool cell lines or individual clones are depicted in individual worksheets as indicated by sheet titles.

Supplementary Data 4, related to Fig. 3. AKT1 W80R prevalence in various cancer indications

Cancer genomics studies in which AKT1 W80 alterations were detected in patients were first identified using cBioPortal (<http://www.cbioportal.org/index.do?session_id=5b5e1288498eb8b3d5672636>). All AKT1 mutation information reported in those selected studies was then retrieved. The frequency of each AKT1 mutation detected within the same indication was calculated from these studies (# of patients harboring a specific AKT1 mutation/total # patients with that indication within the data set). TCGA: The Cancer Genome Atlas, [https://portal.gdc.cancer.gov](https://portal.gdc.cancer.gov/). METABRIC: Molecular Taxonomy of Breast Cancer International Consortium Nature 2012 & Nat Commun 2016, Pierra et al., 2016 <https://www.ncbi.nlm.nih.gov/pubmed/27161491>; MSK-IMPACT: Memorial Sloan Kettering Cancer Center's Integrated Mutation Profiling of Actionable Cancer Targets (MSKCC, Nat Med 2017). AKT1 mutation data from the three studies are depicted in individual worksheets as indicated by sheet titles. Rows containing information associated with the AKT1 W80R mutation are highlighted in yellow.

Supplementary Data 5, related to Fig. 4. Chemical genetics screening results in Par, G-R1, and G-R3.

General information on the individual inhibitors included in the chemical genetics screen such as internal identification number, target name, and target class are depicted. Additionally, mean viability (MV) and IC_50_ values associated with par, G-R1 and G-R3 cells for each inhibitor are presented; the MV difference and log2 fold change (FC) in IC_50_ values of G-R cells compared with that of parental cells are also depicted.

1. **Source Data File (1 excel file)**
