# Supplementary material for: Distinct resistance mechanisms arise to allosteric vs. ATP-competitive AKT inhibitors
Source: Nat Commun. 2022 Apr 19;13:2057. doi: 10.1038/s41467-022-29655-0 (PMC9019088; doi:10.1038/s41467-022-29655-0)
Supplement: Supplementary file 8 — Reporting Summary [file 41467_2022_29655_MOESM8_ESM.pdf]

nature portfolio

Corresponding author(s): Kui Lin

Last updated by author(s): Feb 28, 2022

Reporting Summary

Nature Portfolio wishes to improve the reproducibility of the work that we publish. This form provides structure for consistency and transparency in reporting. For further information on Nature Portfolio policies, see our Editorial Policies and the Editorial Policy Checklist.

Statistics

For all statistical analyses, confirm that the following items are present in the figure legend, table legend, main text, or Methods section.

n/a	Confirmed	
☐	☒	The exact sample size (*n*) for each experimental group/condition, given as a discrete number and unit of measurement
☐	☒	A statement on whether measurements were taken from distinct samples or whether the same sample was measured repeatedly
☐	☒	The statistical test(s) used AND whether they are one- or two-sided<br>*Only common tests should be described solely by name; describe more complex techniques in the Methods section.*
☐	☒	A description of all covariates tested
☐	☒	A description of any assumptions or corrections, such as tests of normality and adjustment for multiple comparisons
☐	☒	A full description of the statistical parameters including central tendency (e.g. means) or other basic estimates (e.g. regression coefficient) AND variation (e.g. standard deviation) or associated estimates of uncertainty (e.g. confidence intervals)
☐	☒	For null hypothesis testing, the test statistic (e.g. *F*, *t*, *r*) with confidence intervals, effect sizes, degrees of freedom and *P* value noted<br>*Give P values as exact values whenever suitable.*
☒	☐	For Bayesian analysis, information on the choice of priors and Markov chain Monte Carlo settings
☒	☐	For hierarchical and complex designs, identification of the appropriate level for tests and full reporting of outcomes
☒	☐	Estimates of effect sizes (e.g. Cohen's *d*, Pearson's *r*), indicating how they were calculated

Our web collection on statistics for biologists contains articles on many of the points above.

Software and code

Policy information about availability of computer code

Data collection	Cell Proliferation data was collected using Incucyte® ZOOM v2016B (Satorius/Essen BioScience). Cell sorting was performed on FACSAria™ Fusions running DIVASoftware v8.0.1 (BD Biosciences). Illumina HumanOnmi2.5-8 arrays were used to assay genotype, DNA copy number, and loss of heterozygosity as described previously. Exome capture libraries were sequenced on HiSeq 2500 (Illumina, CA) to generate 75 million paired-end 75 base pair reads. RNA-seq libraries were multiplexed and sequenced on Illumina HiSeq2500 (Illumina) to generate 50 million paired-end 75 base pair reads. IHC stained slides were scanned on a NanoZoomer XR whole slide imager (Hamamatsu, Bridgewater NJ) at 200x magnification. Western blot data were acquired using the Odyssey Imager with Image Studio™ v5.2 (LI-COR). CellTiter-Glo luminescent assay for cell viability was measured on a Perkin Elmer/Wallac Envision Multilabel Reader using Wallac EnVision Manager software.
Data analysis	Differential expression analysis of RNA-seq data was performed using limma. For the hierarchical clustering and heatmap of RNA-seq transcriptome analysis, the RPKM values for the top 100 most variably expressed genes were z-scored and clustered using Euclidean distance. Somatic SNVs and INDELs were called by comparing the treatment resistant clones against the parental clones using LoFreq with its default setting. Highly-confident variants were annotated using Ensembl Variant Effect Predictor and filtered with dbSNP 138, ExAC 0.3.1 and RepeatMasker 4.0.5. The functional consequences of somatic variants were annotated using SIFT, PolyPhen and Condel. XLfit, IDBS software was used to determine IC50s for viability assays. Prism 9 (GraphPad) was used for statistical analysis for various in vitro experiments. Analysis and comparison of tumor growth was performed using a package of customized functions in R v3.6.2 (R Development Core Team 2008; R Foundation for Statistical Computing, Vienna, Austria) which integrate software from open source packages including lme4, mgcv, gamm4, multcomp, settings, plyr, and several packages from the tidyverse such as magrittr, dplyr, tidyr, and ggplot2. NanoZoomer XR whole slide imager (Hamamatsu, Bridgewater NJ) at 200x magnification were used to quantify the tumor and IHC positive staining areas. Segmentation of tumor regions and DAB positive pixels was performed by a custom algorithm using standard morphological operations and global RGB color thresholds running on Matlab 2019a (Mathworks, Natick, MA). Western blots acquired with LI-COR Odyssey Imager were analyzed with Image Studio™ v5.2 (LI-COR). Chemical genetics screen data was processed using Genedata Screener, Version 14 (Genedata; Basel, Switzerland).

For manuscripts utilizing custom algorithms or software that are central to the research but not yet described in published literature, software must be made available to editors and reviewers. We strongly encourage code deposition in a community repository (e.g. GitHub). See the Nature Portfolio guidelines for submitting code & software for further information.

Data

Policy information about availability of data

All manuscripts must include a data availability statement. This statement should provide the following information, where applicable:

- Accession codes, unique identifiers, or web links for publicly available datasets
- A description of any restrictions on data availability
- For clinical datasets or third party data, please ensure that the statement adheres to our policy

RNA-seq data that support the findings of this study have been deposited in Gene Expression Omnibus (GEO), accession number GSE139178, at https://www.ncbi.nlm.nih.gov/geo/query/acc.cgi?acc=GSE139178. Whole-exome sequencing data that support the findings of this study have been deposited in Sequence Read Archive (SRA), accession number PRJNA578183, at https://www.ncbi.nlm.nih.gov/sra/PRJNA578183.
Cancer genomics studies in which AKT1 W80 alterations were detected in patients were first identified using cBioPortal (http://www.cbioportal.org/index.do?session_id=5b5e1288498eb8b3d5672636). All AKT1 mutation information reported in those selected studies was then retrieved. The frequency of each AKT1 mutation detected within the same indication was calculated from these studies (# of patients harboring a specific AKT1 mutation/total # patients with that indication within the data set). Data bases used include TCGA: The Cancer Genome Atlas, https://portal.gdc.cancer.gov; METABRIC: Molecular Taxonomy of Breast Cancer International Consortium Nature 2012 & Nat Commun 2016), Pierra et al., 2016 https://www.ncbi.nlm.nih.gov/pubmed/27161491; MSK-IMPACT: Memorial Sloan Kettering Cancer Center's Integrated Mutation Profiling of Actionable Cancer Targets (MSKCC, Nat Med 2017).

Field-specific reporting

Please select the one below that is the best fit for your research. If you are not sure, read the appropriate sections before making your selection.

☒ Life sciences ☐ Behavioural & social sciences ☐ Ecological, evolutionary & environmental sciences

For a reference copy of the document with all sections, see nature.com/documents/nr-reporting-summary-flat.pdf

Life sciences study design

All studies must disclose on these points even when the disclosure is negative.

Sample size	No calculations were done to determine sample size. For in vivo experiments, animals were distributed into treatment groups at n=9/group. The number of animals per group was based on previous experience with such models and known to be sufficient to calculate precise estimates of tumor growth and treatment effect. For in vitro studies, sample size was determined following convention of the methods. In general, at least 3 biological replicates were performed to show data reproducibility.
Data exclusions	RNA-seq reads containing 30% or more bases with a Phred quality score of 23 or lower were excluded. Multimapping reads were discarded.
Replication	Each experiment was repeated at least 3 times unless otherwise indicated. Cell viability were tested in quadruplicate within each experiment.
Randomization	For in vitro experiments, samples were allocated randomly for culture and analysis. For in vivo studies, animals were distributed into treatment groups based on tumor volume following a non-random sequence tailored to result in groups with similarly-sized tumors by standard deviation.
Blinding	In general, the investigators were blind at the time of experiment execution and data acquisition. For in vivo studies, group allocation was achieved by an automated objective sequence not influenced by the study monitor and all other aspects of the study were conducted in compliance to our institutional ethical and integrity standards. Histological staining and immunohistochemistry was performed blinded and pathological assessment and digital quantification of the staining was blinded.

Reporting for specific materials, systems and methods

We require information from authors about some types of materials, experimental systems and methods used in many studies. Here, indicate whether each material, system or method listed is relevant to your study. If you are not sure if a list item applies to your research, read the appropriate section before selecting a response.

Materials & experimental systems

n/a	Involved in the study	
☐	☒	Antibodies
☐	☒	Eukaryotic cell lines
☒	☐	Palaeontology and archaeology
☐	☒	Animals and other organisms
☒	☐	Human research participants
☒	☐	Clinical data
☒	☐	Dual use research of concern

Methods

n/a	Involved in the study	
☒	☐	ChIP-seq
☒	☐	Flow cytometry
☒	☐	MRI-based neuroimaging

Antibodies

Antibodies used

Antibodies for Western blots (CST: Cell Signaling Technology):
AKT1 (C73H10) CST#2938 1:1000
AKT2 (D6G4) CST#3063 1:1000
AKT3 (L47B1) CST#8018 1:1000
Pan-AKT (40D4) CST#2920 1:1000
pAKT (T308) (C31E5E) CST#2965 1:1000
pAKT (S473) CST#9271 1:1000
BAD (D24A9) CST#9239 1:500
pBAD (S112) (7E11) CST#9296 1:500
4EBP1 CST#9452 1:1000
p4EBP1 (T37/46) (236B4) CST#2855 1:1000
p4EBP1 (S65) (174A9) CST#9456 1:1000
GSK3beta (3D10) CST#9832 1:500
pGSK3beta (S9) CST#9336 1:500
PARP (46D11) CST#9532 1:1000
Cleaved PARP (D64E10) (Asp214) CST#5625 1:1000
PIM1 Abnova#H00005292-M01 1:500
PIM2 (D1D2) CST#4730 1:500
PIM3 CST#4165 1:500
PRAS40 (D23C7) CST#2691 1:1000
PRAS40 (73P21) Invitrogen/ThermoFisher#AHO1031 1:1000
pPRAS40 (T246) (C77D7) CST#2997 1:1000
PTEN (26H9) CST#9556 1:1000
PTEN (138G6) CST#9559 1:1000
S6 (54D2) CST#2317 1:1000
pS6 (S235/236) CST#2211 1:1000
beta-Actin (AC-15) Sigma#A5441 1:3000
GAPDH (4G5) Advanced Immunochemical#2-RGM2 1:2000
beta-Tubulin Sigma#T8328 1:5000
IRDye 680RD Donkey anti-Rabbit IgG Secondary Antibody LI-COR#926-68073 1:10,000
IRDye 800CW Donkey anti-Mouse IgG (H + L) Secondary Antibody LI-COR#926-32212 1:10,000

Antibodies for IHC:
pAKT (S473) (D9E) CST#4060
pPRAS40 (T246) (C77D7) CST#2997
PTEN (138G6) CST#9559
pS6 (S235/236) CST#2211
Cleaved Caspase 3 (Asp175) CST#9661
Cyclin D1 (SP4) AbCam#ab16663

Validation

All of the antibodies used for Western blotting have been extensively used in the literature for previous studies, and validation blots are shown in the product information sheets on the manufacturer's websites, as detailed below:
Akt1 (C73H10) Rabbit mAb detects endogenous levels of total Akt1 protein. This antibody does not cross-react with Akt2 or Akt3. Species Reactivity: Human, Mouse, Rat, Monkey. Application: WB, IP.
Akt2 (D6G4) Rabbit mAb detects endogenous levels of total Akt2 protein. It does not cross-react with Akt1 or Akt3. Species Reactivity: Human, Mouse, Rat, Monkey. Application: WB, IP.
Akt3 (L47B1) Mouse mAb recognizes endogenous levels of total Akt3 protein. Species Reactivity: Human, Mouse, Rat, Hamster. Application: WB.
Akt (pan) (40D4) Mouse mAb detects endogenous levels of total Akt protein. This antibody does not cross-react with other related proteins. Species Reactivity: Human, Mouse, Rat, Monkey. Application: WB, IP, IHC.
Phospho-Akt (Thr308) (C31E5E) Rabbit mAb detects endogenous levels of Akt only when phosphorylated at Thr308. Species Reactivity: Human, Mouse, Rat, Hamster, Monkey. Application: WB.
Phospho-Akt (Ser473) Antibody detects endogenous levels of Akt1 only when phosphorylated at Ser473. This antibody also recognizes Akt2 and Akt3 when phosphorylated at the corresponding residues. It does not recognize Akt phosphorylated at other sites, nor does it recognize phosphorylated forms of related kinases such as PKC or p70 S6 kinase. Species Reactivity: Human, Mouse, Rat, Hamster, Monkey, D. melanogaster, Bovine, Dog. Species predicted to react based on 100% sequence homology: Monkey, Chicken, Xenopus, Horse. Application: WB, IP, IF, F.
Bad (D24A9) Rabbit mAb detects endogenous levels of total Bad protein. The antibody does not cross-react with related proteins. Species Reactivity: Human, Mouse, Rat, Monkey Species predicted to react based on 100% sequence homology: Bovine. Application: WB.
Phospho-Bad (Ser112) (7E11) Mouse mAb detects endogenous levels of Bad only when phosphorylated at serine112. The Ser112 nomenclature is based upon the mouse sequence. The analogous phosphorylation site is Ser75 in human and Ser113 in rat. This antibody does not detect Bad phosphorylated at other sites, nor does it detect related family members. Species Reactivity: Human, Mouse, Rat, Monkey. Application: WB.
4E-BP1 Antibody detects endogenous levels of total 4E-BP1, independent of phosphorylation. Species Reactivity: Human, Mouse, Rat, Monkey. Application: WB, IP.
Phospho-4E-BP1 (Thr37/46) (236B4) Rabbit mAb detects endogenous levels of 4E-BP1 only when phosphorylated at Thr37 and/or Thr46. This antibody may cross-react with 4E-BP2 and 4E-BP3 when phosphorylated at equivalent sites. Non-specific staining has been observed in mitotic cells by immunofluorescence. Species Reactivity: Human, Mouse, Rat, Monkey, D. melanogaster. Application: WB, IHC, IF, F.
Phospho-4E-BP1 (Ser65) (174A9) Rabbit mAb detects endogenous levels of 4E-BP1 when phosphorylated at Ser65. Species Reactivity: Human, Monkey. Species predicted to react based on 100% sequence homology: Chicken. Application: WB, IP.

GSK-3β (3D10) Mouse mAb recognizes endogenous levels of total GSK-3β protein. This antibody does not cross-react with GSK-3α. Species Reactivity: Human, Mouse, Rat, Hamster, Monkey. Application: WB, IP, IF, F.
Phospho-GSK-3beta (Ser9) Antibody detects endogenous levels of GSK-3β only when phosphorylated at serine 9. The antibody may cross-react weakly with the phosphorylated form of GSK-3α due to high sequence homology. Species Reactivity: Human, Mouse, Rat, Monkey. Species predicted to react based on 100% sequence homology: Zebrafish, Bovine. Application: WB.
PARP (46D11) Rabbit mAb detects endogenous levels of total full-length PARP-1 and the large fragment (89 kDa) produced by caspase cleavage at Asp214. This antibody does not cross-react with PARP-2 and PARP-3. Species Reactivity: Human, Mouse, Rat, Monkey. Application: WB, IP, IF, F.
Cleaved PARP (Asp214) (D64E10) XP® Rabbit mAb detects endogenous levels of the large fragment (89 kDa) of human PARP1 protein produced by caspase cleavage. The antibody does not recognize full length PARP1 or other PARP isoforms. Species Reactivity: Human, Monkey. Application: WB, IP, IHC, IF, F.
PIM1 Antibody (2C8) (H00005292-M02) Mouse Monoclonal Anti-PIM1 Antibody (2C8). Tested Applications: Western Blot, ELISA, ICC, Sandwich ELISA. Tested Reactivity: Human.
Pim-2 (D1D2) Rabbit mAb detects endogenous levels of total Pim-2 protein. The antibody does not cross-react with other Pim family members. Species Reactivity: Human. Application: WB, IP.
Pim-3 (D17C9) Rabbit mAb detects endogenous levels of total Pim-3 protein. It does not cross-react with other Pim family members. Species Reactivity: Human, Mouse, Rat. Species predicted to react based on 100% sequence homology: Monkey. Application: WB.
PRAS40 (D23C7) Rabbit mAb detects endogenous levels of total PRAS40 protein. Species Reactivity: Human, Mouse, Rat, Monkey. Application: WB, IP, IHC.
PRAS40 Monoclonal Antibody (73P21) This Antibody was verified by Cell treatment to ensure that the antibody binds to the antigen stated. Species Reactivity: Human. Application: WB, IP, IHC, IF.
Phospho-PRAS40 (Thr246) (C77D7) Rabbit mAb detects endogenous levels of PRAS40 protein only when phosphorylated at Thr246. Species Reactivity: Human, Mouse, Rat, Monkey. Application: WB, IP, IHC.
PTEN (26H9) Mouse mAb detects endogenous levels of PTEN protein. The antibody does not cross-react with related proteins.
S6 Ribosomal Protein (54D2) Mouse mAb detects endogenous levels of total S6 ribosomal protein independent of phosphorylation. Species Reactivity: Human, Mouse, Rat, Monkey, D. melanogaster. Application: WB, IHC, IF, F.
PTEN (138G6) Rabbit mAb detects endogenous levels of total PTEN protein. Species Reactivity: Human, Mouse, Rat, Monkey. Application: WB, IP, IHC.
Phospho-S6 Ribosomal Protein (Ser235/236) Antibody detects endogenous levels of ribosomal protein S6 only when phosphorylated at serine 235 and 236. This antibody does not detect ribosomal protein S6 phosphorylated at other sites. Species Reactivity: Human, Mouse, Rat, Monkey, S. cerevisiae. Species predicted to react based on 100% sequence homology: Chicken, Xenopus. Application: WB, IP, IHC, IF, F.
Monoclonal Anti-b-Actin Clone AC-15 recognizes an epitope located on the N-terminal end of the b-isoform of actin. The antibody specifically labels b-actin in a wide variety of tissues and species using immunoblotting (42 kDa), immunofluorescent staining of cultured cell lines, and immunohistochemistry. The antibody cross reacts with b-actin expressing cells in human, bovine, sheep, pig, rabbit, cat, dog, mouse, rat, guinea pig, chicken, carp, and leech tissues, but not in amoeba nor Drosophila.
Monoclonal Mouse Anti-rabbit GAPDH (4G5) Cross-reaction of MAbs in Western blotting with GAPDH from different animal species. Species Reactivity: Human, Bovine, Porcine, Goat, Cat, Mouse, Canine, Rabbit, Fish. Application: immunoassay, WB, ICC.
Mouse anti-β-tubulin antibody (AA2) reacts specifically with β tubulin, types I, II, III, and IV of bovine, rat, mouse and human. Species reactivity: rat, human, mouse, bovine. Application: WB, IP, IHC, ICC.

IHC staining was validated and scored by a pathologist using the antibodies listed below:
Phospho-Akt (Ser473) (D9E) XP® Rabbit mAb detects endogenous levels of Akt only when phosphorylated at Ser473. Species Reactivity: Human, Mouse, Rat, Hamster, Monkey, D. melanogaster, Zebrafish, Bovine. Species predicted to react based on 100% sequence homology: Chicken, Xenopus, Dog, Pig. Application: WB, IP, IHC, IF, F.
Phospho-PRAS40 (Thr246) (C77D7) Rabbit mAb detects endogenous levels of PRAS40 protein only when phosphorylated at Thr246. Species Reactivity: Human, Mouse, Rat, Monkey. Application: WB, IP, IHC.
PTEN (138G6) Rabbit mAb detects endogenous levels of total PTEN protein. Species Reactivity: Human, Mouse, Rat, Monkey. Application: WB, IP, IHC.
Phospho-S6 Ribosomal Protein (Ser235/236) Antibody detects endogenous levels of ribosomal protein S6 only when phosphorylated at serine 235 and 236. This antibody does not detect ribosomal protein S6 phosphorylated at other sites. Species Reactivity: Human, Mouse, Rat, Monkey, S. cerevisiae. Species predicted to react based on 100% sequence homology: Chicken, Xenopus. Application: WB, IP, IHC, IF, F.
Cleaved Caspase-3 (Asp175) Antibody detects endogenous levels of the large fragment (17/19 kDa) of activated caspase-3 resulting from cleavage adjacent to Asp175. This antibody does not recognize full length caspase-3 or other cleaved caspases. This antibody detects non-specific caspase substrates by western blot. Non-specific labeling may be observed by immunofluorescence in specific sub-types of healthy cells in fixed-frozen tissues (e.g. pancreatic alpha-cells). Nuclear background may be observed in rat and monkey samples. Species Reactivity: Human, Mouse, Rat, Monkey. Species predicted to react based on 100% sequence homology: Bovine, Dog, Pig. Application: WB, IP, IHC, IF, F.
Cyclin D1 (SP4) Rabbit monoclonal antibody. Species Reactivity: Mouse, Rat, Human. Tested and suitable for: ICC/IF, Flow Cyt (Intra), WB, IHC-P. Positive control: WB: MCF7, Hap1, A431 and HeLa, Nuero-2a, NIH/3T3, C6 cell lysates. IHC (FFPE): Human normal tonsil; breast carcinoma; mantle cell lymphoma; rat esophagus. ICC/IF: MCF7 cells, C6, Neuro-2a and HAP1 cells (HAP1-CCND1 knockout cells used as negative cell line). Flow Cyt (intra): MCF7, NIH/3T3 and C6 cells.

Eukaryotic cell lines

Policy information about cell lines

Cell line source(s)

The following cell lines are collected at Genentech's cell banking facility from the original sources in parentheses: the LNCaP (ATCC), HCT 116 (ATCC), PC-3 (ATCC), 22Rv1 (ATCC), DU 145 (ATCC), Ba/F3 (DSMZ). LNCaP G-Rpool, G-R1, G-R3, M-Rpool, M-R3, M-R7, Par X1.6, G-R3 X1.2, R0068 X1.2 are generated in this study.

Authentication

Cell lines were authenticated using short tandem repeat (STR) profiling and SNP fingerprinting by Genentech's cell banking facility.

Mycoplasma contamination	All stocks were tested for mycoplasma before and after cells were cryopreserved. All cell lines tested negative.
Commonly misidentified lines (See ICLAC register)	None

Animals and other organisms

Policy information about studies involving animals; ARRIVE guidelines recommended for reporting animal research

Laboratory animals	Male NOD scid gamma (NSG) mice (Jackson Laboratories), 6-8 weeks old; male SCID.bg C.B-17 mice (Charles River Labs), 6-8 weeks old.
Wild animals	none
Field-collected samples	none
Ethics oversight	All in vivo efficacy studies were approved by Genentech's Institutional Animal Care and Use Committee and adhere to the National Institutes of Health Guidelines for the Care and Use of Laboratory Animals.

Note that full information on the approval of the study protocol must also be provided in the manuscript.
